# Electrophotochemical metal-catalyzed synthesis of alkylnitriles from simple aliphatic carboxylic acids

**DOI:** 10.3762/bjoc.20.133

**Published:** 2024-07-03

**Authors:** Yukang Wang, Yan Yao, Niankai Fu

**Affiliations:** 1 Beijing National Laboratory for Molecular Sciences, CAS Key Laboratory of Molecular Recognition and Function, Institute of Chemistry, Chinese Academy of Sciences, Beijing 100190, China; 2 University of Chinese Academy of Sciences, Beijing 100049, China

**Keywords:** aliphatic carboxylic acids, alkylnitriles, electroorganic synthesis, electrophotocatalysis, radical decarboxylation

## Abstract

We report a practical and sustainable electrophotochemical metal-catalyzed protocol for decarboxylative cyanation of simple aliphatic carboxylic acids. This environmentally friendly method features easy availability of substrates, broad functional group compatibility, and directly converts a diverse range of aliphatic carboxylic acids including primary and tertiary alkyl acids into synthetically versatile alkylnitriles without using chemical oxidants or costly cyanating reagents under mild reaction conditions.

## Introduction

**Figure 1:**
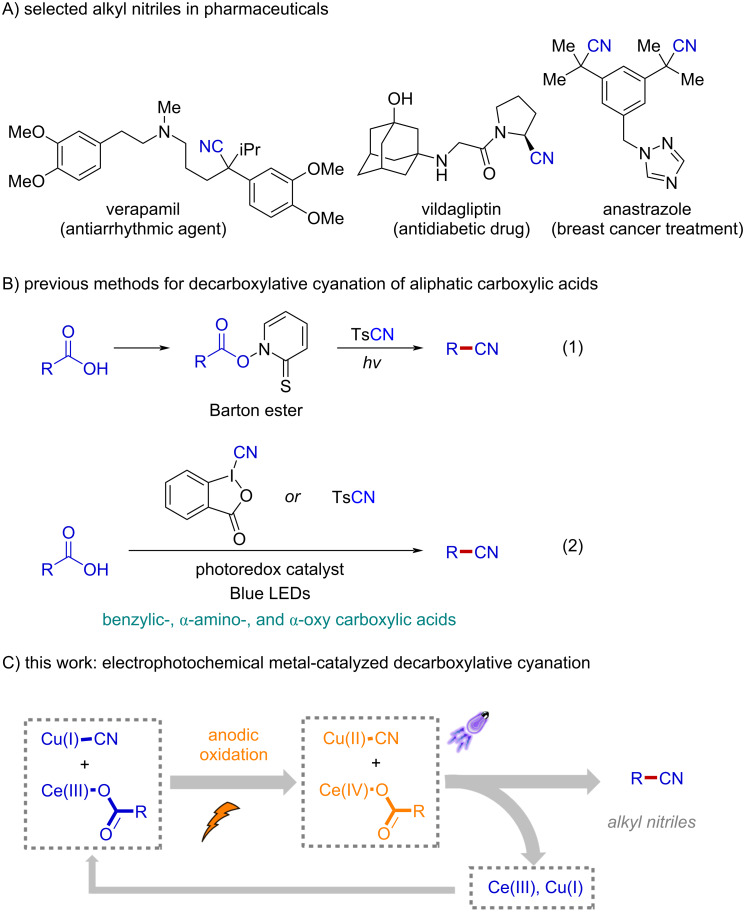
Decarboxylative cyanation: background and our working hypothesis.

Alkylnitriles and their derivatives are widely found in pharmaceuticals and biologically active compounds [1–3]. In addition, within the field of synthetic organic chemistry, nitriles are synthetically useful handles that can be readily converted into a myriad of functional groups including carbonyls, amines, imines, and a variety of heterocyclic scaffolds with well-established procedures [4–9]. In particular, tertiary nitriles are common structural motifs in many bioactive compounds and are widely used as intermediates in organic synthesis for the construction of all-carbon-substituted quaternary centers (Figure 1A). However, conventional methods for the synthesis of tertiary alkylnitriles such as direct functionalization of alkylnitriles [10] and hydrocyanation of alkenes [11–14] are typically hindered by harsh reaction conditions, which reduces functional group compatibility and product diversity. As such, the development of practical methods for the preparation of alkylnitriles from readily available starting materials are particularly valuable in synthetic and medicinal applications [15–18].

Owing to the prevalence of aliphatic carboxylic acids in biomass and natural products, decarboxylative cyanation represents one of the most straightforward and attractive approaches to accessing alkylnitriles [19–20]. As an elegant example, Barton demonstrated the application of redox-active esters, the so called "Barton esters", for decarboxylative cyanation of aliphatic acids with tosyl cyanide as the nitrile source under visible light irradiation at room temperature [21–22]. Although two synthetic steps are required, this is the first practical decarboxylative cyanation protocol because different types of aliphatic acids including primary ones could be successfully employed (Figure 1B, reaction 1). The groups of Waser [23] and Gonzalez-Gomez [24] reported the direct conversion of aliphatic acids to the corresponding alkylnitriles by merging photoredox catalysis and radical cyanation processes using cyanobenziodoxolones and tosyl cyanide as the cyanating reagents, respectively (Figure 1B, reaction 2). Recently, the Rueping group demonstrated a distinctive use of 4-cyanopyridine as nitrile source for electrochemical decarboxylative cyanation of amino acids [25]. Although these methods have provided innovative strategies, substrates in all of these reaction systems are generally limited to benzylic, α-amino-, and α-oxy aliphatic acids, presumably due to the necessity of stabilized radical intermediates for the following radical cyanation step.

We and others have recently demonstrated electrophotochemical transition metal catalysis [26–31] as a unique and powerful synthetic platform for radical decarboxylative functionalization of aliphatic carboxylic acids [32–37]. In particular, the commonly required high activation energy for radical decarboxylation was provided by anodic oxidation and visible light irradiation of the Ce species in a sequential fashion [38–45]. Therefore, the anodic electrode potential for this process could be substantially reduced. In doing so, a low working potential at the anode offers the opportunity for invention of cooperative catalysis with electrochemical transition metal catalysis, which generally has mild oxidation potential for the generation of persistent radicals in the form of nucleophile-bound metal complexes. We and other groups have successfully applied this reaction design to enantioselective decarboxylative cyanation of arylacetic acids [35–37]. Considering the widespread availability of aliphatic carboxylic acids and the significant synthetic and medicinal importance of alkylnitriles, we envisioned that the electrophotochemical Ce-catalyzed radical decarboxylation of alkyl carboxylic acids in combination with electrochemical copper catalysis might allow rapid access to alkylnitriles in a generic fashion (Figure 1C). Herein, we disclose the successful implementation of this strategy and present a mild, practical, and broadly applicable electrophotochemical metal-catalyzed protocol for the direct conversion of simple aliphatic carboxylic acids into alkylnitriles. Notably, this new decarboxylative cyanation protocol exhibited extraordinary insensitivity to substitution pattern of alkyl acids, affording the corresponding alkylnitriles including primary and tertiary alkylnitriles with good reaction efficiency.

## Results and Discussion

Our study of this new electrophotochemical metal-catalyzed decarboxylative cyanation commenced with the evaluation of various combinations of Ce and Cu catalysts. A simple undivided cell using a carbon felt, inexpensive and practical porous material as the anode, and a Pt plate as the cathode, was electrolyzed with a cell potential of 2.3 V (corresponding to an initial anodic potential of 0.10 V versus the ferrocenium ion/ferrocene redox couple) under the irradiation of 400 nm light-emitting diodes (LEDs). Through systematic optimization, we found that the use of readily available CeCl_3_ (10 mol %) and Cu(OTf)_2_ (5.0 mol %) together with bidentate nitrogen ligands such as BPhen, Phen, dtbbpy, and bpy with TMSCN as the cyanating reagent promoted the direct conversion of flurbiprofen (**1**) to the desired product (**2**) in good yields (Table 1, entries 1 and 2).

**Table 1:** Reaction discovery and optimization.^a^

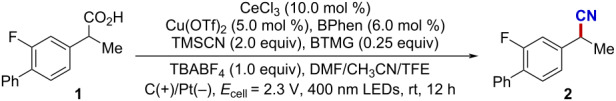		

Entry	Variations	Yield (%)

**1**	**none**	**88 (86)** ^b^
2	Phen, dtbbpy or bpy instead of BPhen	61–85
3	no BPhen	26
4	CH_3_CN as solvent	64^c^
5	3 mA for 4 h	86
6	no Ce catalyst	16
7	no Cu/BPhen catalyst	<5
8	no light	0
9	no electricity	0
10	[Mes-Acr]ClO_4_ instead of Ce	34

^a^Performed with **1** (0.2 mmol, 1.0 equiv) in DMF/CH_3_CN (1:7, 4.0 mL), TFE (2.5 equiv), carbon felt anode, Pt cathode, undivided cell, 400 nm LEDs. Yields determined by ^1^H NMR using 1,1,2,2-tetrachloroethane as the internal standard. ^b^Isolated yield. ^c^Due to the solubility issue of CeCl_3_ in CH_3_CN, Ce(OTf)_3_ was used instead, see Supporting Information File 1 for more details. BTMG, 2-*tert*-butyl-1,1,3,3-tetramethylguanidine. BPhen, bathophenanthroline. Phen, 1,10-phenanthroline, TFE, 2,2,2-trifluoroethanol.

Cu ions are well-known to be highly susceptible to electroplating on the cathode and thus require the use of ligands to avoid detrimental cathode deposition during electrolysis (Table 1, entry 3). In addition, we discovered that the additional use of DMF as co-solvent is beneficial to the reaction efficiency–reactions using acetonitrile as the solvent frequently led to the observation of Cu deposition at cathode (Table 1, entry 4). We reasoned that DMF could coordinate to the copper center, acting as a ligand to prevent copper from cathode reduction. Constant current electrolysis is also applicable to the reaction, the corresponding alkylnitrile product was obtained in 86% yield after electrolysis at 3.0 mA for 4 hours, demonstrating the high Faradaic efficiency of the reaction (Table 1, entry 5) [46]. Control experiments revealed that Ce catalyst, Cu catalyst, light, and electricity were all essential for the success of this transformation (Table 1, entries 6–9). We also tested other photoredox catalysts that are capable of driving the oxidative decarboxylation, only Fukuzumi catalyst [47] was able to deliver the product with a meaningful yield (Table 1, entry 10).

**Figure 2:**
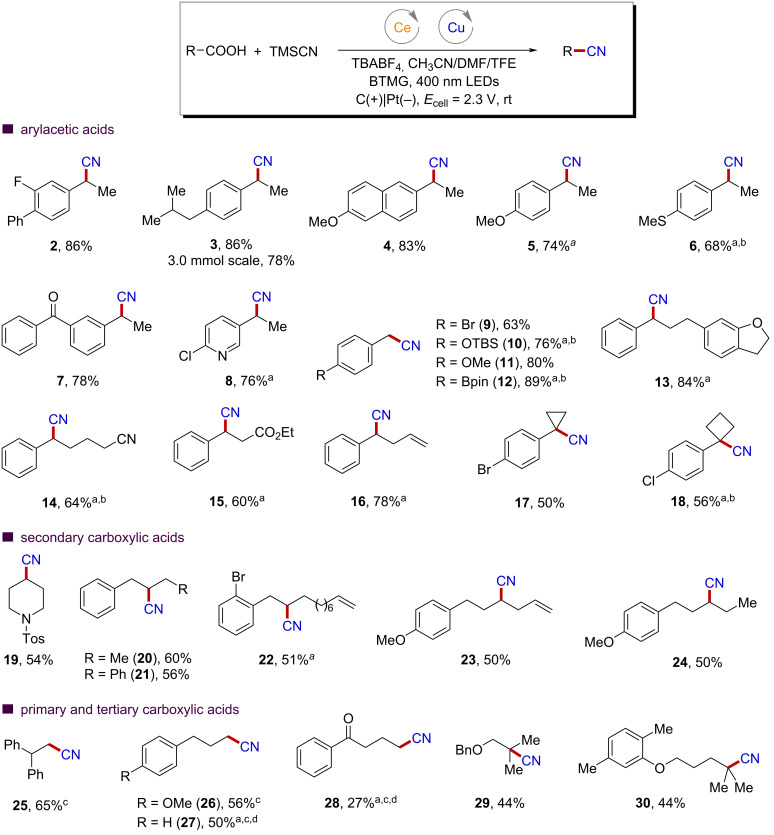
Scope of electrophotochemical decarboxylative cyanation of aliphatic carboxylic acids. All yields are of isolated products. Unless otherwise noted, reaction conditions were as follows: 0.2 mmol acids, 0.4 mmol TMSCN, CeCl_3_ (10 mol %), Cu(OTf)_2_/BPhen (5/6 mol %), 0.05 mmol BTMG, 0.2 mmol TBABF_4_, 0.5 mmol TFE, 3.5 mL of CH_3_CN, 0.5 mL of DMF, carbon felt as the anode, Pt as the cathode, under N_2_, in an undivided cell, at 2.3 V cell potential, 400 nm LEDs, for 12 hours. ^a^2,4,6-Collidine (1.0 equiv) was used instead of BTMG. ^b^Phen was used instead of BPhen. ^c^DMF/CH_3_CN (1:15 v/v) was used as solvent. ^d^Reactions were run with 0.4 mmol LiClO_4_ instead of TBABF_4_.

The scope of this transformation was next investigated (Figure 2). Arylacetic acids with relatively stable benzylic radicals as the corresponding intermediates have been proved to be suitable substrates to the reaction, providing the desired decarboxylative cyanation products with generally good yields (**2**–**18**). To show the synthetic potential of this method, we conducted the reaction with ibuprofen on a 3.0 mmol scale and obtained product **3** in 78% isolated yield. More importantly, the extremely mild reaction conditions imparted by the combination of electrochemistry and photochemistry made accessible a broad range of products with functionalities that are susceptible to oxidative degradation under traditional chemical conditions. For example, electron-rich arenes (**4**–**6**) can be smoothly obtained in synthetically useful yields. Electron-withdrawing groups at the phenyl ring are also compatible to give the products with good yields (**7**). Furthermore, the incorporation of pyridyl groups that are commonly found in pharmaceutically active compounds are also possible (**8**). In general, the catalytic efficiency of this new electrophotochemical protocol was found to be relatively independent of the electronic properties of the aryl substituents and the size of the alkyl side chain at the alpha position of arylacetic acids (**9**–**16**). These features offer great opportunities for the introduction of a wide range of functional groups, including bromide (**9**), boron (**12**), ether (**13**), nitrile (**14**), ester (**15**), and alkene (**16**) moieties, which are versatile functional handles for further elaboration. Notably, tertiary arylacetic acids can also be well tolerated to yield nitriles with quaternary carbon centers in good yields (**17** and **18**).

Simple carboxylic acids without functional groups at the alpha position to stabilize the corresponding carbon centered radicals are more challenging substrates. To our delight, both cyclic and acyclic secondary carboxylic acids performed well in our catalytic system, albeit with slightly reduced reaction efficiency (**19**–**24**). We also attempted simple primary carboxylic acids and got promising results. As outlined at the bottom of Figure 2, primary carboxylic acids can deliver the desired products with good yields in some cases (**25**–**27**). However, a large amount of hydrodecarboxylative products were observed, especially in the case of **28**. To our delight, tertiary carboxylic acids generally serve as better substrates (**29** and **30**). In these cases, a carbocation-involved pathway may be operative to yield the product. The successful and exclusive observation of product **29**, however, provided a piece of evidence to the objection of this possibility, as no carbocation-based rearrangement product was observed in our reaction system [48].

**Figure 3:**
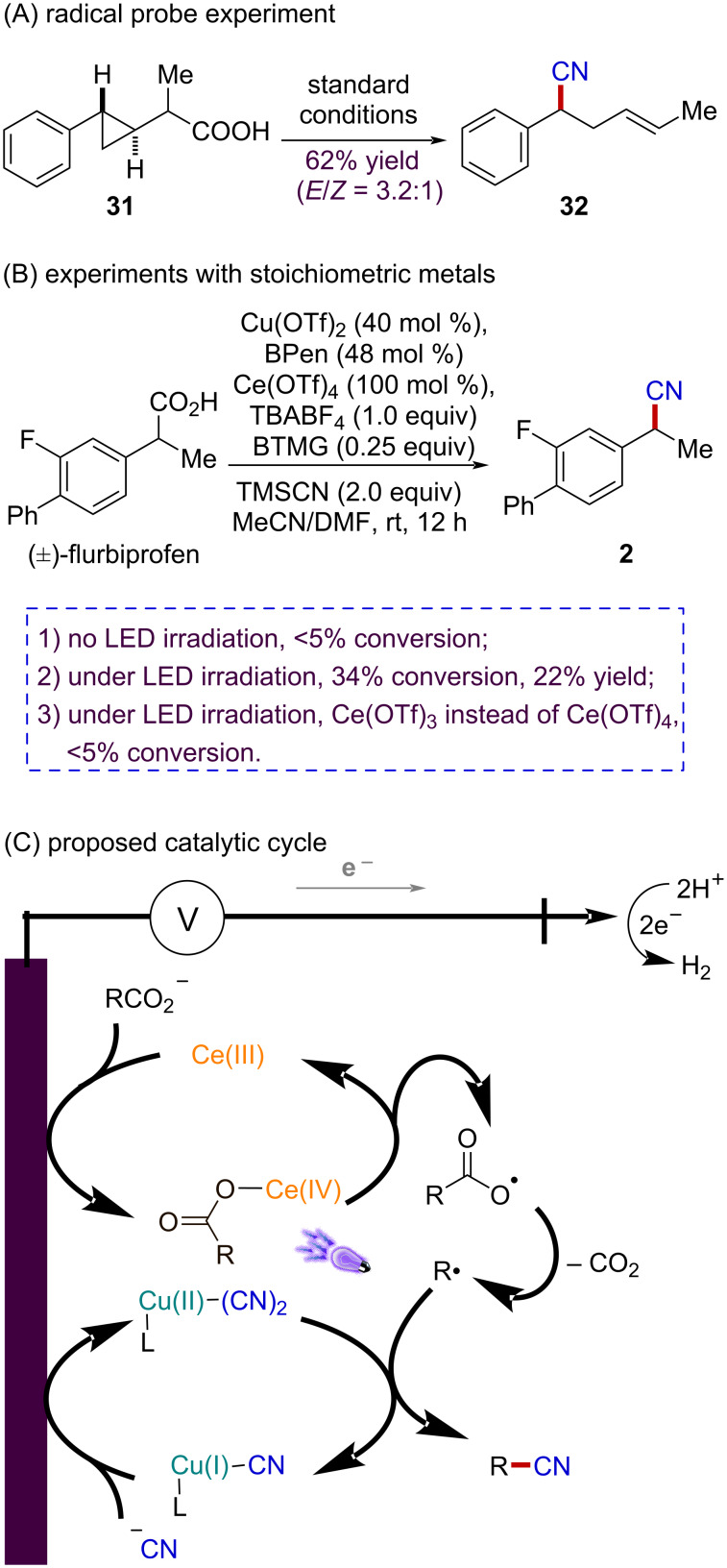
Mechanistic studies and proposed catalytic cycles.

To probe the radical intermediate in the reaction, a radical rearrangement experiment with cyclopropane-derived acid **31** was subjected to the standard conditions, leading to the expected ring opening, alkene-containing nitrile product **32** in 62% isolated yield (Figure 3A). Moreover, experiments using stoichiometric Cu(II) and Ce(IV) indicated that the radical decarboxylative cyanation reaction can only occur under light irradiation. In contrast, reaction with Ce(III) exhibited nearly no reactivity, demonstrating the crucial roles of anodic oxidation and light irradiation to the transformation (Figure 3B).

Collectively, our experimental observations are in agreement with the proposed mechanistic picture detailed in Figure 3C. The anodically generated Ce(IV) carboxylates are able to undergo homolytic cleavage of the Ce–O bond upon light irradiation. The resulting carboxyl radical would then extrude CO_2_ to generate the alkyl radical. Concurrently, Cu(II)–CN species are produced in the presence of cyanide anion through anodic oxidation. At this stage, Cu(II)–CN species are believed to capture alkyl radicals and the product would be readily generated via reductive elimination from the Cu(III) center [49–51].

## Conclusion

In summary, we have developed an efficient and practical protocol for the synthesis of alkylnitriles directly from readily available aliphatic carboxylic acids. The reaction proceeds under mild conditions and exhibits exceptional substrate generality and functional group compatibility and is applicable to alkyl acids with all substitution pattern. Due to the wide utility of alkylnitriles, we expect this method to be widely adopted within the synthetic and medicinal chemistry communities. The present work also demonstrated electrophotochemical transition metal catalysis as a viable and potentially general approach for reaction discovery and would find broad application in new synthetic contexts.

## Supporting Information

File 1 Experimental procedures, mechanistic studies, analytical data and copies of NMR spectra.

## Data Availability

All data that supports the findings of this study is available in the published article and/or the supporting information to this article.
