# Supplementary material for: Electrophotochemical metal-catalyzed synthesis of alkylnitriles from simple aliphatic carboxylic acids
Source: Beilstein J Org Chem. 2024 Jul 3;20:1497–503. doi: 10.3762/bjoc.20.133 (PMC11228820; doi:10.3762/bjoc.20.133)

BEILSTEIN JOURNAL OF ORGANIC CHEMISTRY

Supporting Information

for

Electrophotochemical metal-catalyzed synthesis of alkylnitriles from simple aliphatic carboxylic acids

Yukang Wang, Yan Yao and Niankai Fu

Beilstein J. Org. Chem. **2024,** *20,* 1497–1503. doi:10.3762/bjoc.20.133

Experimental procedures, mechanistic studies, analytical data and copies of NMR spectra

License and Terms: This is a supporting information file under the terms of the Creative Commons Attribution License (https://creativecommons.org/licenses/by/4.0). Please note that the reuse, redistribution and reproduction in particular requires that the author(s) and source are credited and that individual graphics may be subject to special legal provisions.
The license is subject to the *Beilstein Journal of Organic Chemistry* terms and conditions: (https://www.beilstein-journals.org/bjoc/terms)

Table of contents

1. Material and methods....S2
2. Experimental procedures....S3
3. Mechanistic studies....S11
4. Spectral data....S13
5. References....S31
6. NMR Spectra....S33

1. Material and methods

Unless stated otherwise, reactions were performed in oven-dried glassware under positive pressure of nitrogen atmosphere. Acetonitrile and dimethylformamide were purchased from commercial sources and used as received. Tetrahydrofuran was distilled over sodium with benzophenone as the indicator. Reagents obtained from commercial sources were used as supplied unless stated otherwise. $CeCl_3$ and $Cu(OTf)_2$ that were frequently used and stored on benchtop remained equally effective after at least one ($CeCl_3$) and four months ($Cu(OTf)_2$). All decarboxylative cyanation reactions were set up outside a glove box. Thin-layer chromatography (TLC) was performed on pre-coated plates, silica gel 60 PF_{254} (0.25 mm). TLC were visualized with UV light (254 nm) or stained using $KMnO_4$. Flash chromatography was performed on silica gel 60 (200–400 mesh). ^{1}H NMR spectra were recorded on a Bruker Avance (400 or 500 MHz) spectrometer using $CDCl_3$ as solvent and referenced relative to tetramethylsilane (δ = 0.00 ppm). Chemical shifts are reported in ppm and coupling constants (*J*) in Hertz. ^{13}C NMR spectra were recorded on the same instruments (101 or 126 MHz) with total proton decoupling referenced relative to $CDCl_3$ (δ = 77.16 ppm). Infrared spectra were obtained on Thermo Fisher Nicolet 6700. High-resolution mass spectra were recorded on commercial instruments (APCI or ESI). Cyclic voltammetry spectra were recorded on Shanghai Chenhua CHI660E.

2. Experimental procedures

General procedure for decarboxylative cyanation – Reaction optimization and substrate scope study (Method A):

Stock solutions: All stock solutions were prepared using a mixture of MeCN and DMF (7:1 v/v) as the solvent, which was degassed by sparging with nitrogen gas. Stoichiometries of reagents were given in regard to a reaction at 0.20 mmol scale. Solutions A, B, C, and D were freshly prepared prior to use. The purpose of using stock solutions was to speed up setting up multiple reactions at the same time and to increase the accuracy of adding catalysts at small quantities.

Solution A: TFE (36 μL, 0.5 mmol, 2.5 equiv) and BTMG (10 μL, 0.05 mmol, 0.25 equiv) per milliliter.

Solution B: $Cu(OTf)_2$ (3.6 mg, 0.01 mmol, 5 mol %) and bathophen (4.0 mg, 0.012 mmol, 6 mol %) per milliliter.

Solution C: $CeCl_3$ (4.9 mg, 0.02 mmol, 10 mol %) per milliliter.

Solution D: TMSCN (50 μL, 0.40 mmol, 2.0 equiv) per milliliter.

Reaction setup: An oven-dried two-neck tube was equipped with a stir bar, a rubber septum, a threaded Teflon cap fitted with electrical feedthroughs, a carbon felt anode (1.2 × 0.8 cm^2, connected to the electrical feedthrough via a 9 cm in length, 2 mm in diameter graphite rod), and a platinum plate cathode (0.5 × 1.0 cm^2). $TBABF_4$ (66 mg, 0.20 mmol, 1.0 equiv) and carboxylic acid substrate (0.20 mmol, 1.0 equiv) were added followed by subsequent addition of solutions A, B, C, and D (1 mL each) under the atmosphere of nitrogen. The reaction mixture was then sparged with nitrogen for 5 minutes and maintained under nitrogen atmosphere with a balloon. The reaction was irradiated with LEDs (10 W, 400 nm) under the vessel and electrolysis was initiated at a constant cell potential of 2.30 V. After 12 hours at room temperature, the photolysis and electrolysis were terminated. The reaction mixture was then transferred to a round bottom flask using EtOAc/petroleum ether (1:1) and quenched with water (ca. 0.8 mL). The mixture was

filtered through a short silica gel plug using EtOAc/petroleum ether (1:1) and the filtrate was concentrated under reduced pressure. The crude material was purified by column chromatography on silica gel to give the pure product.

NOTE: For optimization studies, 1 M HCl was used to quench the reaction so that conversion rates could be measured. Although most of cyanide anions were consumed, small quantity of TOXIC HCN could potentially form during this acidic work-up. It should be handled with **extreme caution**! Concentrating inside a fume hood is recommended for safety purposes.

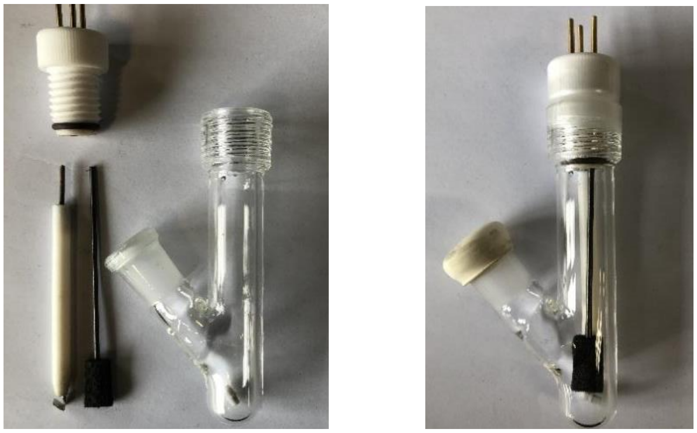

Figure S1. Components of the reaction setup (left) and assembled reaction vessel (right).

Table S1. Reaction discovery and optimization[a]

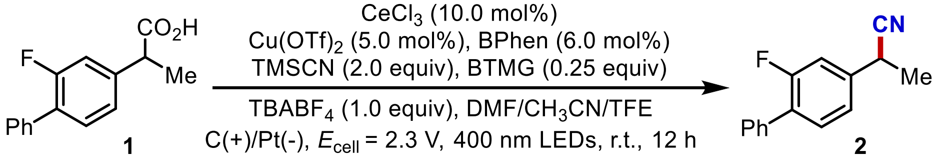

entry	variation from above conditions	conversion (%)	yield of **2** (%)
1	none	100	**88 (86)**[b]
2	phen instead of BPhen	76	61
3	bpy instead of BPhen	100	85
4	dtbbpy instead of BPhen	100	84
5	no BPhen	74	26
6[c]	CH_3CN as solvent	100	64
7	5.0 equiv TFE	100	84
8	water instead of TFE	78	50
9	No BTMG	54	26
10	2,4,6-collidine instead of BTMG	78	70
11	no Ce catalyst	50	16
12	no Cu/BPhen catalyst	38	<5
13	no light	<5	0
14	no electricity	10	0
15	Carbon felt as cathode	>95	50
16	3.0 mA for 4 hours	100	86
17	4CzIPN instead of $CeCl_3$	34	6
18	[Mes-Acr]ClO_4 instead of $CeCl_3$	>95	34

[a]Reaction conditions: **1** (0.2 mmol, 1.0 equiv) in DMF/CH_3CN (1:7, 4.0 mL), TFE (2.5 equiv), carbon felt anode, Pt cathode, undivided cell, 400 nm LEDs. Yields determined by ^{1}H NMR using 1,1,2,2-tetrachloroethane as the internal standard. [b]Isolated yield. [c]Due to the solubility issue of $CeCl_3$ in CH_3CN, $Ce(OTf)_3$ was used instead. BTMG, 2-*tert*-butyl-1,1,3,3-tetramethylguanidine. BPhen, bathophenanthroline. Phen, 1,10-phenanthroline, TFE, 2,2,2-trifluoroethanol.

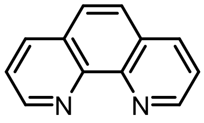

phen

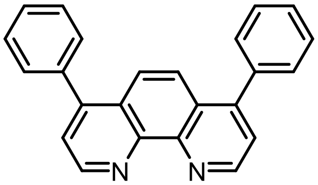

BPhen

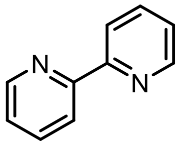

bpy

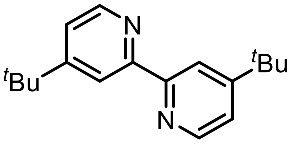

dtbbpy

Table S2. Ligand Screening using 4-(4-methoxyphenyl)butanoic acid[a]

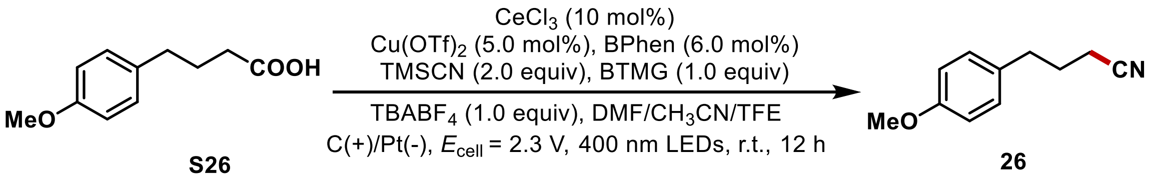

entry	variations	conversion (%)	yield of **26** (%)
1	none	36	26
2	phen	45	21
3	L1	23	14
4	L2	38	6
5	L3	40	trace
6	bpy	26	15
7	dtbbpy	50	20

[a]Reaction conditions: **S26** (0.2 mmol, 1.0 equiv), DMF/CH_3CN (1:7, 4.0 mL), TFE (2.5 equiv), carbon felt anode, Pt cathode, undivided cell, 400 nm LEDs. Yields determined by ^{1}H NMR using 1,1,2,2-tetrachloroethane as the internal standard.

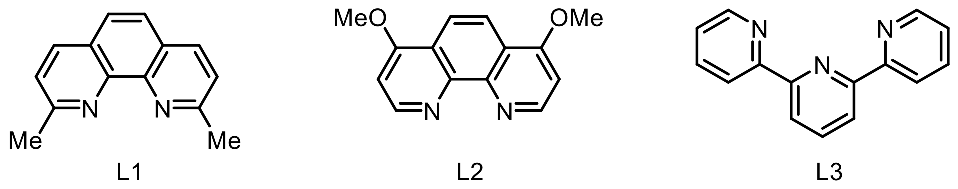

Table S3. Other conditions[a]

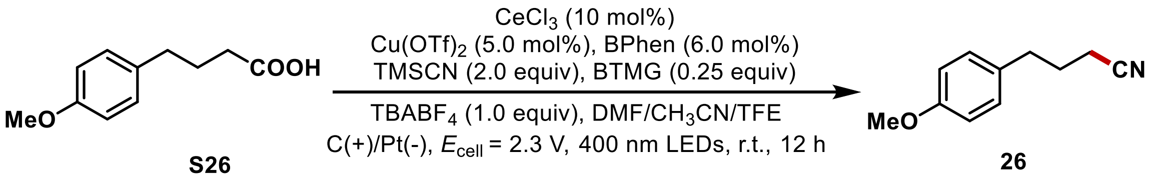

entry	variations	conversion (%)	yield of **26** (%)
1	none	48	44
3	0.50 equiv BTMG	46	36
2	1.0 equiv BTMG	45	21
4	DMF/CH_3CN (1:3 v/v)	38	21
**5**	**DMF/CH_3CN (1:15 v/v)**	**68**	**56**[b]

[a]Reaction conditions: **S26** (0.2 mmol, 1.0 equiv), DMF/CH_3CN (1:7, 4.0 mL), TFE (2.5 equiv), carbon felt anode, Pt cathode, undivided cell, 400 nm LEDs. Yields determined by ^1H NMR using 1,1,2,2-tetrachloroethane as the internal standard. [b]Isolated yield.

Decarboxylative cyanation of ibuprofen – Scale-up synthesis:

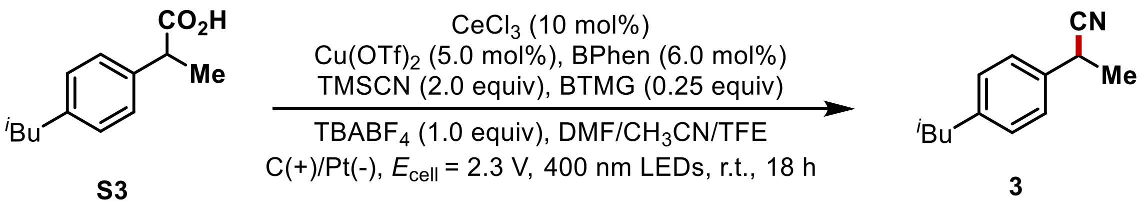

MeCN/DMF (7:1 v/v) was used as the solvent. It was rigorously degassed with 'freeze-pump-thaw' technique prior to use.

An oven-dried two-neck tube (5.0 cm in diameter, 12.0 cm in length) was equipped with a stir bar, a rubber septum, a threaded Teflon cap fitted with electrical feedthroughs, a carbon felt anode (2.0 × 3.0 cm^2), and a platinum plate cathode (2.0 × 3.0 cm^2). The electrodes were connected to the electrical feedthrough, each via a 9 cm in length, 2 mm in diameter graphite rod. To the flask was added $TBABF_4$ (990 mg, 3.0 mmol, 1.0 equiv), $CeCl_3$ (74.0 mg, 0.300 mmol, 10 mol %), ibuprofen (**S3**, 618 mg, 3.0 mmol, 1.0 equiv), $Cu(OTf)_2$ (54.3 mg, 0.15 mmol, 5 mol %) and bathophen (59.8 mg, 0.18 mmol, 6 mol%). The flask was then evacuated and refilled with nitrogen (3×). Solvent (60 mL), TFE (0.53 mL, 7.5 mmol, 2.5 equiv), BTMG (0.15 mL, 0.75 mmol, 0.25 equiv) and TMSCN (0.75 mL, 6.0 mmol, 2.0 equiv) were added subsequently. The reaction was irradiated with two Kessil lamps (Model PR160L-390 nm, both at 100% intensity, 40 W each, 6 cm away), and electrolysis was initiated at a constant voltage of 2.3 V. After 18 hours at room temperature, the photolysis and electrolysis were terminated. The reaction was quenched with 0.5 M HCl. The two layers were separated, and the aqueous layer was extracted with EtOAc (3×). The combined organic layers were washed with water (2×) and brine, dried over Na_2SO_4 and concentrated under reduced pressure. The crude material was purified by column chromatography on silica gel (EtOAc/petroleum ether 1/20 to 1/10) to furnish cyanation **3** (440 mg) as a colorless oil in 78% yield.

NOTE: TOXIC HCN could potentially form during the acidic work-up. It should be handled with **extreme caution**! Concentrating inside a fume hood is recommended for safety purposes.

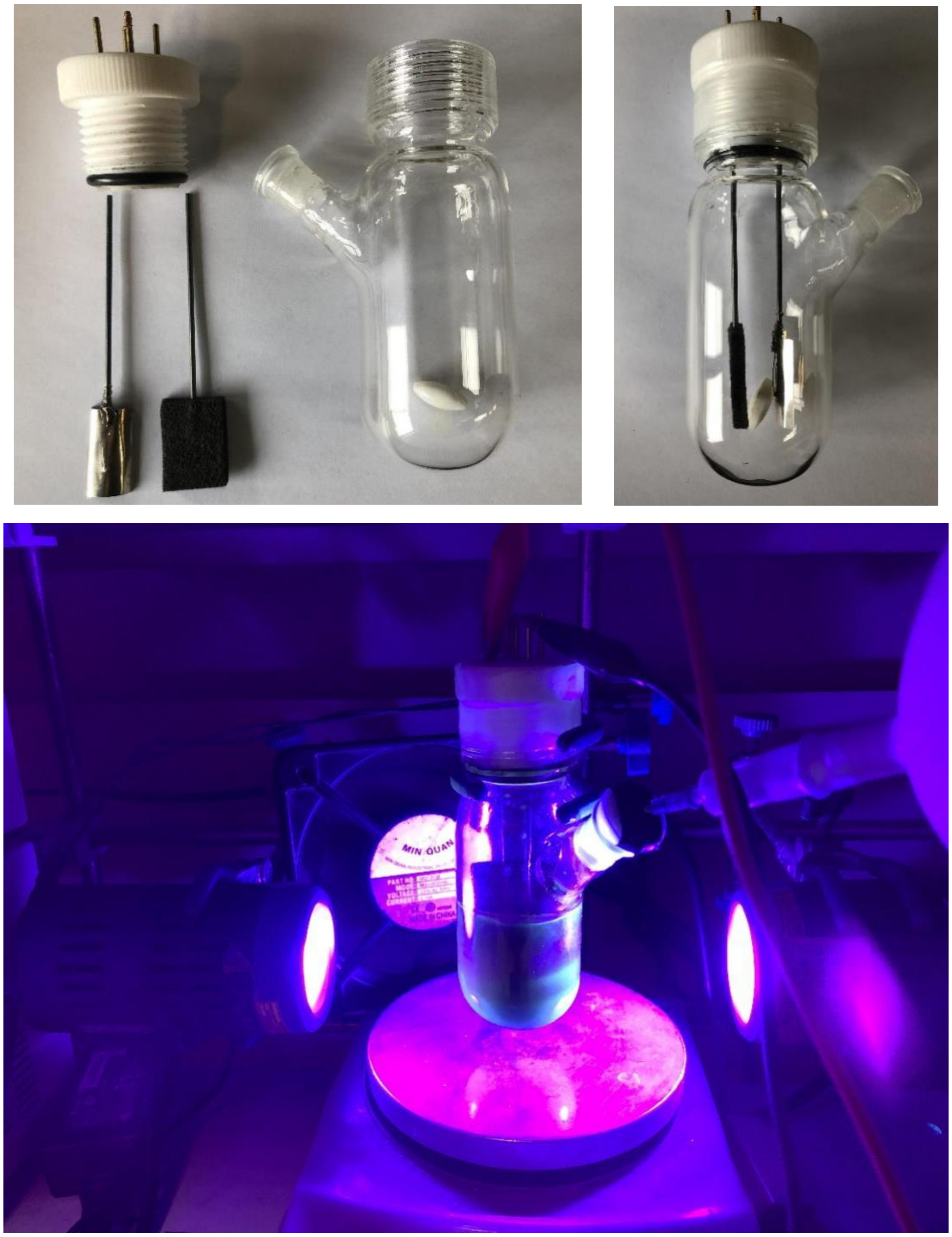

Figure S2. Components of the reaction setup (top left). Assembled reaction vessel (top right). Typical reaction setup/appearance for scale-up synthesis (bottom).

3. Mechanistic studies

3.1 Radical clock experiment:

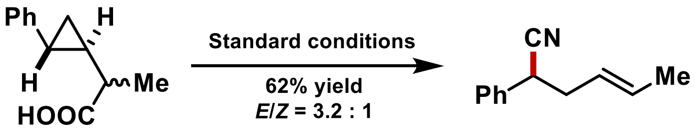

31 32

Cyclopropylpropanoic acid **31** was subjected to the standard conditions and reacted for 12 hours. Ring opening product **32** was isolated as a mixture of geometric isomers in 62% yield.

3.2 Experiments using stoichiometric Ce(IV) and Cu(II)/BPhen:

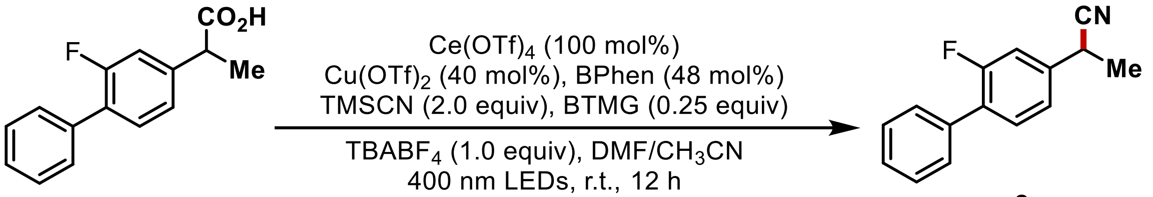

1 2

1) no LED irradiation, < 5% conversion;
2) under LED irradiation, 34% conversion, 22% yield;
3) under LED irradiation, $Ce(OTf)_3$ instead of $Ce(OTf)_4$, < 5% conversion.

Three reactions were set up in parallel. To an oven-dried two-neck tube was added $TBABF_4$ (33 mg, 0.10 mmol, 1.0 equiv), flurbiprofen (24.4 mg, 0.10 mmol, 1.0 equiv), BTMG (4.3 mg, 0.025 mol, 0.25 equiv), $Ce(OTf)_4$ (58.7 mg, 0.10 mmol, 1.0 equiv), $Cu(OTf)_2$ (14.4 mg, 0.04 mmol, 0.4 equiv) and bathophen (15.9 mg, 0.048 mmol, 0.48 equiv). The tube was evacuated and refilled with nitrogen (3×). Degassed MeCN/DMF (7:1 v/v, 3.0 mL in total) was added followed by TMSCN (25 μL). One reaction was irradiated with LEDs (10 W, 400 nm) under the vessel, while the other one was stirred in dark, the third one using $Ce(OTf)_3$ (29 mg, 0.10 mmol, 1.0 equiv) instead of $Ce(OTf)_4$. After 12 hours at room temperature, three reactions were worked up separately. The reaction was quenched with 1 M HCl and extracted with EtOAc (3×). The organic layers were washed

with water and brine, dried over Na_2SO_4 and concentrated. The residue was passed through a short plug of silica gel (EtOAc/petroleum ether 1:1). The filtrate was concentrated under reduced pressure. Conversion were determined by ^1H NMR spectra using 1,1,2,2-tetrachloroethane as the internal standard. Yields are isolated yield.

4. Spectral data

2-(2-Fluoro-[1,1'-biphenyl]-4-yl)propanenitrile (2)

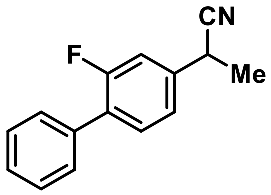

Synthesized following general method A from flurbiprofen. 38.7 mg isolated (86%).

Colorless oil.

R$_f$ = 0.6 (petroleum ether/EtOAc 5:1)

^{1}H NMR (400 MHz, CDCl$_3$): δ = 7.55 – 7.53 (m, 2H), 7.49 – 7.44 (m, 3H), 7.41 – 7.37 (m, 1H), 7.24 – 7.16 (m, 2H), 3.94 (q, J = 7.2 Hz, 1H), 1.69 (d, J = 7.3 Hz, 3H).

^{13}C NMR (101 MHz, CDCl$_3$): δ = 160.0, 159.0, 138.3 (d, $J_{C\text{-}F}$ = 6.1 Hz), 135.1, 131.6 (d, $J_{C\text{-}F}$ = 3.0 Hz), 129.1 (d, $J_{C\text{-}F}$ = 3.0 Hz), 128.7, 128.1, 122.8 (d, $J_{C\text{-}F}$ = 3.0 Hz), 121.1, 114.8 (d, $J_{C\text{-}F}$ = 19.2 Hz), 30.9, 21.3.

^{19}F NMR (377 MHz, CDCl$_3$): δ = -116.3.

The spectral data are consistent with those reported in the literature.[1]

2-(4-Isobutylphenyl)propanenitrile (3)

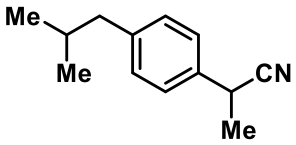

Synthesized following general method A from ibuprofen. 33 mg isolated (86%).

Colorless oil.

R$_f$ = 0.6 (petroleum ether/EtOAc 10:1)

^{1}H NMR (400 MHz, CDCl$_3$): δ = 7.25 (d, J = 8.0 Hz, 2H), 7.15 (d, J = 8.0 Hz, 2H), 3.87 (q, J = 7.3 Hz, 1H), 2.47 (d, J = 7.2 Hz, 2H), 1.91 – 1.78 (m, 1H), 1.63 (d, J = 7.3 Hz, 3H), 0.90 (d, J = 6.6 Hz, 6H).

^{13}C NMR (101 MHz, CDCl$_3$): δ = 141.8, 134.4, 129.9, 126.6, 121.9, 45.1, 31.0, 30.3, 22.4, 21.6.

The spectral data are consistent with those reported in the literature.[1]

2-(6-Methoxynaphthalen-2-yl)propanenitrile (4)

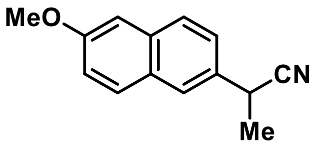

Synthesized following general method A from naproxen. 35 mg isolated (83%).

White solid.

R$_f$ = 0.6 (petroleum ether/EtOAc 5:1)

^{1}H NMR (400 MHz, CDCl$_3$): δ = 7.75 (t, J = 9.7 Hz, 3H), 7.42 – 7.36 (m, 1H), 7.21 – 7.16 (m, 1H), 7.16 – 7.11 (m, 1H), 4.03 (q, J = 7.3 Hz, 1H), 3.93 (s, 3H), 1.71 (d, J = 7.3 Hz, 3H).

^{13}C NMR (101 MHz, CDCl$_3$): δ = 158.2, 134.1, 132.1, 129.4, 128.9, 128.0, 125.5, 125.1, 121.9, 119.7, 105.8, 55.5, 31.4, 21.6.

The spectral data are consistent with those reported in the literature.[1]

2-(4-Methoxyphenyl)propanenitrile (5)

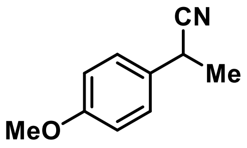

Synthesized following general method A using 2,4,6-collidine (1.0 equiv) instead of BTMG (0.25 equiv). 24 mg isolated (74%).

Colorless oil.

R$_f$ = 0.5 (petroleum ether/EtOAc 5:1)

^{1}H NMR (500 MHz, CDCl$_3$): δ = 7.27 (d, J = 8.2 Hz, 2H), 6.90 (d, J = 8.6 Hz, 2H), 3.90 – 3.77 (m, 4H), 1.62 (d, J = 7.3 Hz, 3H).

^{13}C NMR (126 MHz, CDCl$_3$): δ = 159.6, 129.3, 128.0, 122.0, 114.7, 55.5, 30.6, 21.7.

The spectral data are consistent with those reported in the literature.[1]

2-(4-(Methylthio)phenyl)propanenitrile (6)

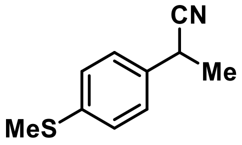

Synthesized following general method A using 2,4,6-collidine (1.0 equiv) instead of BTMG (0.25 equiv) and phen instead of bathophen. 24 mg isolated (68%).

Colorless oil.

R$_f$ = 0.5 (petroleum ether/EtOAc 5:1)

^{1}H NMR (400 MHz, CDCl$_3$): δ = 7.30 – 7.22 (m, 4H), 3.86 (q, J = 7.3 Hz, 1H), 2.48 (s, 3H), 1.62 (d, J = 7.3 Hz, 3H).

^{13}C NMR (101 MHz, CDCl$_3$): δ = 138.8, 133.8, 127.3, 127.2, 121.6, 30.9, 21.5, 15.8.

The spectral data are consistent with those reported in the literature.[2]

2-(3-Benzoylphenyl)propanenitrile (7)

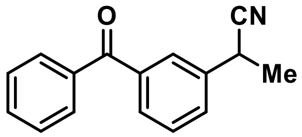

Synthesized following general method A from ketoprofen. 36.7 mg isolated (78%).

Colorless oil.

R$_f$ = 0.5 (petroleum ether/EtOAc 5:1)

^{1}H NMR (400 MHz, CDCl$_3$): δ = 7.84 – 7.76 (m, 3H), 7.76 – 7.69 (m, 1H), 7.67 – 7.57 (m, 2H), 7.57 – 7.45 (m, 3H), 3.98 (q, J = 7.3 Hz, 1H), 1.68 (d, J = 7.3 Hz, 3H).

^{13}C NMR (101 MHz, CDCl$_3$): δ = 196.1, 138.7, 137.7, 137.2, 132.9, 130.7, 130.2, 130.0, 129.3, 128.6, 128.3, 121.2, 31.3, 21.4.

Spectral data for this compound were consistent with those in the literature.[1]

2-(6-Chloropyridin-3-yl)propanenitrile (8)

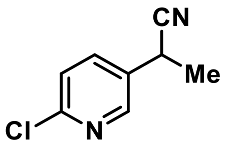

Synthesized following general method A using 2,4,6-collidine (1.0 equiv) instead of BTMG (0.25 equiv). 25 mg isolated (76%).

Colorless oil.

R_f = 0.4 (petroleum ether/EtOAc 5:1)

^{1}H NMR (400 MHz, $CDCl_3$): δ = 8.41 – 8.35 (m, 1H), 7.76 – 7.64 (m, 1H), 7.48 – 7.34 (m, 1H), 3.95 (q, J = 7.3 Hz, 1H), 1.67 (d, J = 7.3 Hz, 3H).

^{13}C NMR (101 MHz, $CDCl_3$): δ = 151.6, 148.2, 137.2, 132.0, 124.9, 120.2, 28.5, 21.3.

Spectral data for this compound were consistent with those in the literature.[1]

2-(4-Bromophenyl)acetonitrile (9)

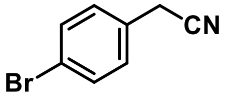

Synthesized following general method A. 25 mg isolated (63%).

Colorless oil.

R_f = 0.6 (petroleum ether/EtOAc 5:1)

^{1}H NMR (400 MHz, $CDCl_3$): δ = 7.55 – 7.49 (m, 2H), 7.24 – 7.18 (m, 2H), 3.71 (s, 2H).

^{13}C NMR (101 MHz, $CDCl_3$): δ = 132.5, 129.7, 129.0, 122.4, 117.4, 23.4.

Spectral data for this compound were consistent with those in the literature.[3]

2-(4-((*tert*-Butyldimethylsilyl)oxy)phenyl)acetonitrile (10)

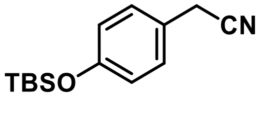

Synthesized following general method A using 2,4,6-collidine (1.0 equiv) instead of BTMG (0.25 equiv) and phen instead of bathophen. 37.5 mg isolated (76%).

Colorless oil.

R_f = 0.5 (petroleum ether/EtOAc 5:1)

^{1}H NMR (500 MHz, $CDCl_3$): δ = 7.17 – 7.09 (m, 2H), 6.83 – 6.76 (m, 2H), 3.57 (s, 2H), 0.98 (s, 9H), 0.19 (s, 6H).

^{13}C NMR (126 MHz, $CDCl_3$): δ = 178.1, 155.1, 130.5, 126.1, 120.3, 40.4, 25.8, 18.3, -4.29.

Spectral data for this compound were consistent with those in the literature.[4]

2-(4-Methoxyphenyl)acetonitrile (11)

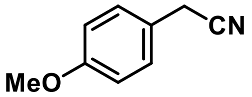

Synthesized following general method A. 23.5 mg isolated (80%).

Colorless oil.

R_f = 0.4 (petroleum ether/EtOAc 5:1)

^{1}H NMR (500 MHz, $CDCl_3$): δ = 7.26 – 7.20 (m, 2H), 6.93 – 6.86 (m, 2H), 3.81 (s, 3H), 3.68 (s, 2H).

^{13}C NMR (126 MHz, $CDCl_3$): δ = 159.5, 129.2, 121.9, 118.3, 114.7, 55.5, 22.9.

Spectral data for this compound were consistent with those in the literature.[3]

2-(4-(4,4,5,5-Tetramethyl-1,3,2-dioxaborolan-2-yl)phenyl)acetonitrile (12)

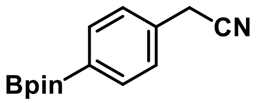

Synthesized following general method A using 2,4,6-collidine (1.0 equiv) instead of BTMG (0.25 equiv) and phen instead of bathophen. 42 mg isolated (89%).

White solid.

R_f = 0.5 (petroleum ether/EtOAc 5:1)

^{1}H NMR (500 MHz, $CDCl_3$): δ = 7.86 – 7.76 (m, 2H), 7.38 – 7.29 (m, 2H), 3.77 (s, 2H), 1.35 (s, 12H).

^{13}C NMR (126 MHz, $CDCl_3$): δ = 135.7, 132.9, 127.4, 117.8, 84.2, 25.0, 23.9.

Spectral data for this compound were consistent with those in the literature.[5]

4-(2,3-Dihydrobenzofuran-6-yl)-2-phenylbutanenitrile (13)

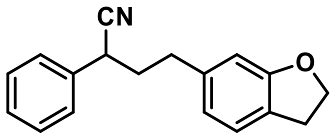

Synthesized following general method A using 2,4,6-collidine (1.0 equiv) instead of BTMG (0.25 equiv). 44.2 mg isolated (84%).

Colorless oil.

R_f = 0.4 (petroleum ether/EtOAc 5:1)

^{1}H NMR (400 MHz, $CDCl_3$): δ = 7.44 – 7.28 (m, 5H), 7.04 (s, 1H), 6.95 – 6.88 (m, 1H), 6.76 – 6.69 (m, 1H), 4.56 (t, *J* = 8.7 Hz, 2H), 3.78 – 3.69 (m, 1H), 3.19 (t, *J* = 8.6 Hz, 2H), 2.83 – 2.65 (m, 2H), 2.30 – 2.05 (m, 2H).

^{13}C NMR (101 MHz, $CDCl_3$): δ = 158.9, 135.9, 131.8, 129.2, 128.2, 128.0, 127.5, 127.4, 125.1, 120.8, 109.4, 71.3, 38.0, 36.6, 32.6, 29.9.

FT-IR (neat): 2921, 2854, 2246, 1492, 1219, 772 cm^{-1}.

HRMS (APCI) m/z: $[M+H]^+$ Calcd. for $C_{18}H_{18}NO$ 264.1383; Found 264.1378.

2-Phenylhexanedinitrile (14)

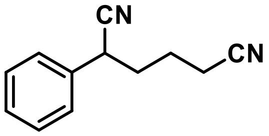

Synthesized following general method A using 2,4,6-collidine (1.0 equiv) instead of BTMG (0.25 equiv) and phen instead of bathophen. 23.5 mg isolated (64%).

Colorless oil.

R_f = 0.4 (petroleum ether/EtOAc 5:1)

^{1}H NMR (400 MHz, $CDCl_3$): δ = 7.46 – 7.30 (m, 5H), 3.87 (t, J = 7.1 Hz, 1H), 2.48 – 2.34 (m, 2H), 2.14 – 2.01 (m, 2H), 1.93 – 1.74 (m, 2H).

^{13}C NMR (101 MHz, $CDCl_3$): δ = 134.8, 129.5, 128.6, 127.3, 120.1, 118.8, 36.9, 34.6, 22.9, 16.9.

Spectral data for this compound were consistent with those in the literature.[2]

Ethyl 3-cyano-3-phenylpropanoate (15)

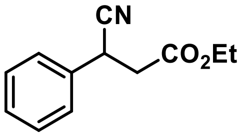

Synthesized following general method A using 2,4,6-collidine (1.0 equiv) instead of BTMG (0.25 equiv). 24.4 mg isolated (60%).

Colorless oil.

R_f = 0.4 (petroleum ether/EtOAc 5:1)

^{1}H NMR (400 MHz, $CDCl_3$): δ = 7.44 – 7.30 (m, 5H), 4.30 (t, J = 7.4 Hz, 1H), 4.23 – 4.12 (m, 2H), 3.06 – 2.95 (m, 1H), 2.89 – 2.78 (m, 1H), 1.24 (t, J = 7.1 Hz, 3H).

^{13}**C NMR (101 MHz, CDCl$_3$):** δ = 169.3, 134.6, 129.4, 128.7, 127.5, 120.1, 61.6, 40.2, 33.3, 14.2.

Spectral data for this compound were consistent with those in the literature.[1]

2-Phenylpent-4-enenitrile (16)

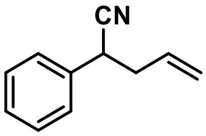

Synthesized following general method A using 2,4,6-collidine (1.0 equiv) instead of BTMG (0.25 equiv). 24.5 mg isolated (78%).

Colorless oil.

R$_f$ = 0.6 (petroleum ether/EtOAc 10:1)

^{1}**H NMR (400 MHz, CDCl$_3$):** δ = 7.43 – 7.29 (m, 5H), 5.88 – 5.72 (m, 1H), 5.24 – 5.13 (m, 2H), 3.85 (t, J = 7.2 Hz, 1H), 2.72 – 2.53 (m, 2H).

^{13}**C NMR (101 MHz, CDCl$_3$):** δ = 135.3, 132.7, 129.2, 128.3, 127.5, 120.4, 119.5, 40.0, 37.7.

Spectral data for this compound were consistent with those in the literature.[6]

1-(4-Bromophenyl)cyclopropane-1-carbonitrile (17)

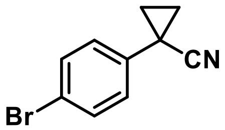

Synthesized following general method A. 22 mg isolated (50%).

Colorless oil.

$\mathbf{R_f}$ = 0.4 (petroleum ether/EtOAc 10:1)

^{1}H NMR (400 MHz, CDCl$_3$): δ = 7.47 (d, J = 8.6 Hz, 2H), 7.16 (d, J = 8.6 Hz, 2H), 1.77 – 1.71 (m, 2H), 1.41- 1.35 (m, 2H).

^{13}C NMR (101 MHz, CDCl$_3$): δ = 135.3, 132.2, 127.6, 122.2, 121.8, 18.4, 13.7.

Spectral data for this compound were consistent with those in the literature.[7]

1-(4-Chlorophenyl)cyclobutane-1-carbonitrile (18)

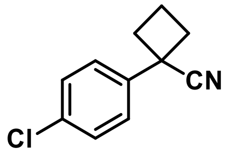

Synthesized following general method A using 2,4,6-collidine (1.0 equiv) instead of BTMG (0.25 equiv) and phen instead of bathophen. 21.4 mg isolated (56%).

Colorless oil.

$\mathbf{R_f}$ = 0.6 (petroleum ether/EtOAc 10:1)

^{1}H NMR (400 MHz, CDCl$_3$): δ = 7.43 – 7.31 (m, 4H), 2.89 – 2.77 (m, 2H), 2.66 – 2.52 (m, 2H), 2.51 – 2.35 (m, 1H), 2.15 – 2.00 (m, 1H).

^{13}C NMR (101 MHz, CDCl$_3$): δ = 138.5, 134.0, 129.3, 127.2, 124.1, 39.9, 34.8, 17.2.

Spectral data for this compound were consistent with those in the literature.[8]

1-Tosylpiperidine-4-carbonitrile (19)

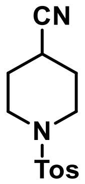

Synthesized following general method A using MeCN/DMF (15:1 v/v, 4 mL) as the solvent.

28.6 mg isolated (54%).

White solid.

R$_f$ = 0.2 (petroleum ether/EtOAc 5:1)

^{1}H NMR (400 MHz, CDCl$_3$): δ = 7.63 (d, J = 8.1 Hz, 2H), 7.34 (d, J = 7.9 Hz, 2H), 3.20 – 3.03 (m, 4H), 2.77 – 2.68 (m, 1H), 2.45 (s, 3H), 2.06 – 1.86 (m, 4H).

^{13}C NMR (101 MHz, CDCl$_3$): δ = 144.2, 132.9, 130.0, 127.7, 120.5, 43.9, 28.1, 25.5, 21.7.

Spectral data for this compound were consistent with those in the literature.[9]

2-Benzylbutanenitrile (20)

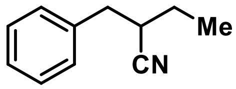

Synthesized following general method A using MeCN/DMF (15:1 v/v, 4 mL) as the solvent.

19 mg isolated (60%).

Colorless oil.

R$_f$ = 0.6 (petroleum ether/EtOAc 10:1)

^{1}H NMR (400 MHz, CDCl$_3$): δ = 7.37 – 7.30 (m, 2H), 7.30 – 7.20 (m, 3H), 2.98 – 2.81 (m, 2H), 2.77 – 2.66 (m, 1H), 1.74 – 1.58 (m, 2H), 1.11 (t, J = 7.4 Hz, 3H).

^{13}C NMR (101 MHz, CDCl$_3$): δ = 137.3, 129.2, 128.9, 127.4, 121.9, 38.3, 35.7, 25.3, 11.8.

Spectral data for this compound were consistent with those in the literature.[10]

2-Benzyl-3-phenylpropanenitrile (21)

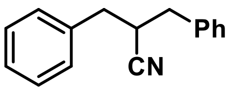

Synthesized following general method A. 24.8 mg isolated (56%).

Colorless oil.

R_f = 0.6 (petroleum ether/EtOAc 10:1)

^{1}H NMR (400 MHz, $CDCl_3$): δ = 7.40 – 7.19 (m, 10H), 3.06 – 2.97 (m, 1H), 2.91 (d, J = 7.0 Hz, 4H).

^{13}C NMR (101 MHz, $CDCl_3$): δ = 136.9, 129.2, 128.9, 127.5, 121.4, 38.1, 36.0.

Spectral data for this compound were consistent with those in the literature.[9]

2-(2-Bromobenzyl)undec-10-enenitrile (22)

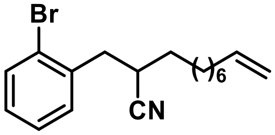

Synthesized following general method A. 34 mg isolated (51%).

Colorless oil.

R_f = 0.6 (petroleum ether/EtOAc 5:1)

^{1}H NMR (400 MHz, $CDCl_3$): δ = 7.54 (d, J = 8.0 Hz, 1H), 7.24 – 7.16 (m, 2H), 7.12 – 7.03 (m, 1H), 5.87 – 5.73 (m, 1H), 5.05 – 4.88 (m, 2H), 3.08 – 2.89 (m, 2H), 2.89 – 2.77 (m, 1H), 2.09 – 1,96 (m, 2H), 1.76 – 1.61 (m, 1H), 1.61 – 1.48 (m, 1H), 1.45 – 1.20 (m, 10H).

^{13}C NMR (101 MHz, CDCl$_3$): δ = 139.3, 138.7, 133.1, 131.4, 128.4, 127.5, 124.8, 114.3, 45.4, 38.4, 33.9, 32.3, 29.5, 29.4, 29.2, 29.0, 27.2.

FT-IR (neat): 2927, 2855, 1706, 749 cm^{-1}.

HRMS (APCI) m/z: $[M+H]^+$ Calcd. for $C_{18}H_{25}BrN$ 334.1165; Found 334.1173.

2-(4-Methoxyphenethyl)pent-4-enenitrile (23)

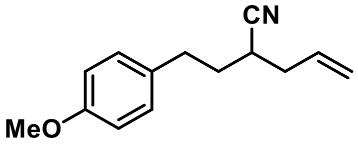

Synthesized following general method A. 21.5 mg isolated (50%).

Colorless oil.

R$_f$ = 0.6 (petroleum ether/EtOAc 5:1)

^{1}H NMR (400 MHz, CDCl$_3$): δ = 7.11 (d, J = 8.5 Hz, 2H), 6.85 (d, J = 8.5 Hz, 2H), 5.90 – 5.65 (m, 1H), 5.27 – 5.07 (m, 2H), 3.79 (s, 3H), 2.91 – 2.77 (m, 1H), 2.75 – 2.61 (m, 1H), 2.60 – 2.51 (m, 1H), 2.45 – 2.27 (m, 2H), 1.99 – 1.75 (m, 2H).

^{13}C NMR (101 MHz, CDCl$_3$): δ = 158.3, 133.1, 132.2, 129.5, 121.7, 119.1, 114.2, 55.4, 36.4, 33.7, 32.4, 30.8.

FT-IR (neat): 2931, 2238, 1513, 1247 cm^{-1}.

HRMS (APCI) m/z: $[M+H]^+$ Calcd. for $C_{14}H_{18}NO$ 216.1383; Found 216.1375.

2-(4-Methoxyphenethyl)pent-4-enenitrile (24)

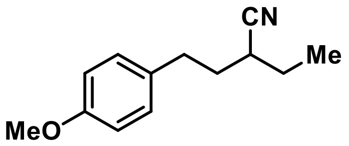

Synthesized following general method A. 20.3 mg isolated (50%).

Colorless oil.

R_f = 0.6 (petroleum ether/EtOAc 5:1)

^{1}H NMR (400 MHz, $CDCl_3$): δ = 7.11 (d, J = 8.4 Hz, 2H), 6.85 (d, J = 8.5 Hz, 2H), 3.79 (s, 3H), 2.88 – 2.77 (m, 1H), 2.73 – 2.61 (m, 1H), 2.49 – 2.36 (m, 1H), 1.98 – 1.85 (m, 1H), 1.85 – 1.74 (m, 1H), 1.67 – 1.57 (m, 3H), 1.07 (t, J = 7.4 Hz, 3H).

^{13}C NMR (101 MHz, $CDCl_3$): δ = 158.3, 132.4, 129.5, 122.1, 114.2, 55.4, 34.0, 32.6, 32.5, 25.7, 11.7.

FT-IR (neat): 2935, 2237, 1513, 1247 cm^{-1}.

HRMS (APCI) m/z: $[M+H]^+$ Calcd. for $C_{13}H_{18}NO$ 204.1383; Found 204.1376.

3,3-Diphenylpropanenitrile (25)

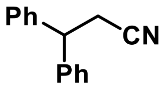

Synthesized following general method A using MeCN/DMF (15:1 v/v, 4 mL) as the solvent. 26.9 mg isolated (65%).

Colorless oil.

R_f = 0.6 (petroleum ether/EtOAc 10:1)

^{1}H NMR (400 MHz, $CDCl_3$): δ = 7.39 – 7.29 (m, 4H), 7.29 – 7.18 (m, 6H), 4.38 (t, J = 7.7 Hz, 1H), 3.03 (d, J = 7.7 Hz, 2H).

^{13}C NMR (101 MHz, $CDCl_3$): δ = 141.4, 129.0, 127.7, 127.5, 118.5, 47.3, 24.4.

Spectral data for this compound were consistent with those in the literature.[11]

4-(4-Methoxyphenyl)butanenitrile (26)

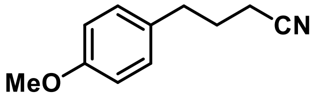

Synthesized following general method A using MeCN/DMF (15:1 v/v, 4 mL) as the solvent. 19.6 mg isolated (56%).

Colorless oil.

R_f = 0.6 (petroleum ether/EtOAc 5:1)

^{1}H NMR (400 MHz, $CDCl_3$): δ = 7.10 (d, J = 8.5 Hz, 2H), 6.85 (d, J = 8.6 Hz, 2H), 3.79 (s, 3H), 2.72 (t, J = 7.4 Hz, 2H), 2.30 (t, J = 7.1 Hz, 2H), 2.03 – 1.86 (m, 2H).

^{13}C NMR (101 MHz, $CDCl_3$): δ = 158.4, 131.9, 129.5, 119.7, 114.2, 55.4, 33.6, 27.3, 16.4.

Spectral data for this compound were consistent with those in the literature.[12]

4-Phenylbutanenitrile (27)

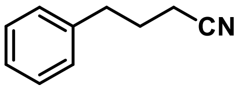

Synthesized following general method A using MeCN/DMF (15:1 v/v, 4 mL) as the solvent and $LiClO_4$ (2.0 equiv) as the electrolyte, 2,4,6-collidine (1.0 equiv) as the base. 14.5 mg isolated (50%).

Colorless oil.

R_f = 0.6 (petroleum ether/EtOAc 10:1)

^{1}H NMR (400 MHz, $CDCl_3$): δ = 7.35 – 7.27 (m, 2H), 7.27 – 7.22 (m, 1H), 7.22 – 7.15 (m, 2H), 2.78 (t, J = 7.4 Hz, 2H), 2.32 (t, J = 7.1 Hz, 2H), 2.05 – 1.89 (m, 2H).

^{13}C NMR (101 MHz, $CDCl_3$): δ = 139.8, 128.8, 128.6, 126.7, 119.6, 34.5, 27.1, 16.5.

Spectral data for this compound were consistent with those in the literature.[9]

5-Oxo-5-phenylpentanenitrile (28)

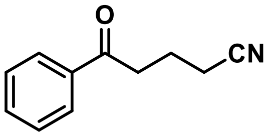

Synthesized following general method A using MeCN/DMF (15:1 v/v, 4 mL) as the solvent and $LiClO_4$ (2.0 equiv) as the electrolyte, 2,4,6-collidine (1.0 equiv) as the base. 9.3 mg isolated (27%).

Colorless oil.

R_f = 0.3 (petroleum ether/EtOAc 5:1)

^{1}H NMR (400 MHz, $CDCl_3$): δ = 7.97 (d, J = 7.5 Hz, 2H), 7.64 – 7.54 (m, 1H), 7.54 – 7.42 (m, 2H), 3.19 (t, J = 6.8 Hz, 2H), 2.53 (t, J = 6.9 Hz, 2H), 2.18 – 2.05 (m, 2H).

^{13}C NMR (101 MHz, $CDCl_3$): δ = 198.3, 136.6, 133.6, 128.9, 128.1, 36.5, 19.87, 16.8.

Spectral data for this compound were consistent with those in the literature.[13]

3-(Benzyloxy)-2,2-dimethylpropanenitrile (29)

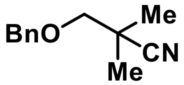

Synthesized following general method A. 16.6 mg isolated (44%).

Colorless oil.

R_f = 0.6 (petroleum ether/EtOAc 5:1)

^{1}H NMR (400 MHz, CDCl$_3$): δ = 7.42 – 7.26 (m, 5H), 4.62 (s, 2H), 3.39 (s, 2H), 1.36 (s, 6H).

^{13}C NMR (101 MHz, CDCl$_3$): δ = 137.7, 128.6, 128.0, 127.7, 124.2, 75.8, 73.6, 33.7, 23.8.

Spectral data for this compound were consistent with those in the literature.[14]

5-(2,5-Dimethylphenoxy)-2,2-dimethylpentanenitrile (30)

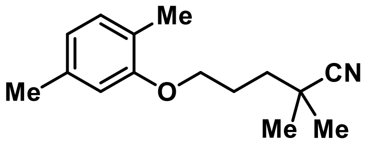

Synthesized following general method A. 20.3 mg isolated (44%).

Colorless oil.

R$_f$ = 0.6 (petroleum ether/EtOAc 10:1)

^{1}H NMR (500 MHz, CDCl$_3$): δ = 7.00 (d, J = 7.5 Hz, 1H), 6.67 (d, J = 7.5 Hz, 1H), 6.62 (s, 1H), 3.99 (t, J = 6.0 Hz, 2H), 2.31 (s, 3H), 2.17 (s, 3H), 2.03 – 1.95 (m, 2H), 1.79 – 1.71 (m, 2H), 1.39 (s, 6H).

^{13}C NMR (126 MHz, CDCl$_3$): δ = 157.0, 136.7, 130.6, 125.0, 123.8, 121.2, 112.2, 67.4, 38.1, 32.4, 26.9, 25.8, 21.5, 15.9.

FT-IR (neat): 2925, 2234, 1265, 1130 cm^{-1}.

HRMS (APCI) m/z: [M+H]$^+$ Calcd. for $C_{15}H_{22}NO$ 232.1696; Found 232.1697.

2-Phenylhex-4-enenitrile (32)

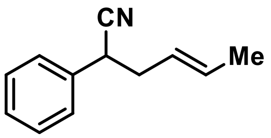

Synthesized following general method A. 21.2 mg isolated (62%), E/Z = 3.2 : 1.

Colorless oil.

R_f = 0.6 (petroleum ether/EtOAc 10:1)

^{1}H NMR (400 MHz, $CDCl_3$, mixture of geometric isomers): δ = 7.47 – 7.29 (m, 5H), 5.75 – 5.51 (m, 1H), 5.53 – 5.35 (m, 1H), 3.87 – 3.71 (m, 1H), 2.80 – 2.42 (m, 2H), 1.68 (d, J = 6.3 Hz, 2H), 1.63 – 1.49 (m, 1H).

^{13}C NMR (101 MHz, $CDCl_3$, mixture of geometric isomers): δ = 135.6, 135.6, 130.4, 129.1, 128.8, 128.2, 128.2, 127.5, 127.5, 125.3, 124.3, 120.7, 39.1, 38.3, 37.6, 33.3, 18.0, 13.0.

FT-IR (neat): 2994, 2919, 2853, 2360, 2240, 1769, 1244 cm^{-1}.

Spectral data for this compound were consistent with those in the literature.[15]

5. References

1. Wang, D.; Zhu, N.; Chen, P.; Lin, Z.; Liu, G., Enantioselective Decarboxylative Cyanation Employing Cooperative Photoredox Catalysis and Copper Catalysis. *J. Am. Chem. Soc.* **2017**, *139*, 15632-15635.

2. Frye, N. L.; Bhunia, A.; Studer, A., Nickel-Catalyzed Markovnikov Transfer Hydrocyanation in the Absence of Lewis Acid. *Org. Lett.* **2020**, *22*, 4456-4460.

3. Kim, K.; Lee, S.; Hong, S. Direct C(sp^3)-H Cyanation Enabled by a Highly Active Decatungstate Photocatalyst. *Org. Lett.* **2021**, *23*, 5501– 5505.

4. Tan, E. S.; Miyakawa, M.; Bunzow, J. R.; Grandy, D. K.; Scanlan, T. S., Exploring the Structure−Activity Relationship of the Ethylamine Portion of 3-Iodothyronamine for Rat and Mouse Trace Amine-Associated Receptor 1. *J. Med. Chem.* **2007**, *50*, 2787–2798.

5. Murata, M.; Oyama, T.; Watanabe, S.; Masuda, Y., Palladium-Catalyzed Borylation of Aryl Halides or Triflates with Dialkoxyborane: A Novel and Facile Synthetic Route to Arylboronates. *J. Org. Chem.* **2000**, *65*, 164–168.

6. Chen, Y.; Xu, L.; Jiang, Y.; Ma, D., Assembly of *α*-(Hetero)aryl Nitriles via Copper-Catalyzed Coupling Reactions with (Hetero)aryl Chlorides and Bromides. *Angew. Chem. Int. Ed.* **2021**, *60*, 7082-7086.

7. Kramm, F.; Ullwer, F.; Klinnert, B.; Zheng, M.; Plietker, B., Iron-Catalyzed Cycloisomerization and C−C Bond Activation to Access Non-canonical Tricyclic Cyclobutanes. *Angew. Chem. Int. Ed.* **2022**, *61*, e2022051.

8. McCabe Dunn, J. M.; Kuethe, J. T.; Orr, R. K.; Tudge, M.; Campeau, L. C., Development of a Palladium-Catalyzed *α*-Arylation of Cyclopropyl Nitriles. *Org. Lett.* **2014**, *16*, 6314–6317.

9. Xia, A.; Lv, P.; Xie, X.; Liu, Y., Nickel-Catalyzed Cyanation of Unactivated Alkyl Sulfonates with $Zn(CN)_2$. *Org. Lett.* **2020**, *22*, 7842–7847.

10. Früh, N.; Togni, A., Vanadium-Catalyzed Solvent-Free Synthesis of Quaternary *α*-Trifluoromethyl Nitriles by Electrophilic Trifluoromethylation. *Angew. Chem. Int. Ed.* **2014**, *53*, 10813– 10816.

11. Bhunia, A.; Bergander, K.; Studer, A., Cooperative Palladium/Lewis Acid-Catalyzed Transfer Hydrocyanation of Alkenes and Alkynes Using 1-Methylcyclohexa-2,5-diene-1-carbonitrile. *J. Am. Chem. Soc.* **2018**, *140*, 16353–16359.

12. Motsch, B. J.; Wengryniu, S. E., Site-Selective Synthesis of *N*-Benzyl 2,4,6-Collidinium Salts by Electrooxidative C–H Functionalization. *Org. Lett.* **2022**, *24*, 6060–6065.

13. Zhu, Y.; Jiang, C.; Li, H.; Liu, P.; Sun, P., Alkenes via C═C Bond Cleavage or Oxygenation and Azidation of Open-Chain Alkenes. *J. Org. Chem.* **2022**, *87*, 11031–11041.

14. Hsieh, J.; Chu, Y.; Muralirajanb, K.; Cheng, C., A simple route to 1,4-addition reactions by Co-catalyzed reductive coupling of organic tosylates and triflates with activated alkenes. *Chem. Commun.*, **2017**, *53*, 11584-11587.

15. Yang, K.; Wang, Y.; Luo, S.; Fu, N., Electrophotochemical Metal‐Catalyzed Enantioselective Decarboxylative Cyanation. *Chem. Eur. J.* **2023**, *29*, e202203962.

6. NMR Spectra

^{1}H NMR of compound 2 ($CDCl_3$, 400 MHz)

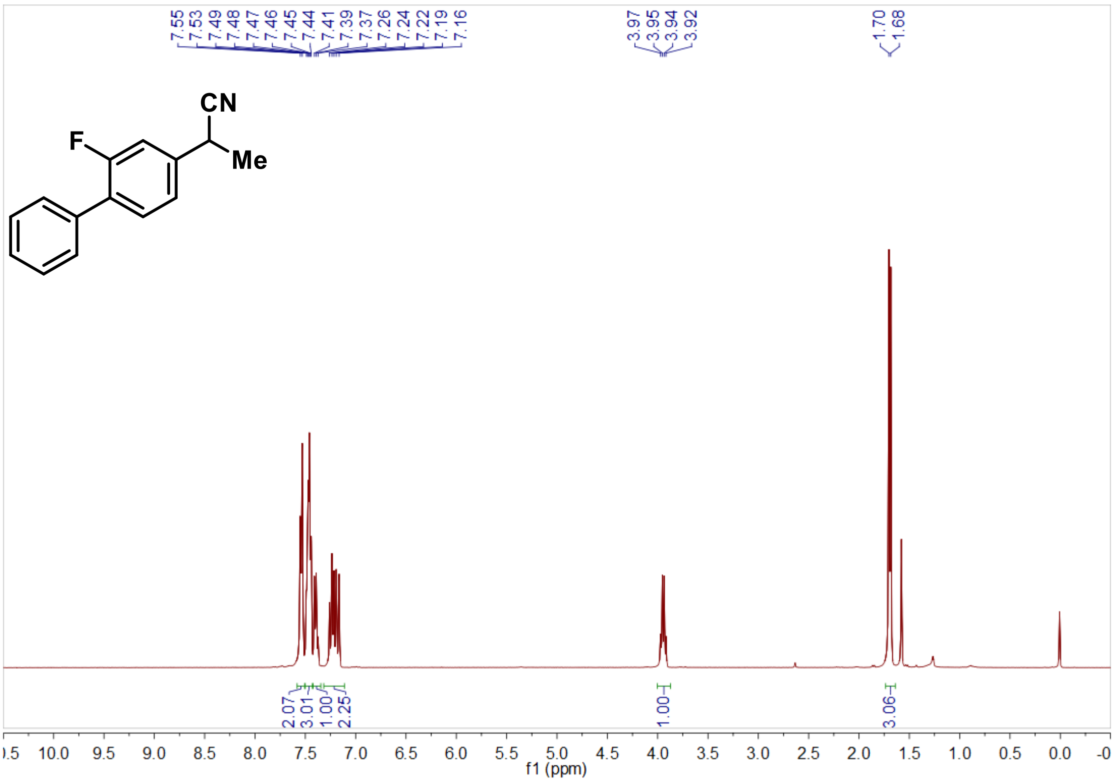

^{13}C NMR of compound 2 ($CDCl_3$, 101 MHz)

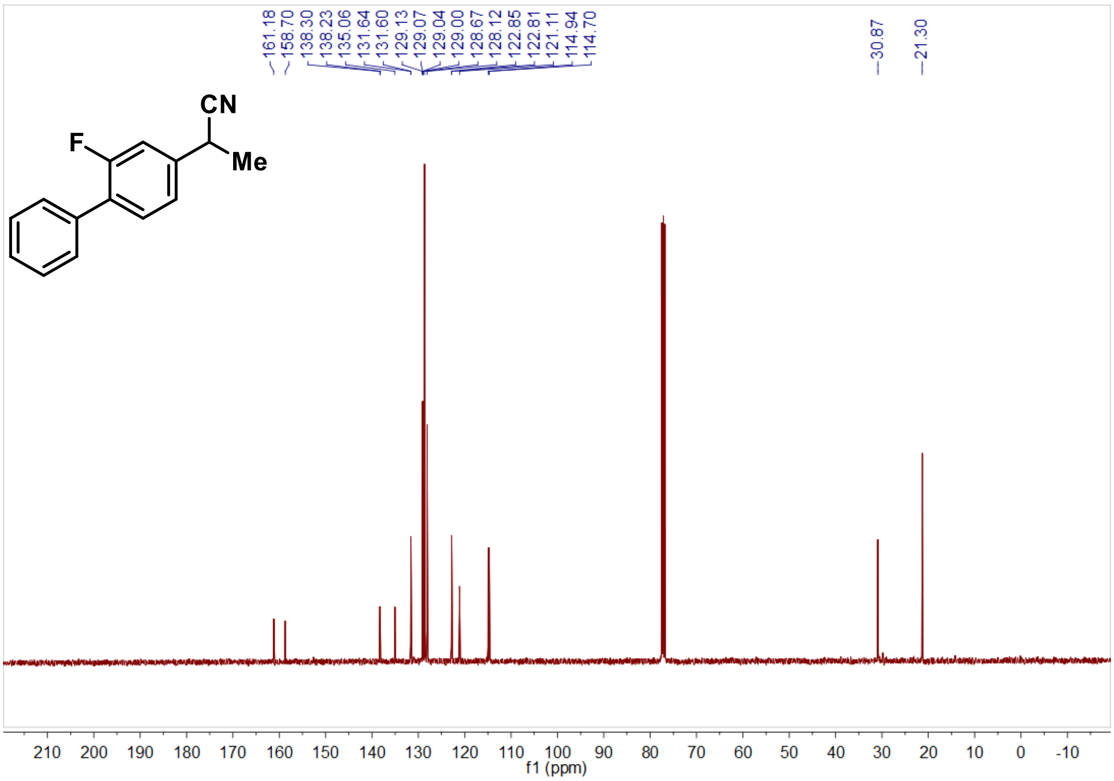

[9]F NMR of compound 2 ($CDCl_3$, 377 MHz)

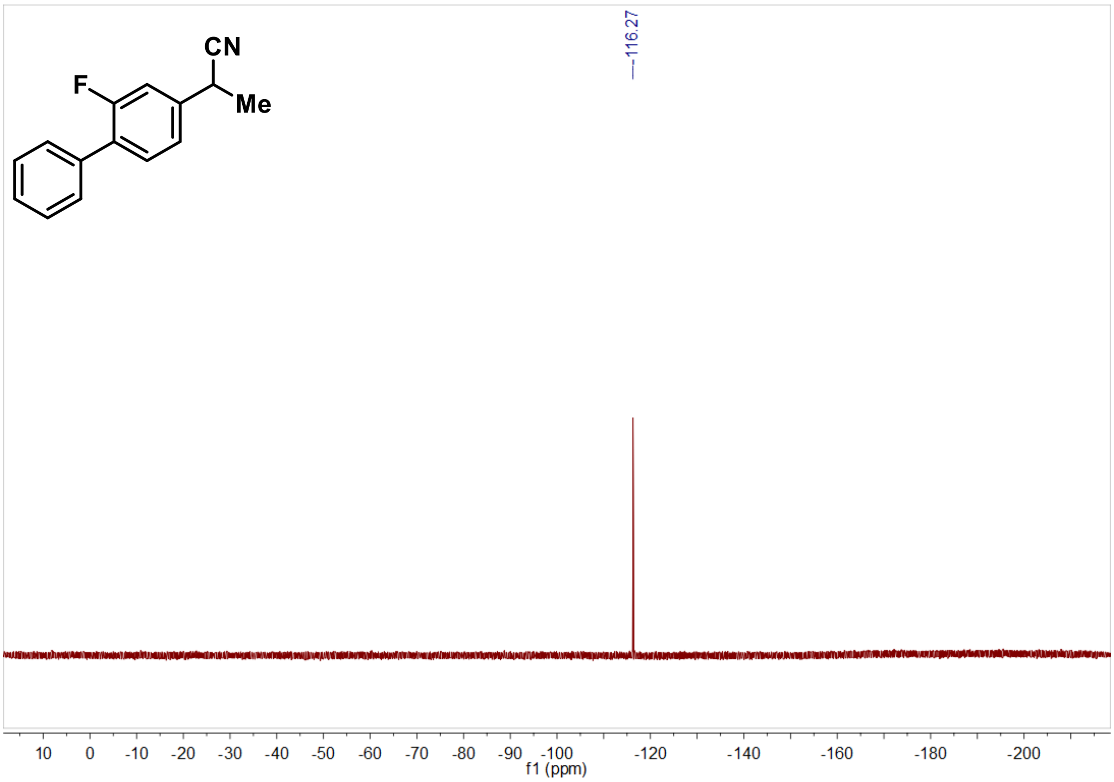

^{1}H NMR of compound 3 ($CDCl_3$, 400 MHz)

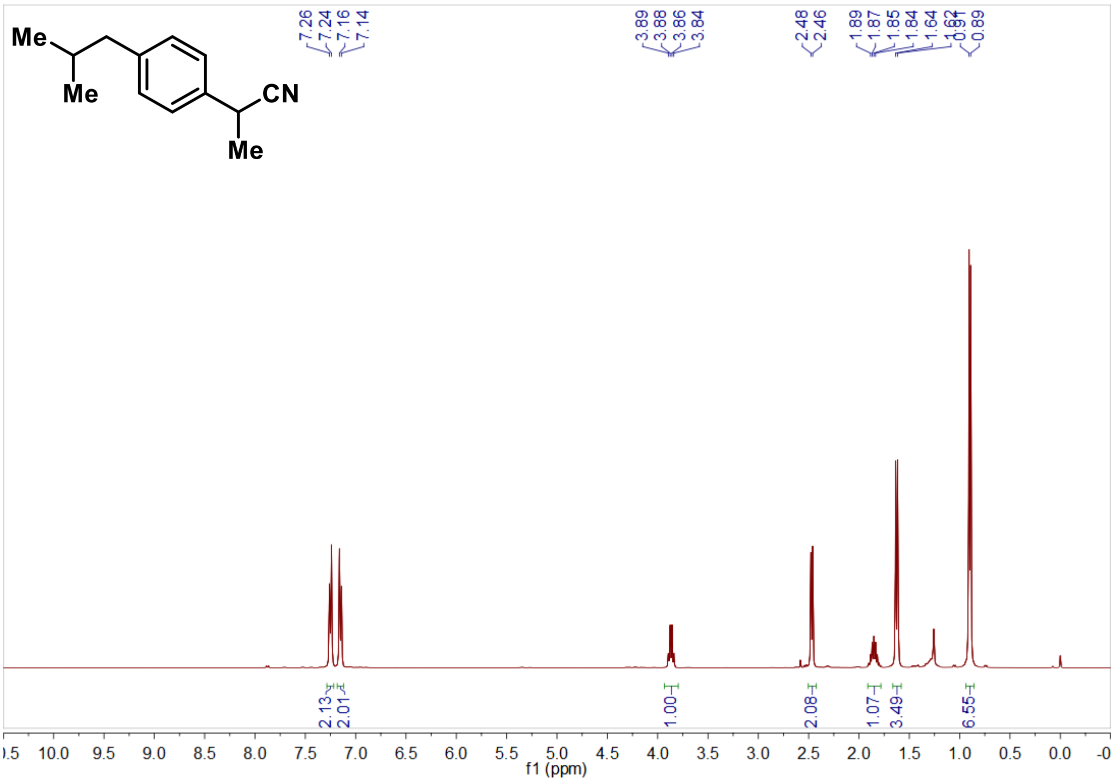

^{13}C NMR of compound 3 ($CDCl_3$, 101 MHz

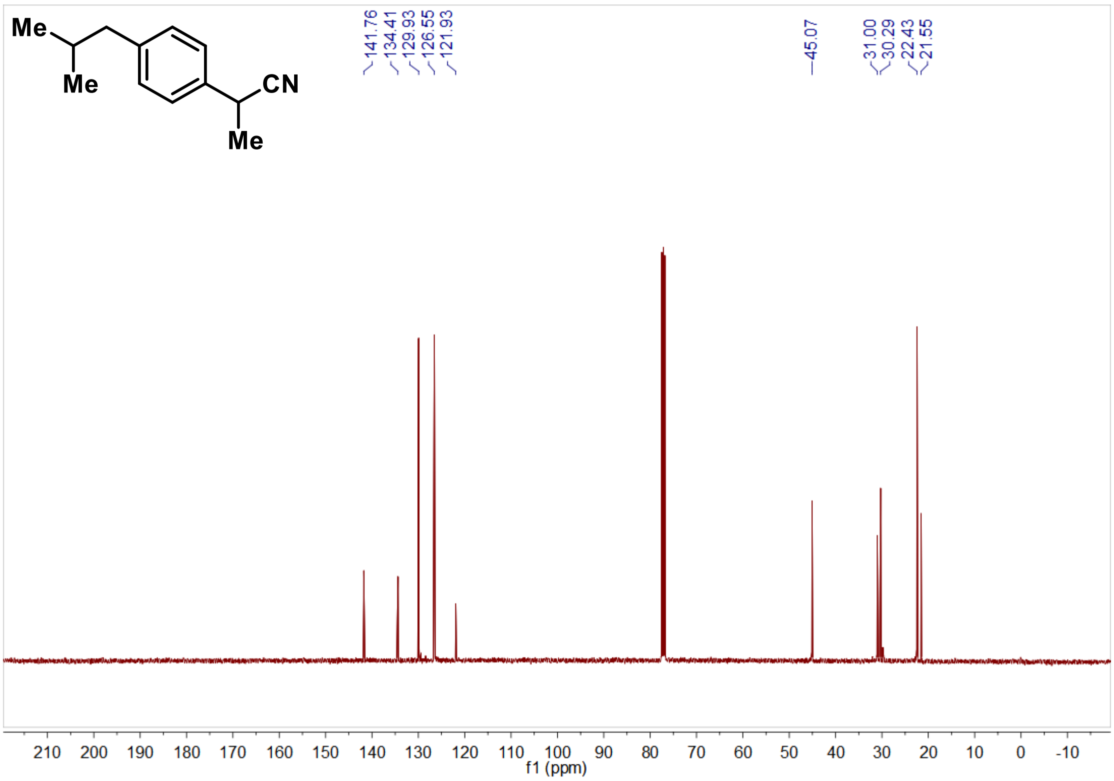

^{1}H NMR of compound 4 ($CDCl_3$, 400 MHz)

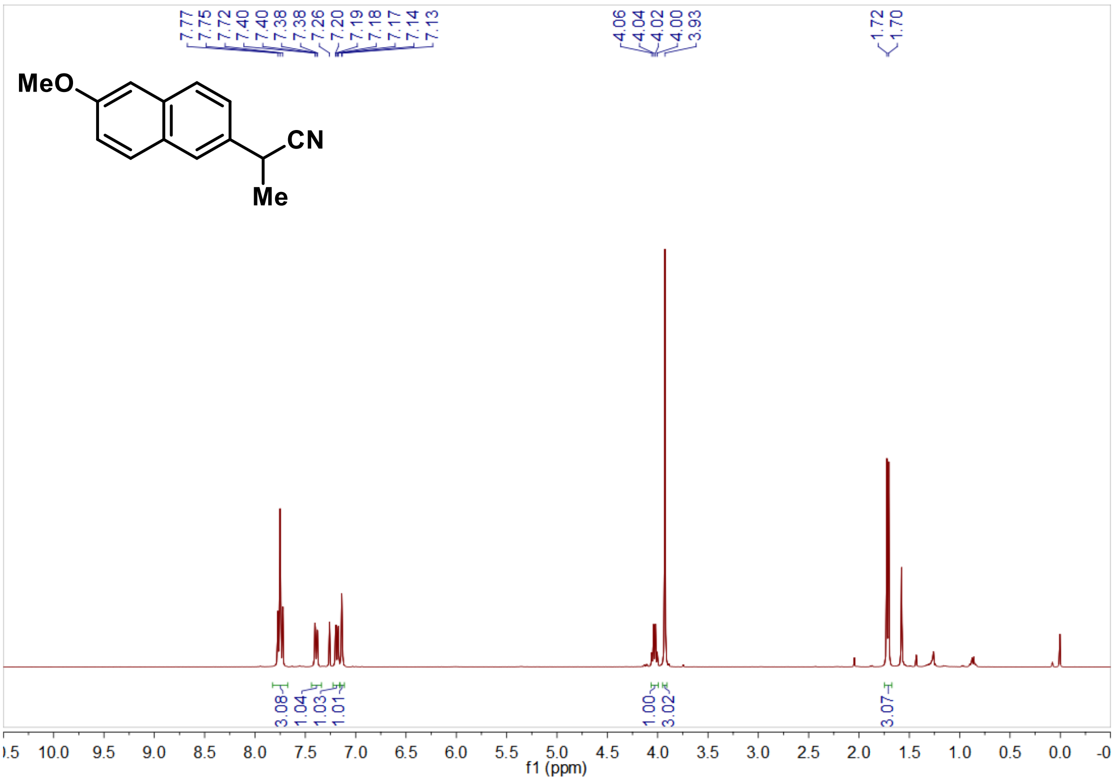

^{13}C NMR of compound 4 ($CDCl_3$, 101 MHz)

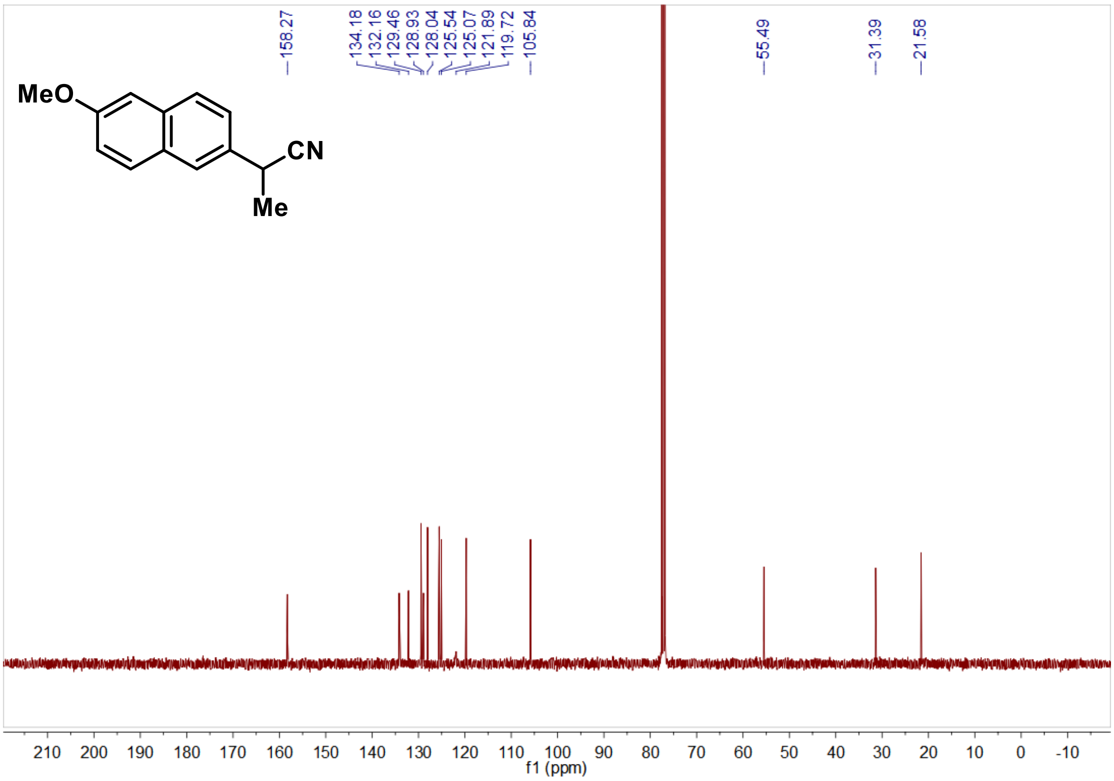

^{1}H NMR of compound 5 ($CDCl_3$, 500 MHz)

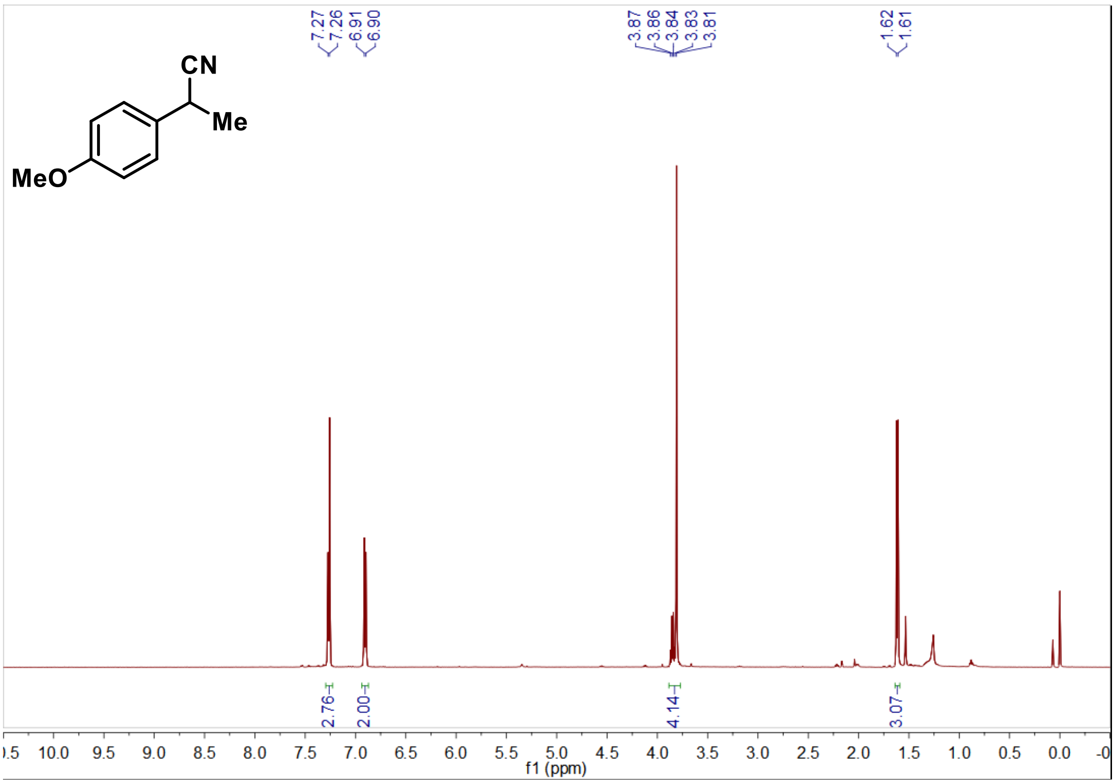

^{13}C NMR of compound 5 ($CDCl_3$, 126 MHz)

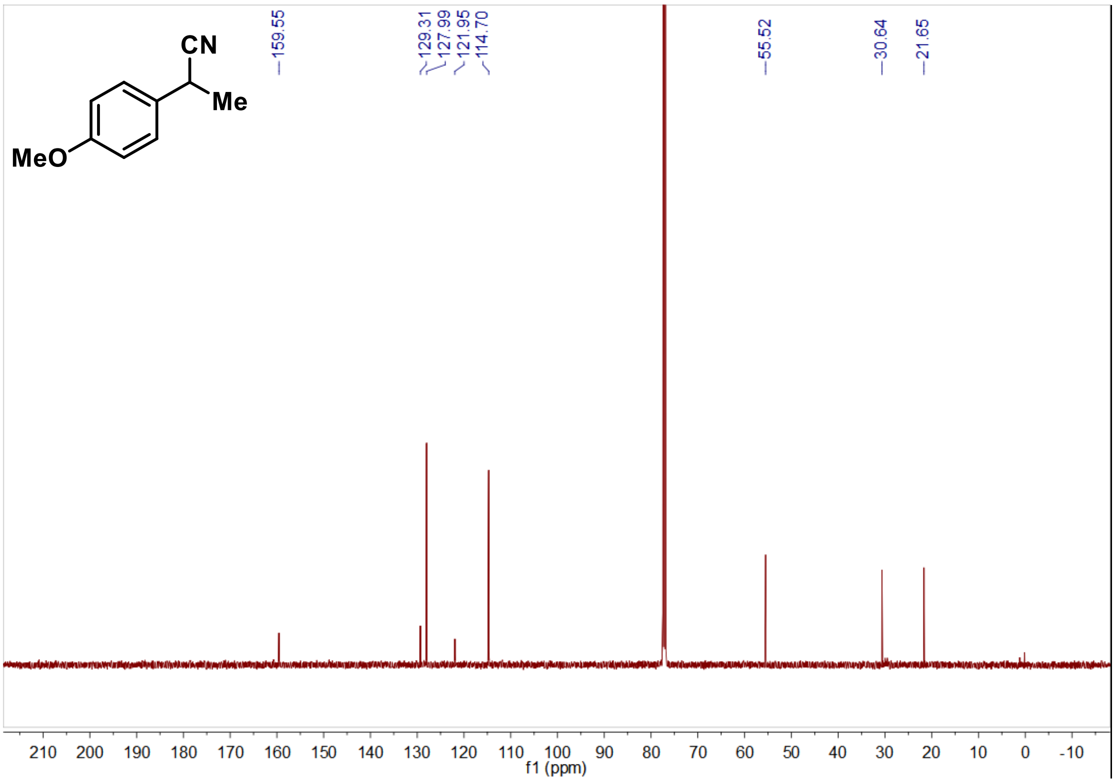

^{1}H NMR of compound 6 ($CDCl_3$, 400 MHz)

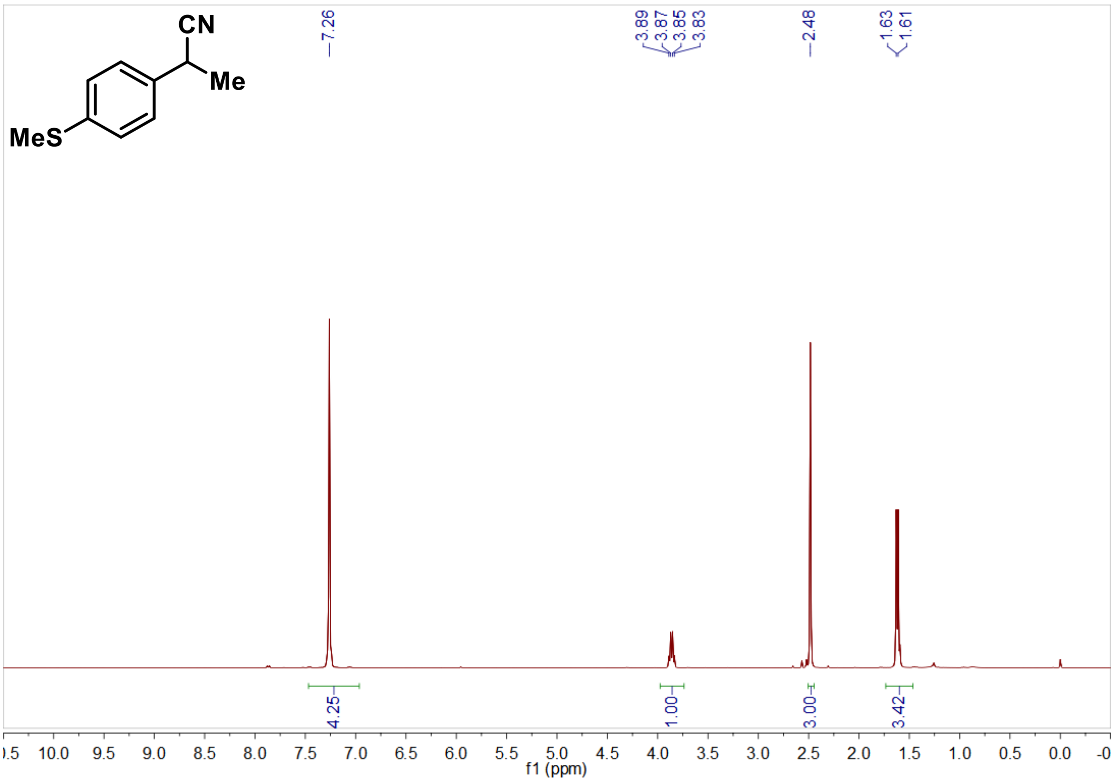

^{13}C NMR of compound 6 ($CDCl_3$, 101 MHz)

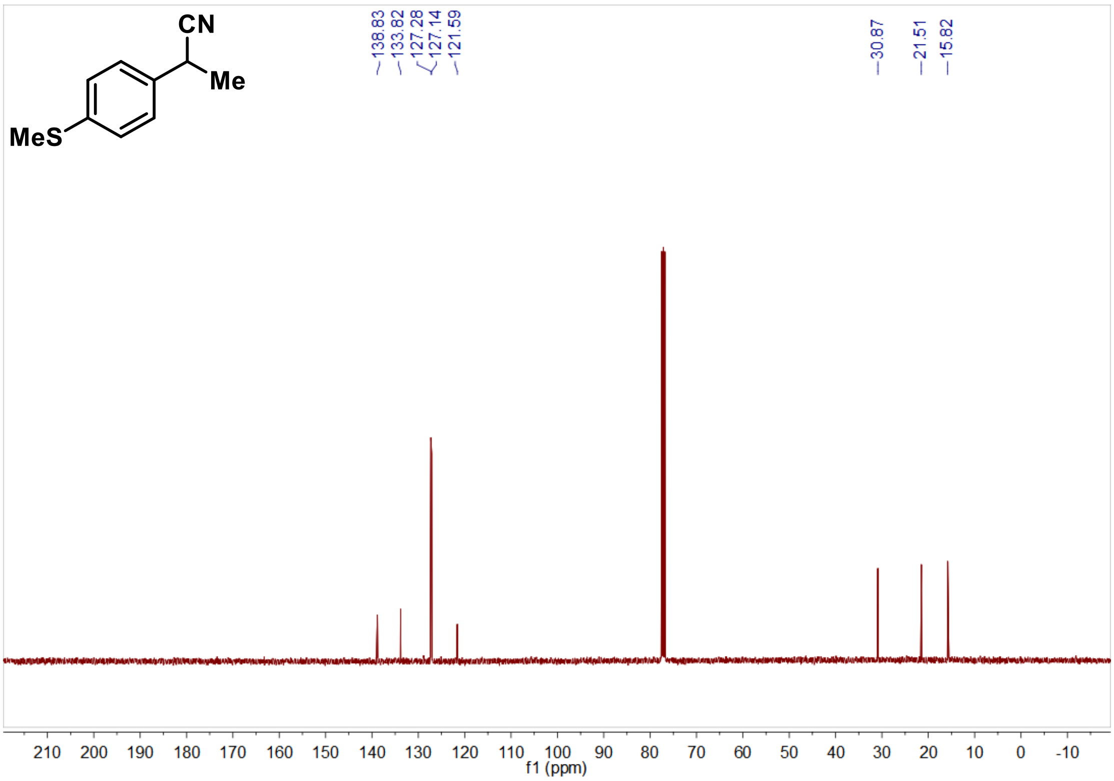

^{1}H NMR of compound 7 ($CDCl_3$, 400 MHz)

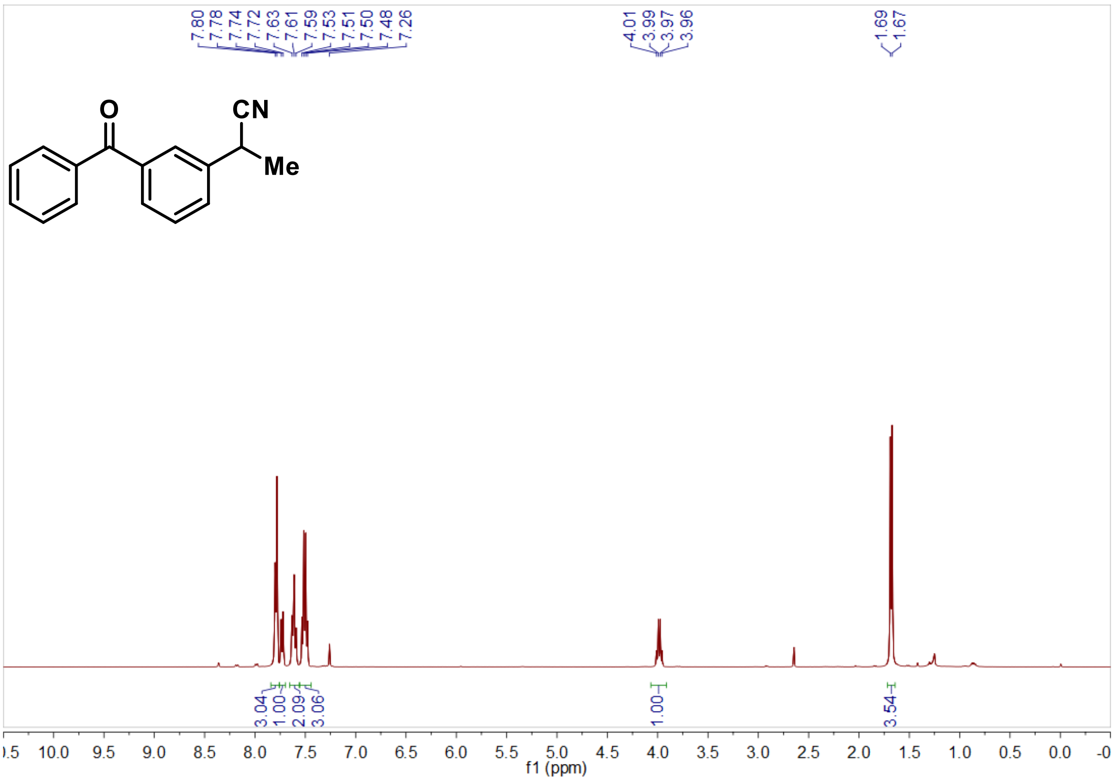

^{13}C NMR of compound 7 ($CDCl_3$, 101 MHz)

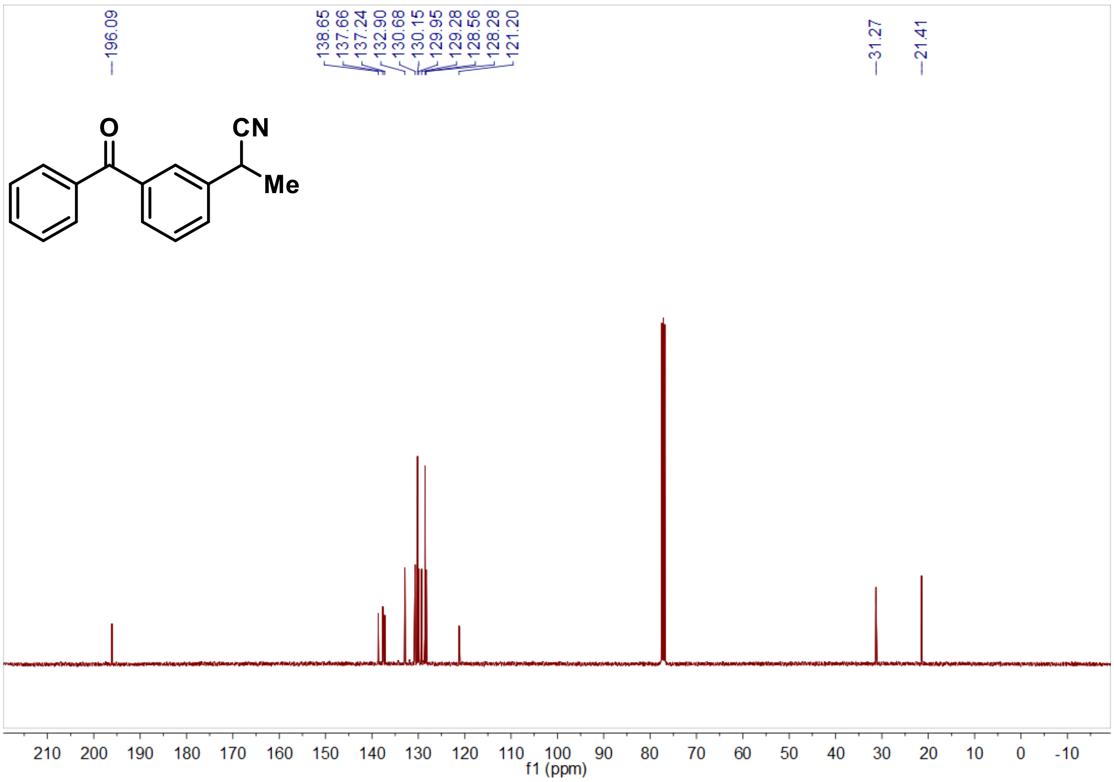

^{1}H NMR of compound 8 ($CDCl_3$, 400 MHz)

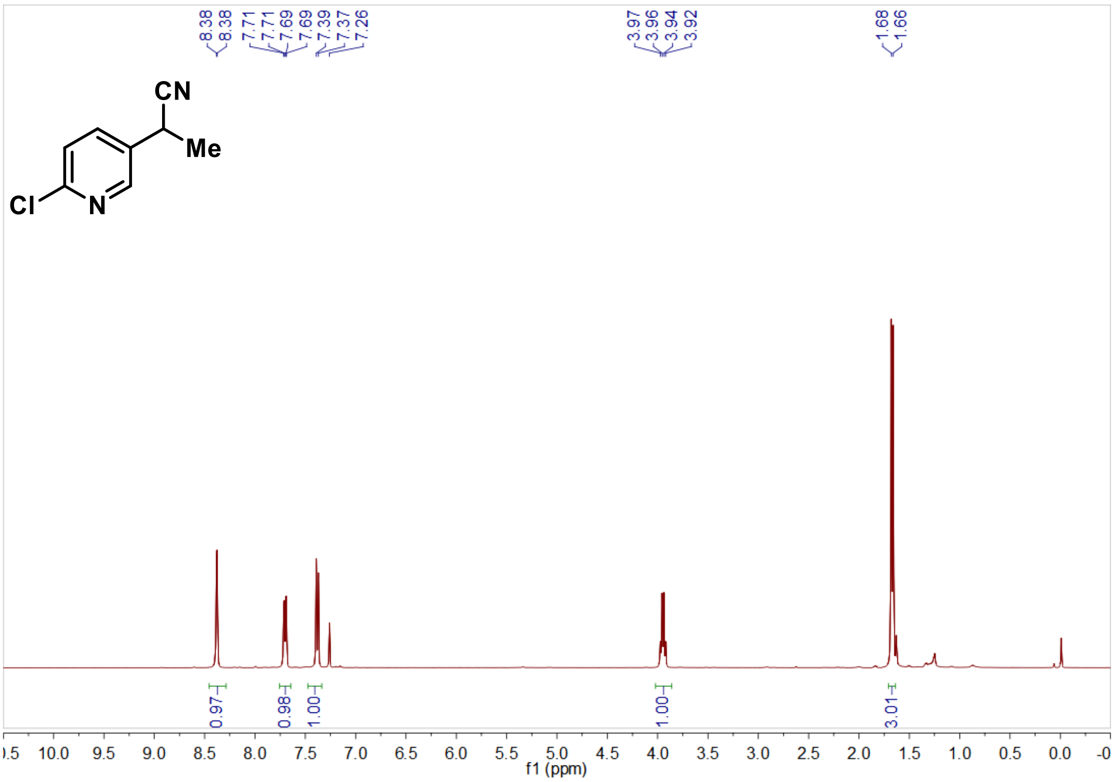

^{13}C NMR of compound 8 ($CDCl_3$, 101 MHz)

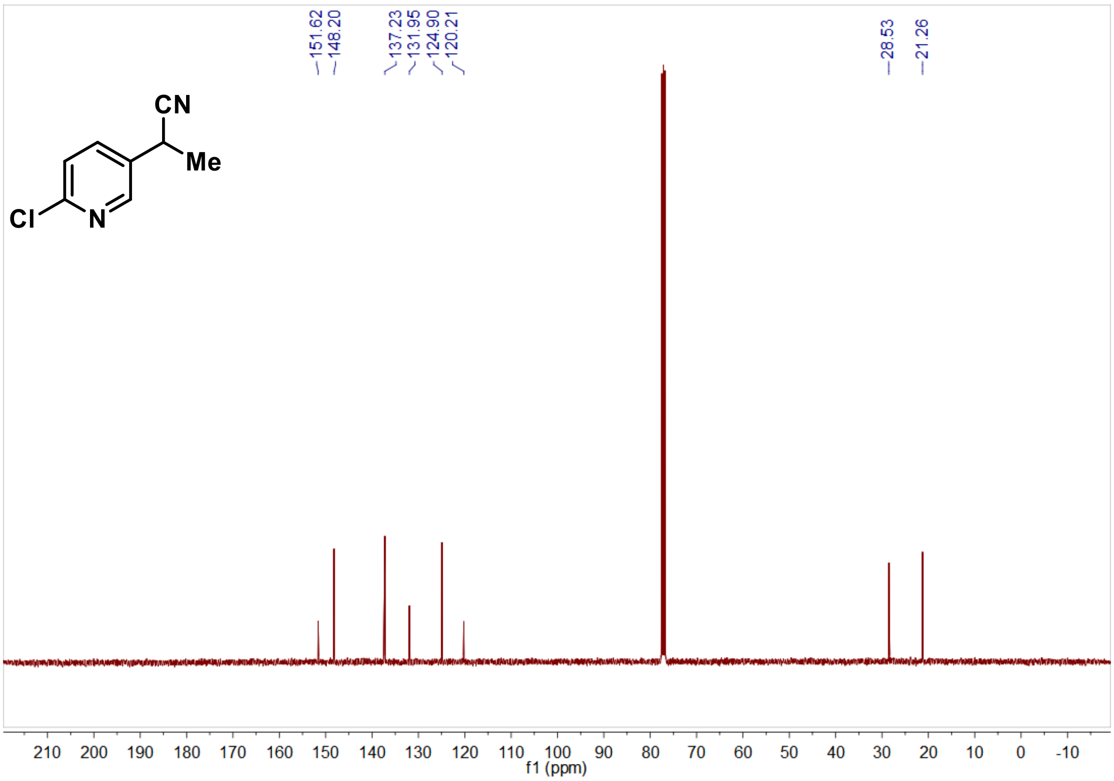

^{1}H NMR of compound 9 ($CDCl_3$, 400 MHz)

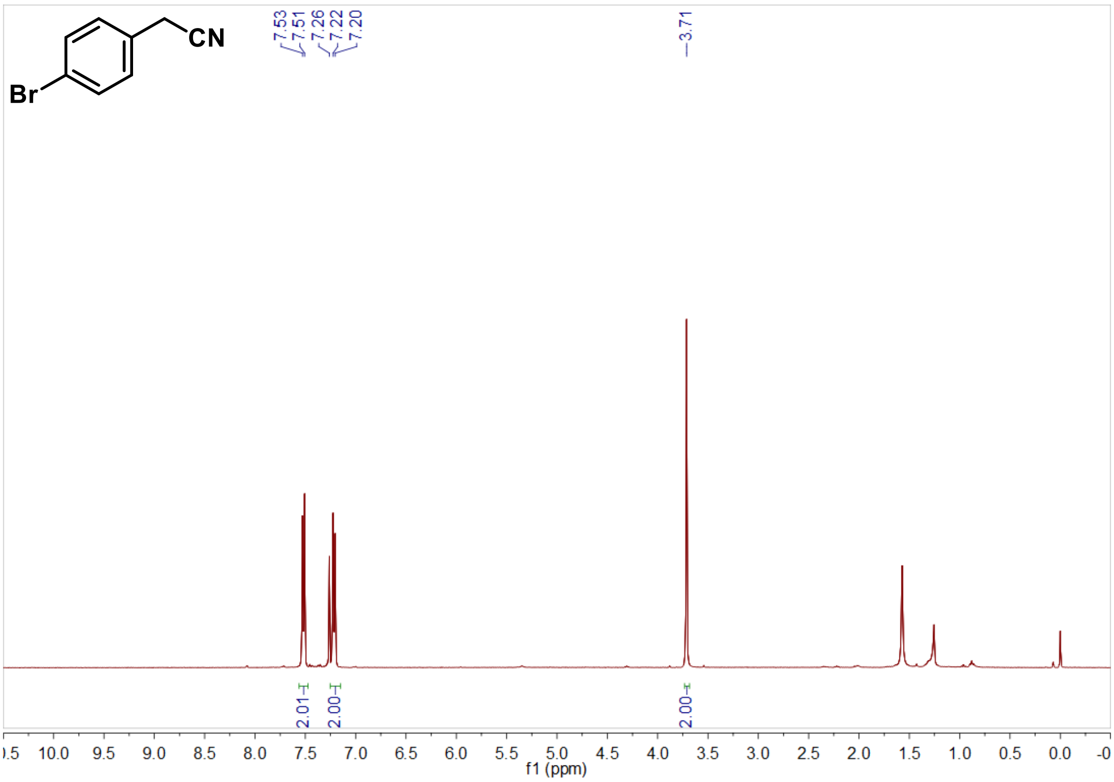

^{13}C NMR of compound 9 ($CDCl_3$, 101 MHz)

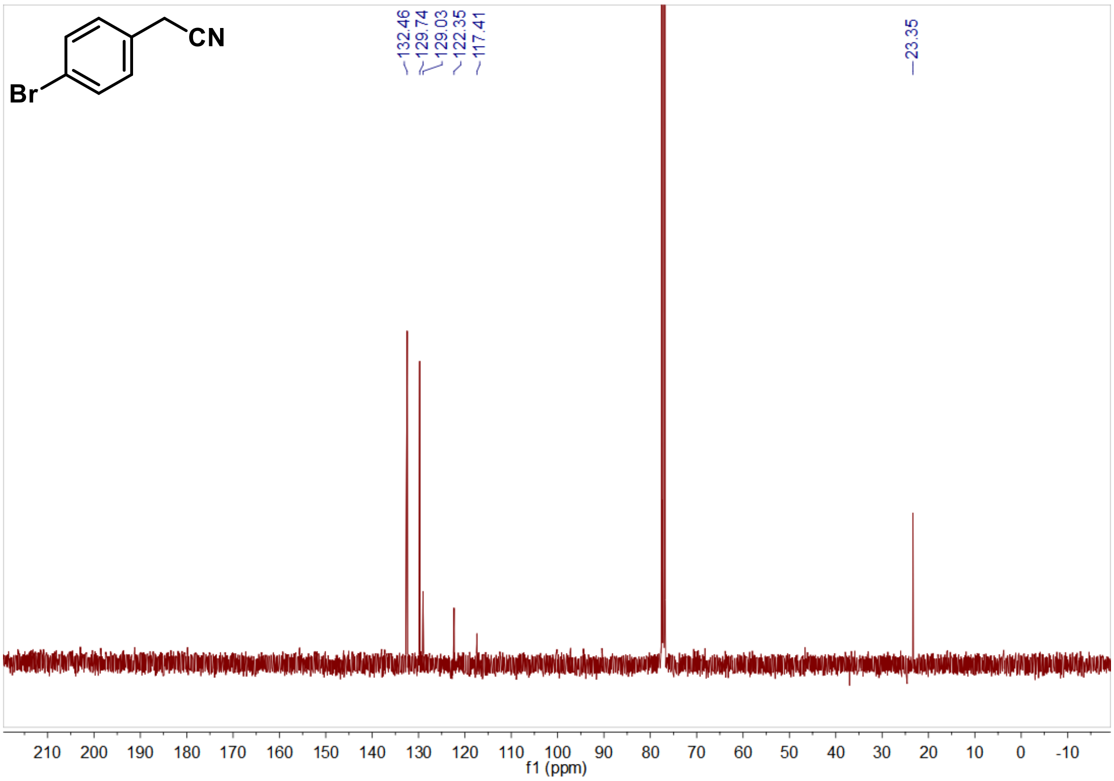

^{1}H NMR of compound 10 ($CDCl_3$, 500 MHz)

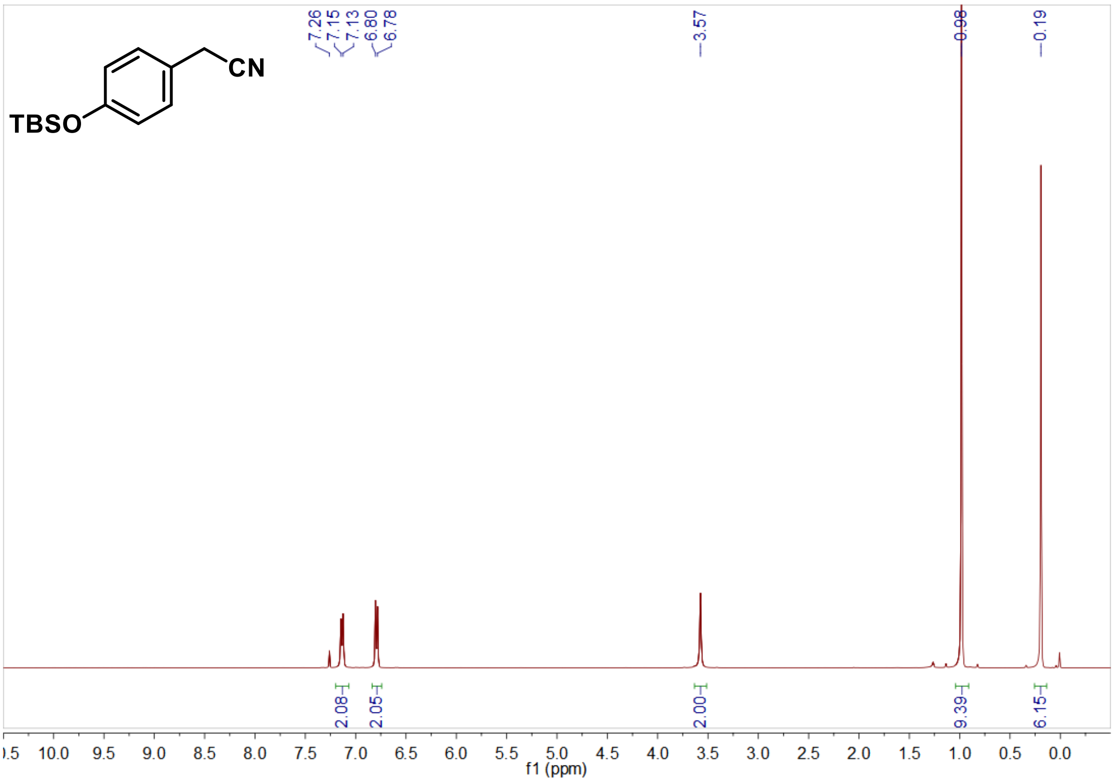

^{13}C NMR of compound 10 ($CDCl_3$, 126 MHz)

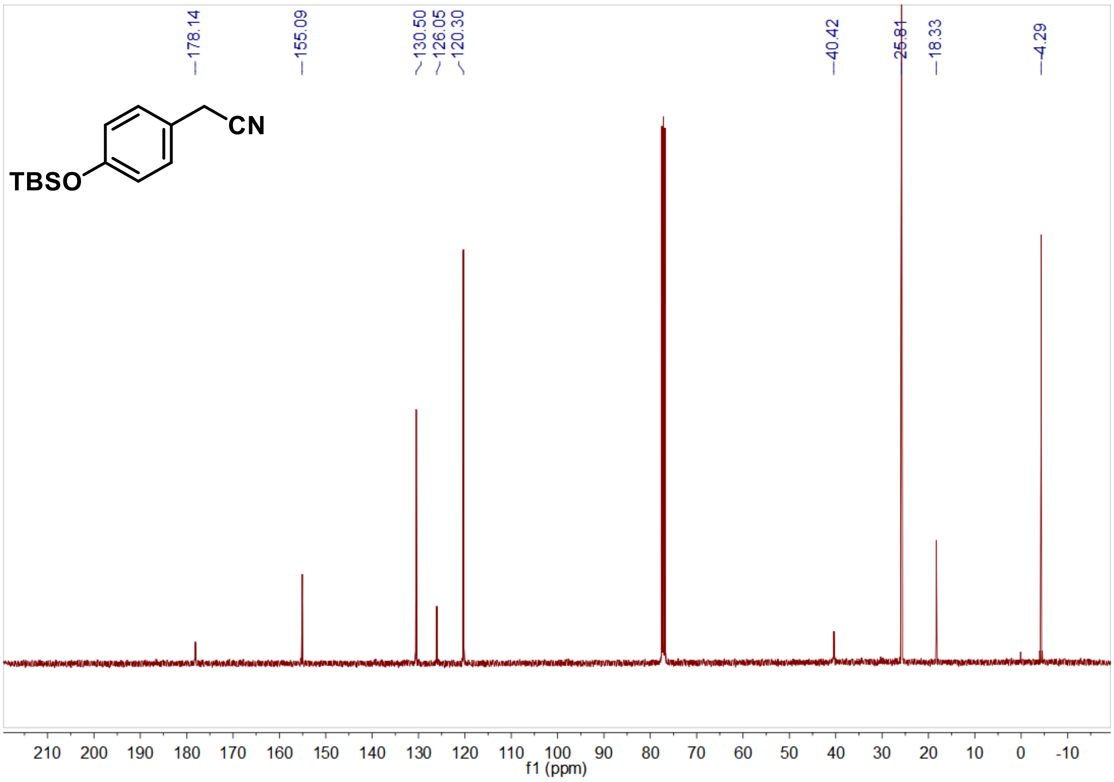

^{1}H NMR of compound 11 ($CDCl_3$, 500 MHz)

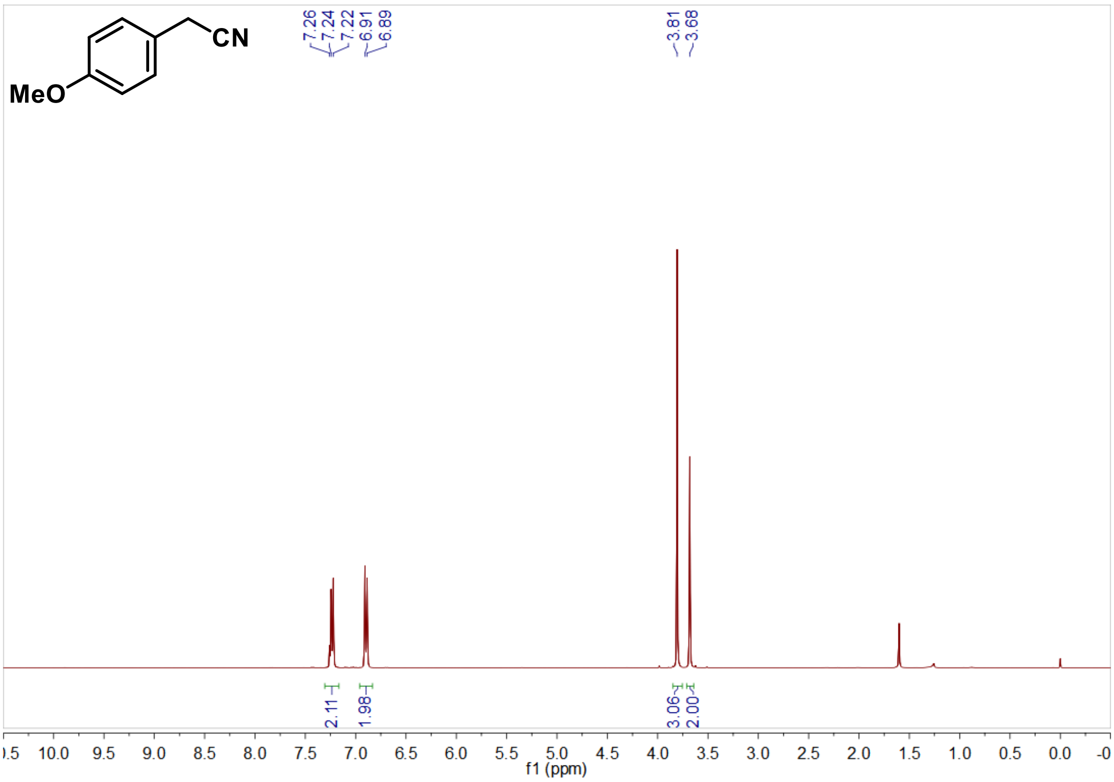

^{13}C NMR of compound 11 ($CDCl_3$, 126 MHz)

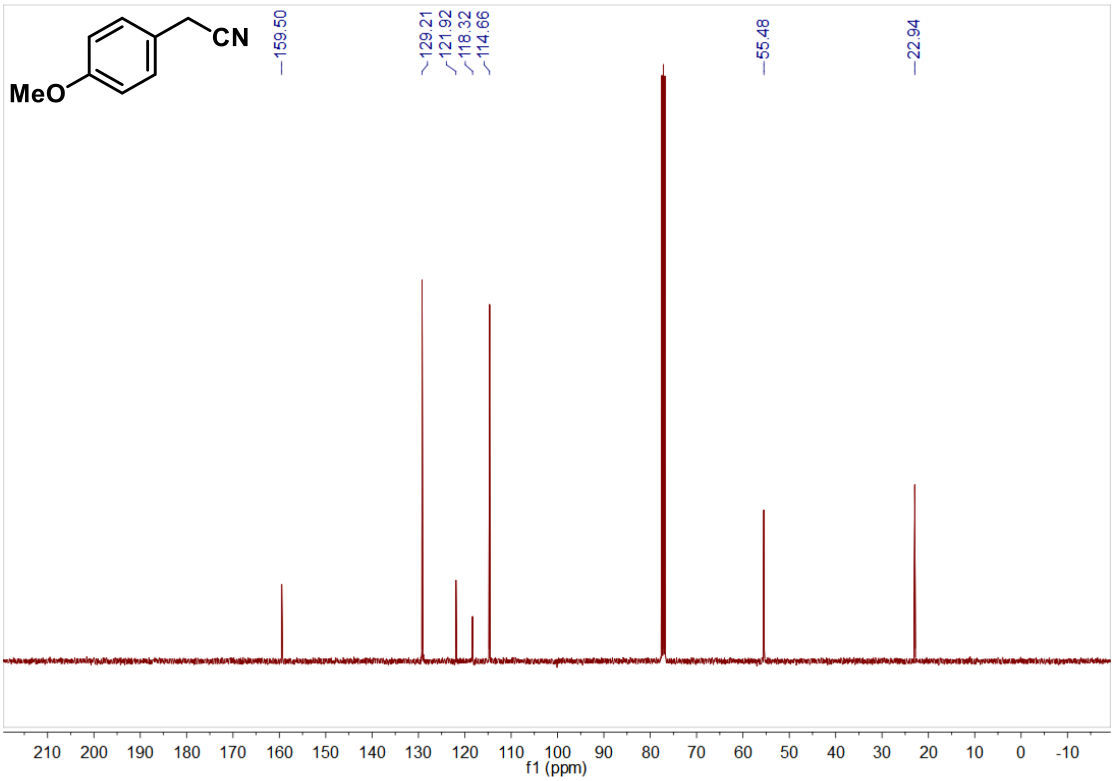

^{1}H NMR of compound 12 ($CDCl_3$, 500 MHz)

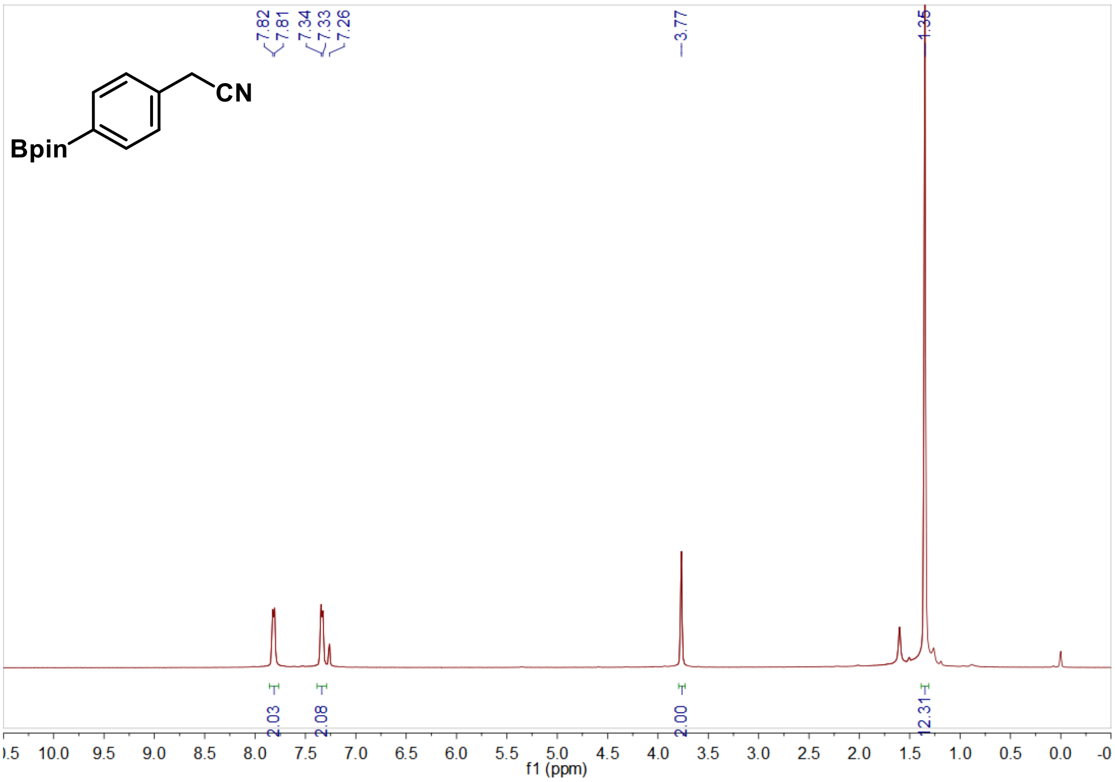

^{13}C NMR of compound 12 ($CDCl_3$, 126 MHz)

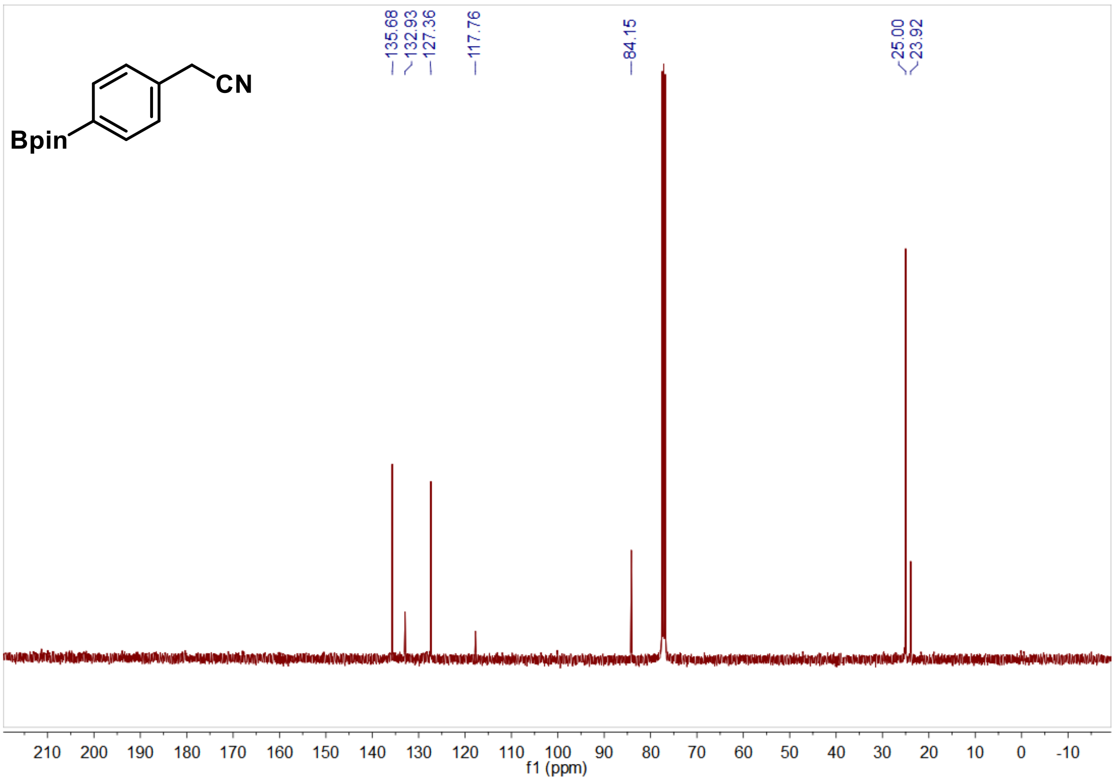

^{1}H NMR of compound 13 ($CDCl_3$, 400 MHz)

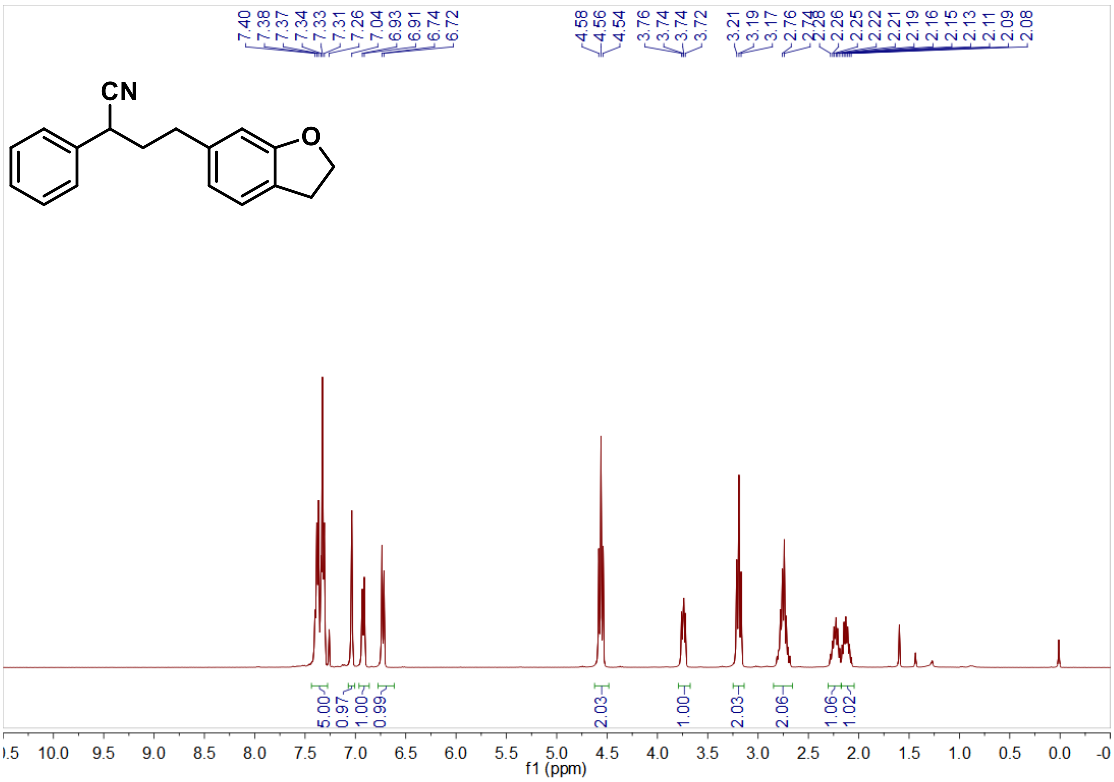

^{13}C NMR of compound 13 ($CDCl_3$, 101 MHz)

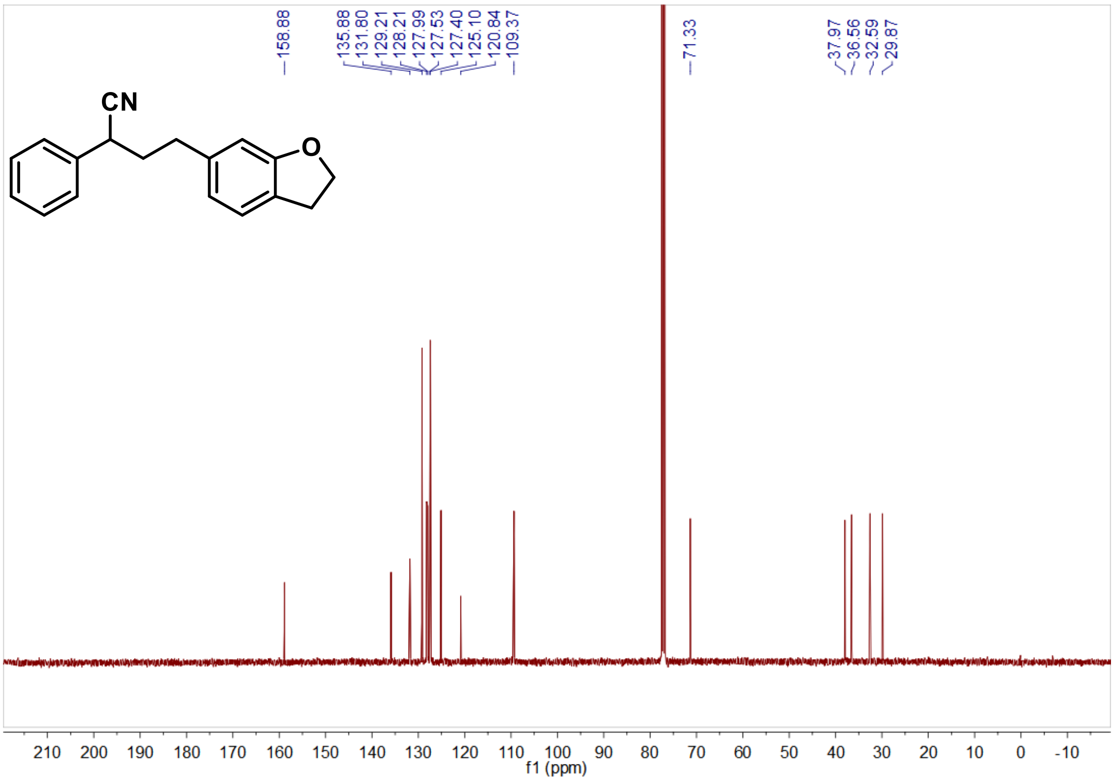

^{1}H NMR of compound 14 ($CDCl_3$, 400 MHz)

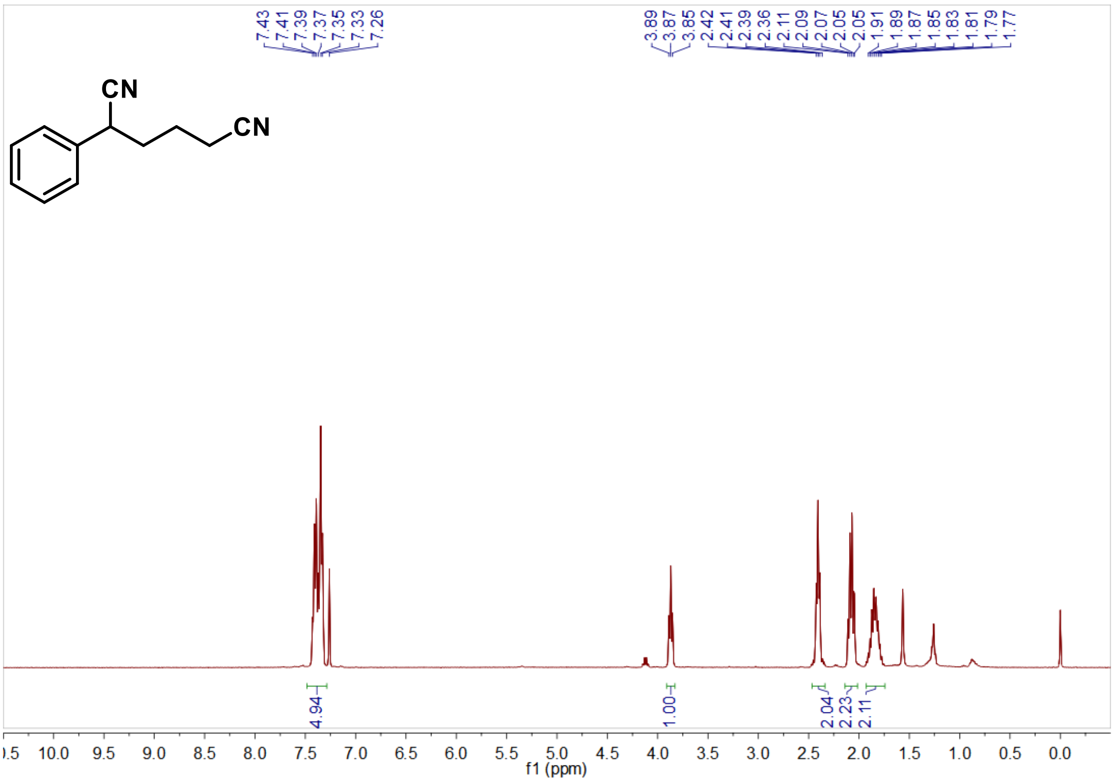

^{13}C NMR of compound 14 ($CDCl_3$, 101 MHz)

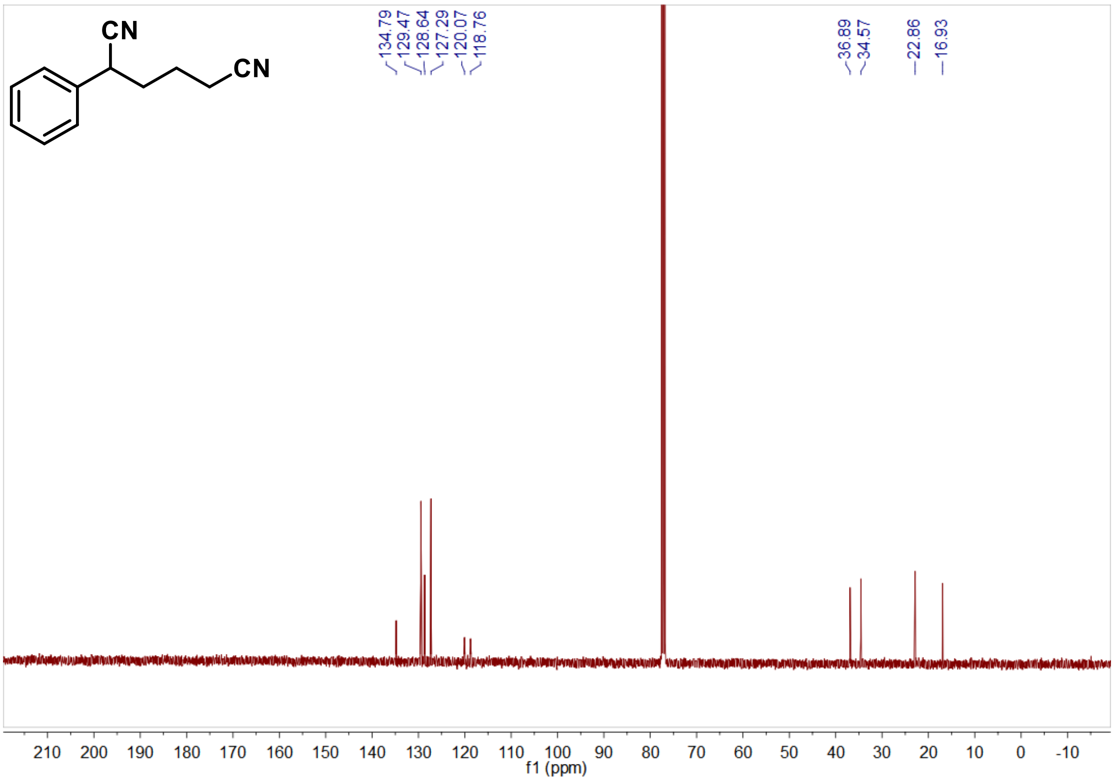

^{1}H NMR of compound 15 ($CDCl_3$, 400 MHz)

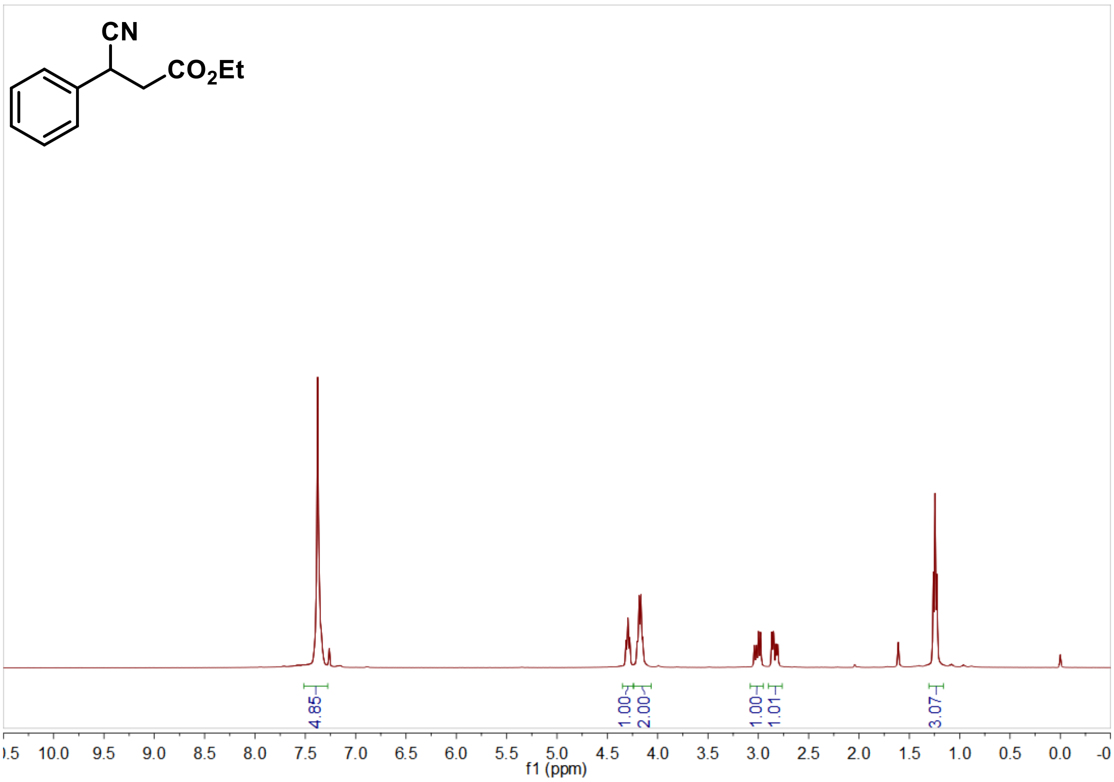

^{13}C NMR of compound 15 ($CDCl_3$, 101 MHz)

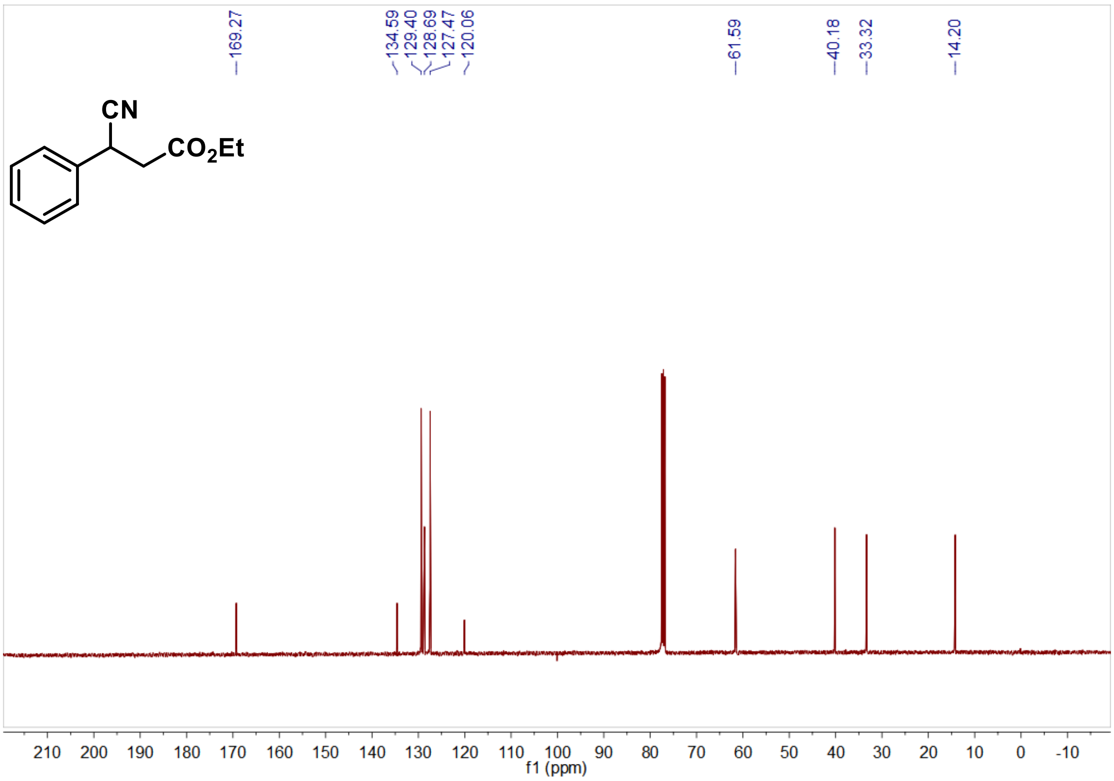

^{1}H NMR of compound 16 ($CDCl_3$, 400 MHz)

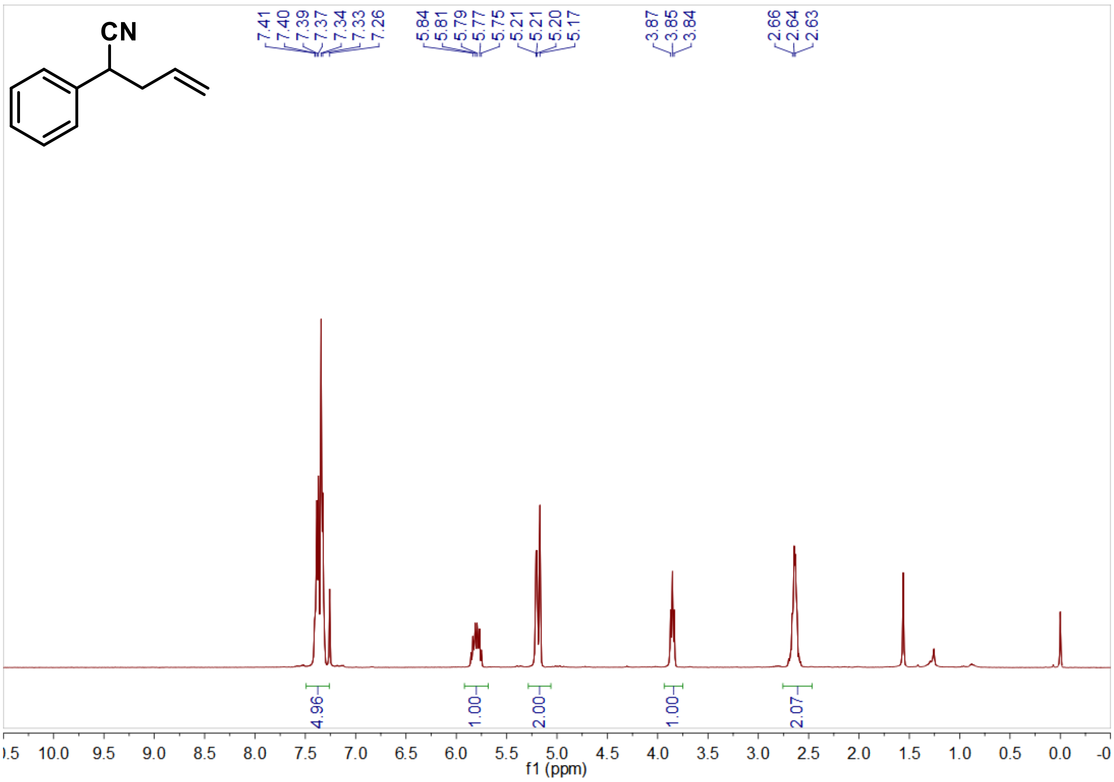

^{13}C NMR of compound 16 ($CDCl_3$, 101 MHz)

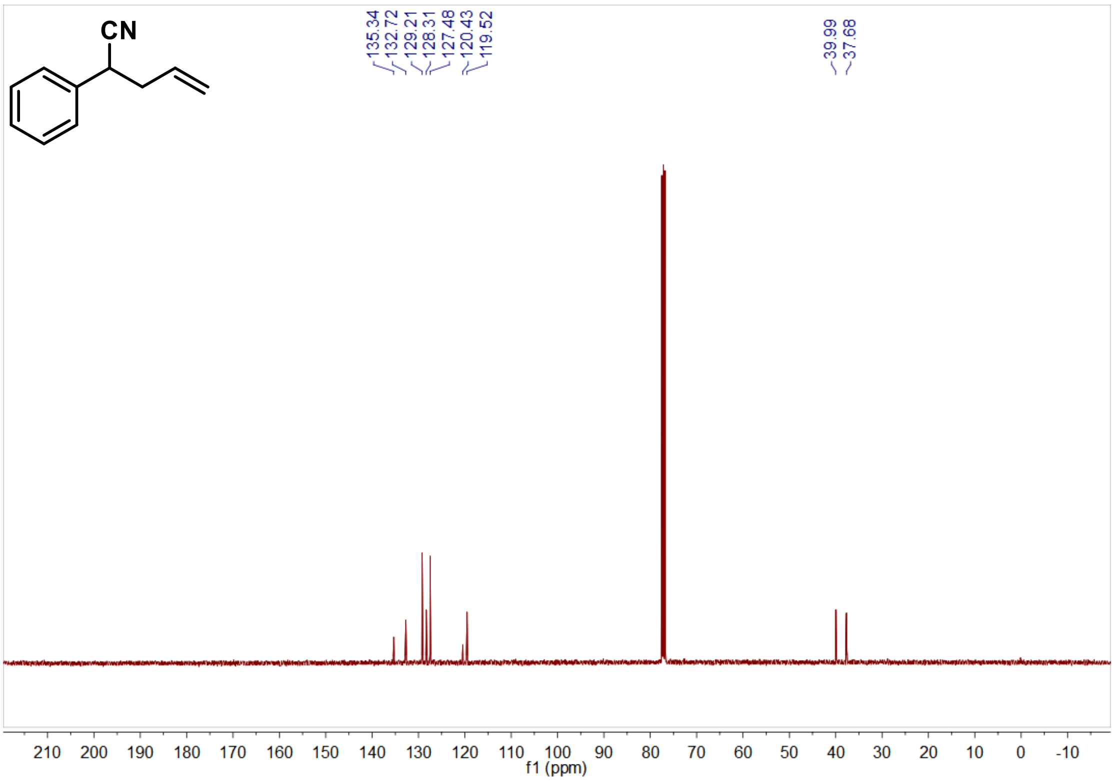

^{1}H NMR of compound 17 ($CDCl_3$, 400 MHz)

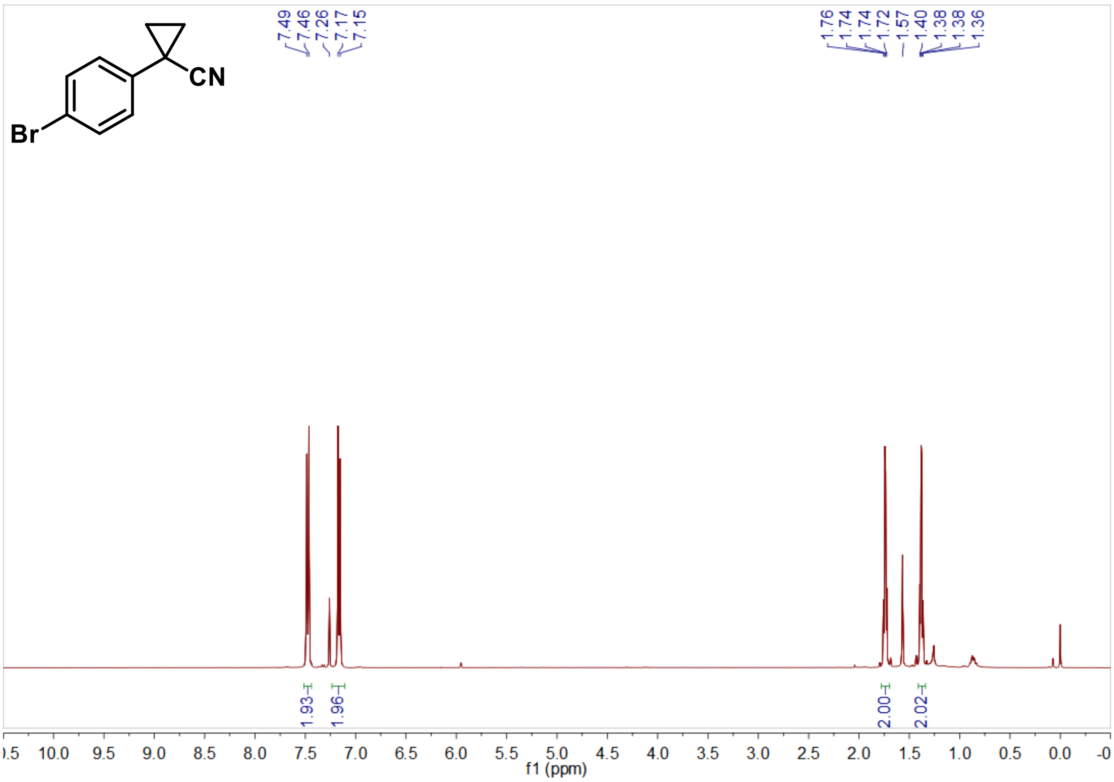

^{13}C NMR of compound 17 ($CDCl_3$, 101 MHz)

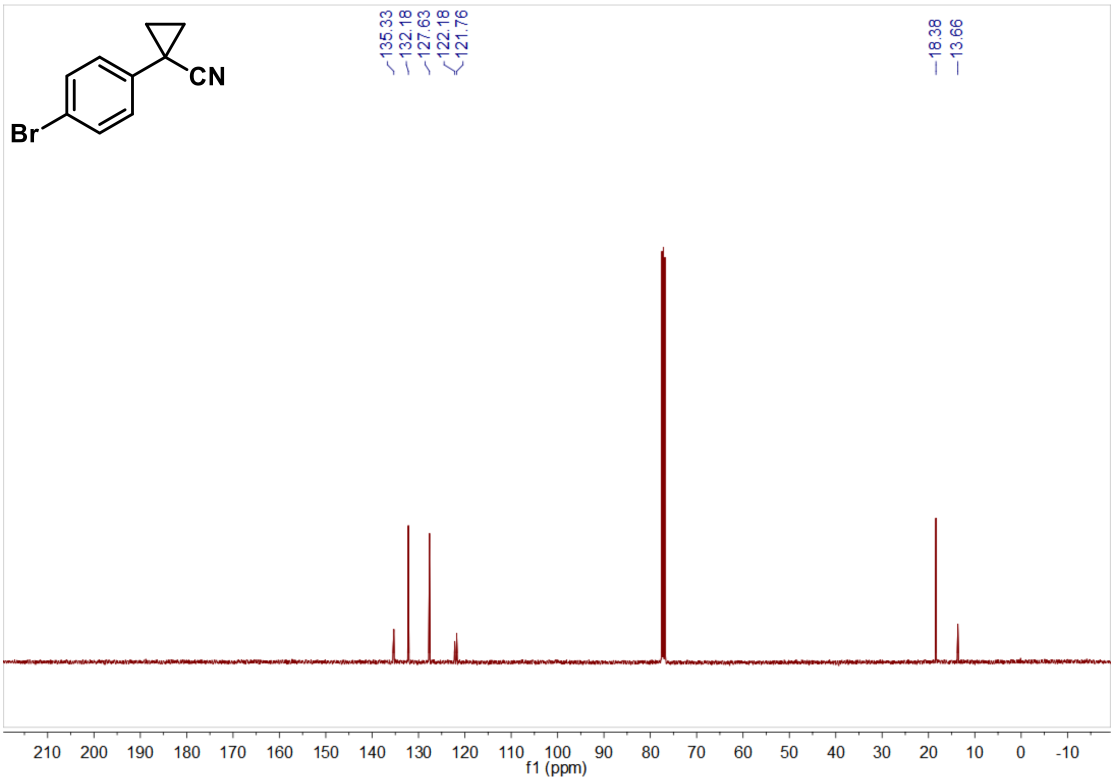

^{1}H NMR of compound 18 ($CDCl_3$, 400 MHz)

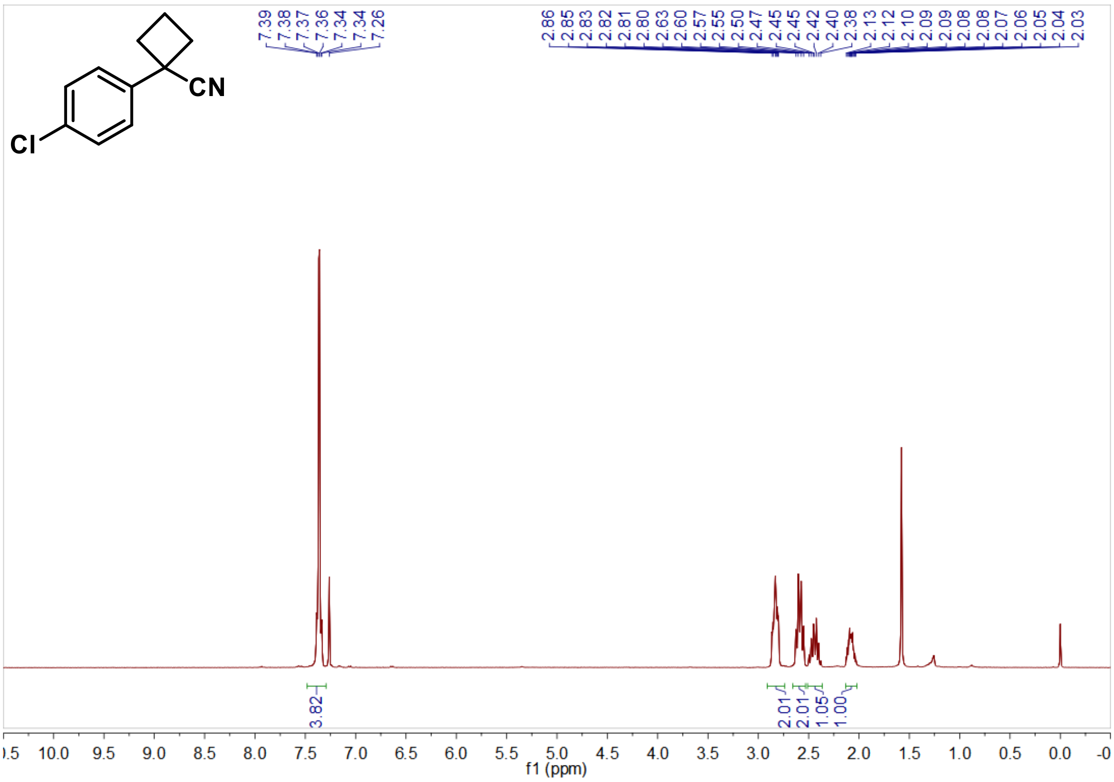

^{13}C NMR of compound 18 ($CDCl_3$, 101 MHz)

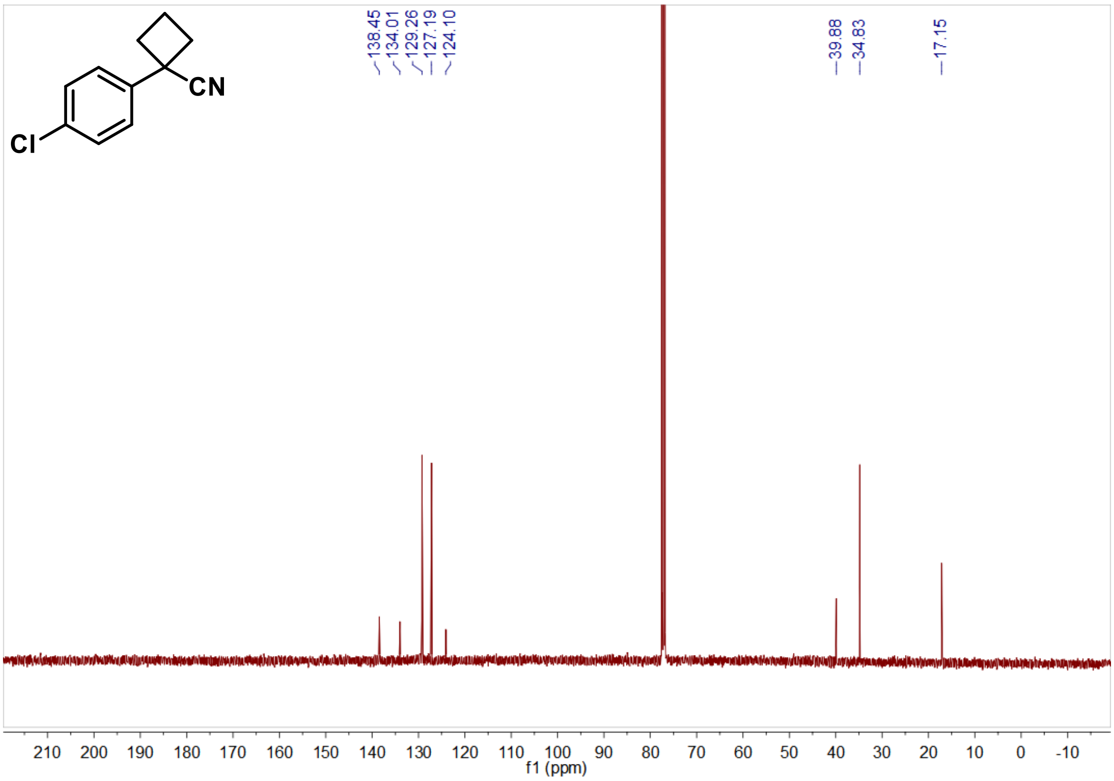

^{1}H NMR of compound 19 ($CDCl_3$, 400 MHz)

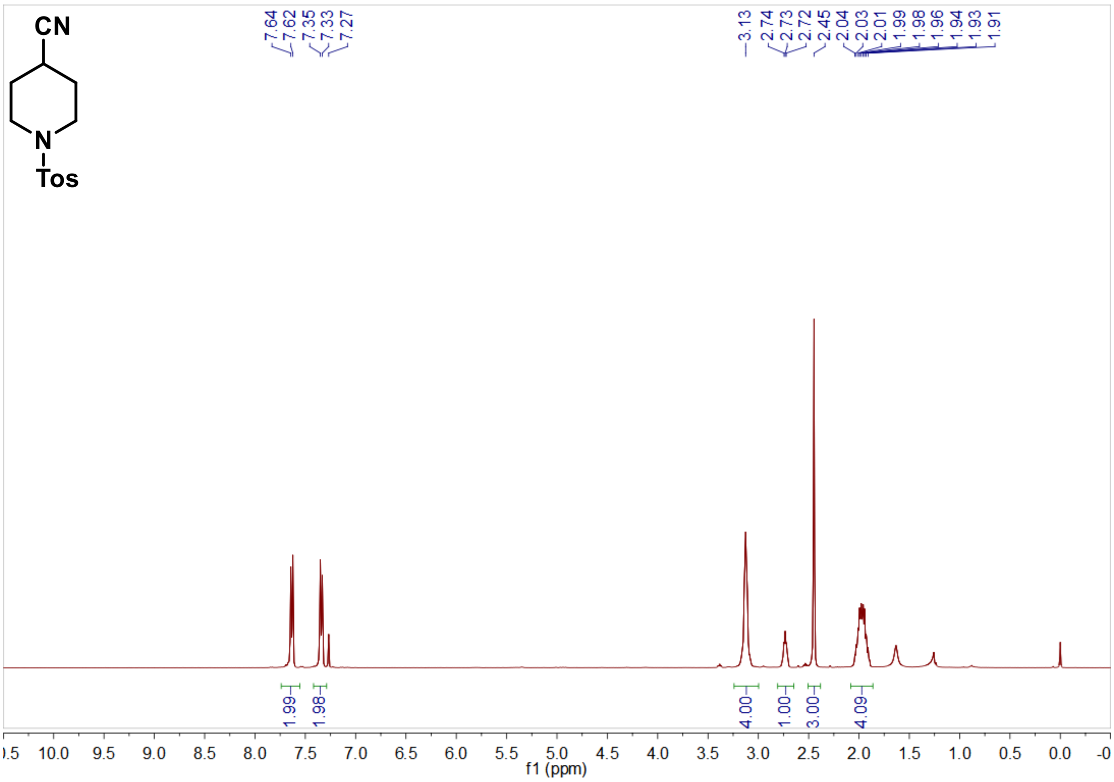

^{13}C NMR of compound 19 ($CDCl_3$, 101 MHz)

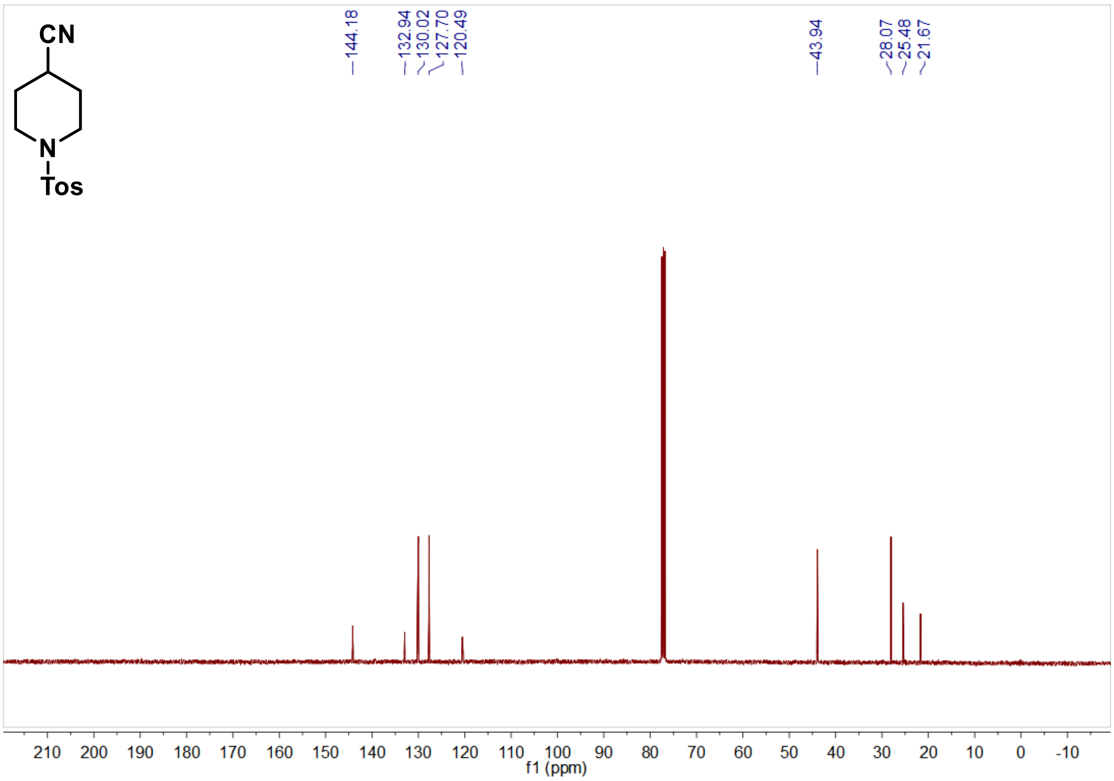

^{1}H NMR of compound 20 ($CDCl_3$, 400 MHz)

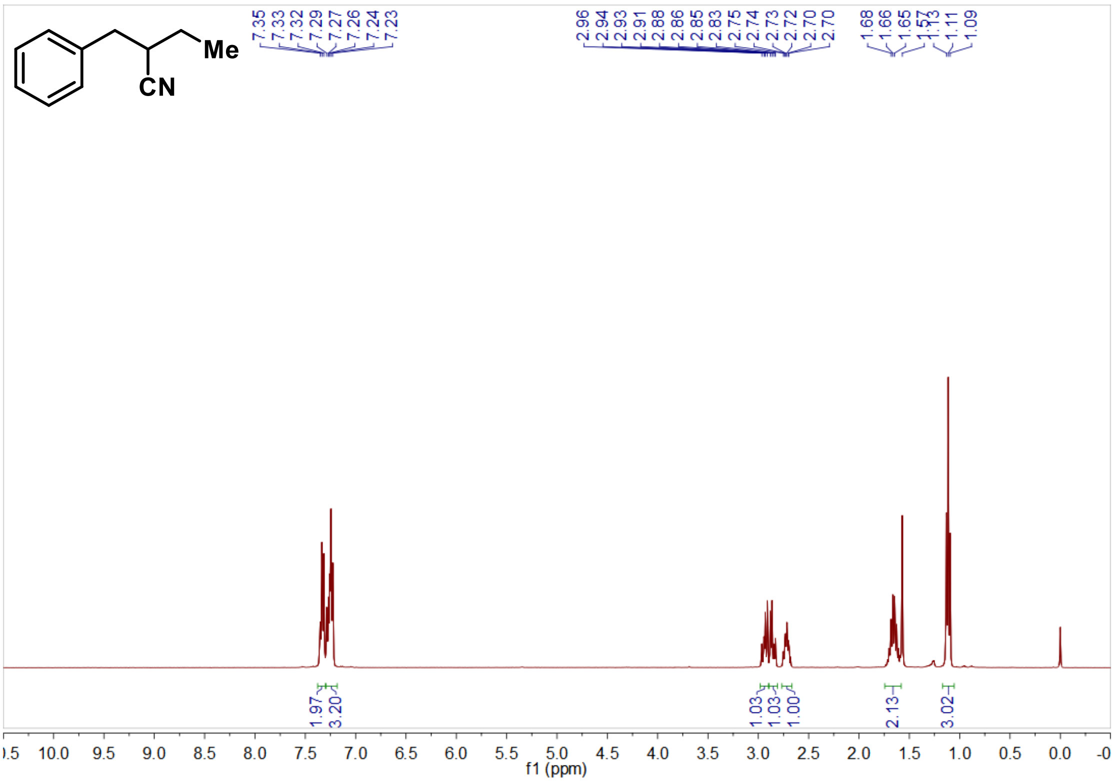

^{13}C NMR of compound 20 ($CDCl_3$, 101 MHz)

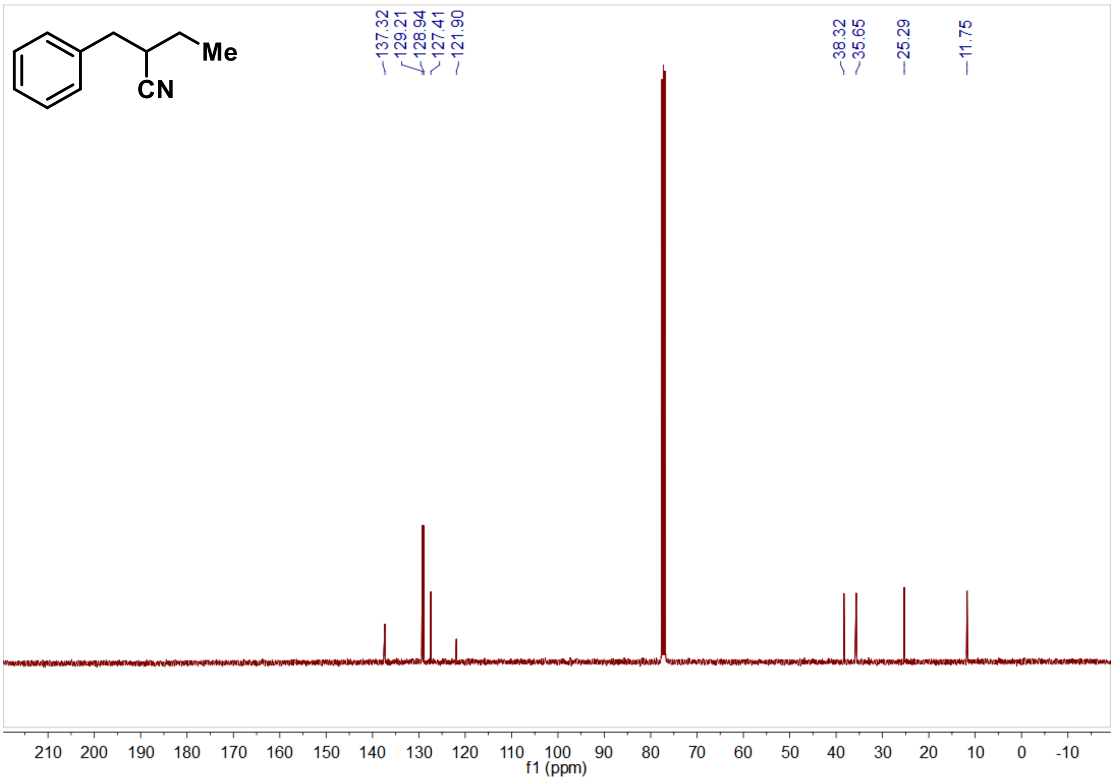

^{1}H NMR of compound 21 ($CDCl_3$, 400 MHz)

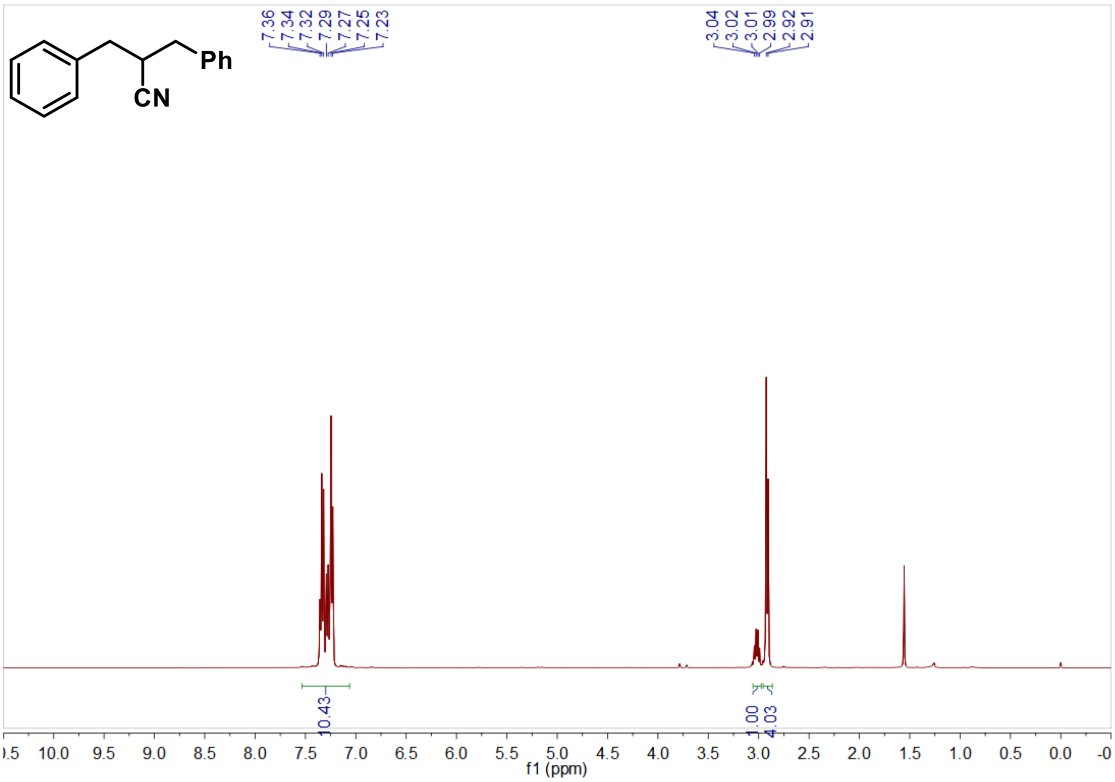

^{13}C NMR of compound 21 ($CDCl_3$, 101 MHz)

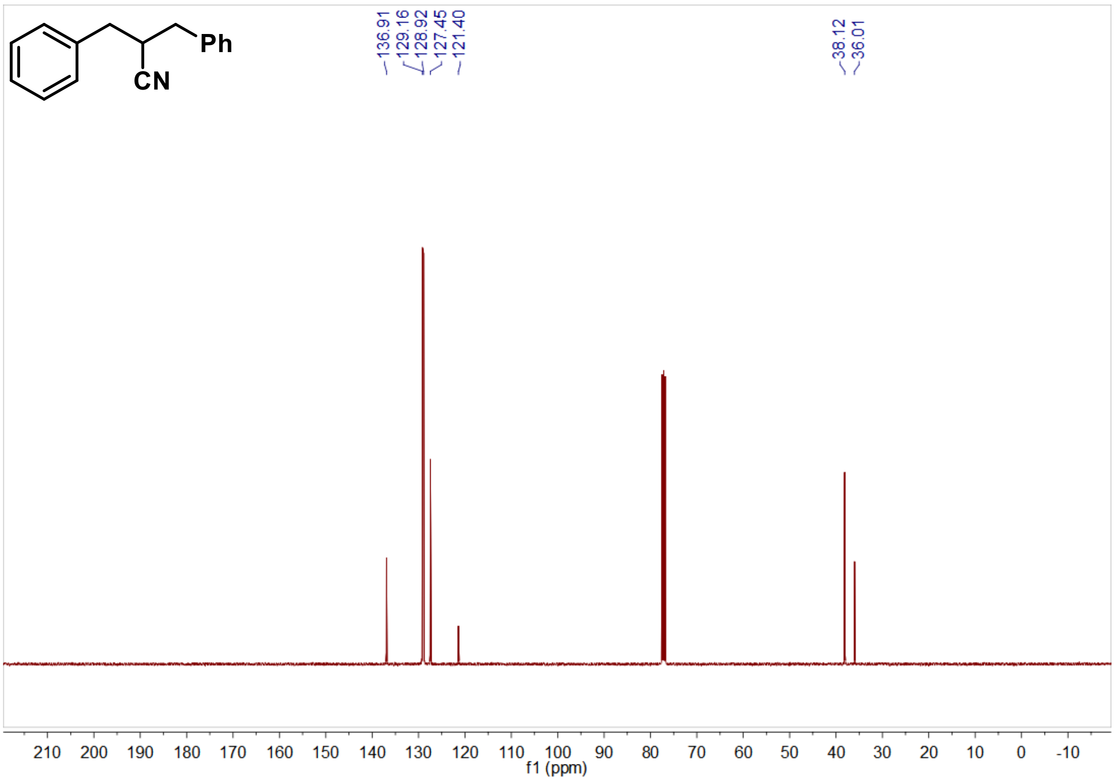

^{1}H NMR of compound 22 ($CDCl_3$, 400 MHz)

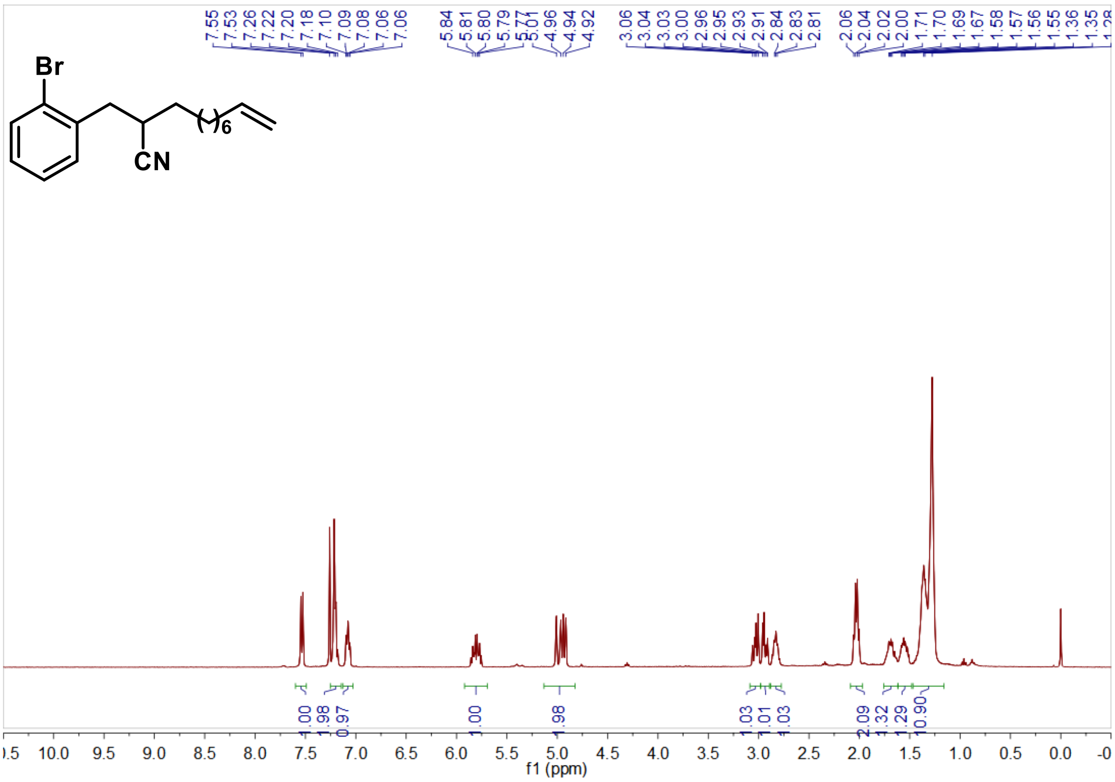

^{13}C NMR of compound 22 ($CDCl_3$, 101 MHz)

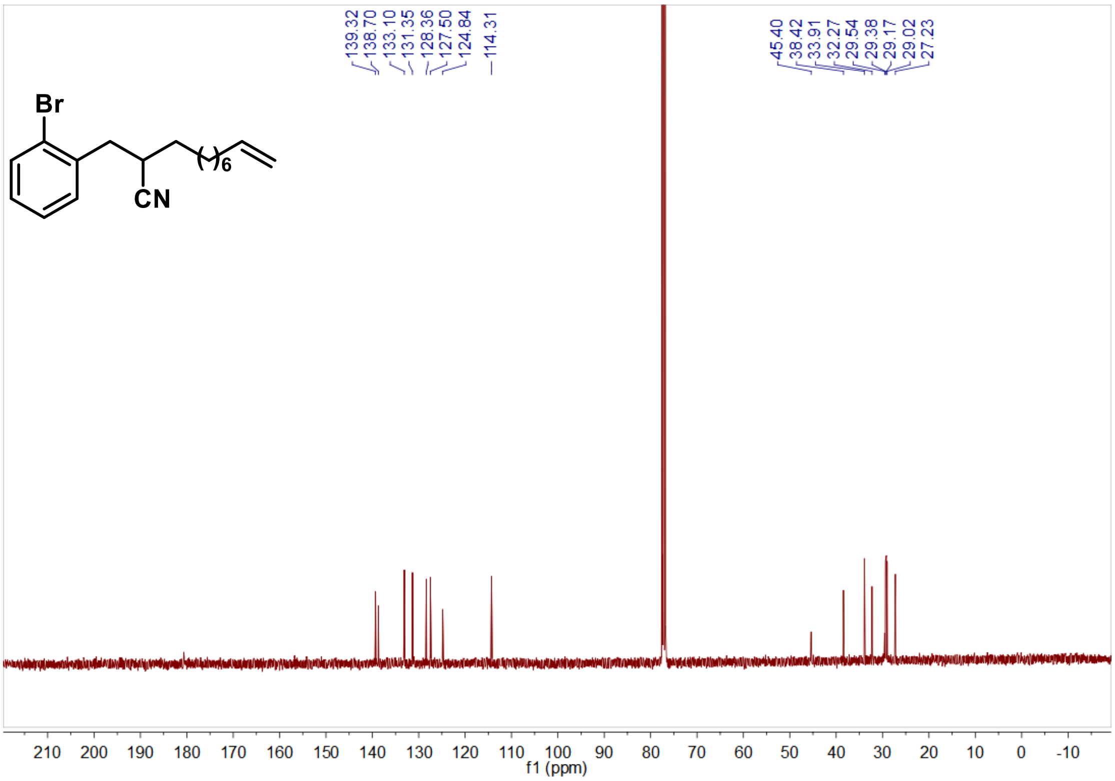

^{1}H NMR of compound 23 ($CDCl_3$, 400 MHz)

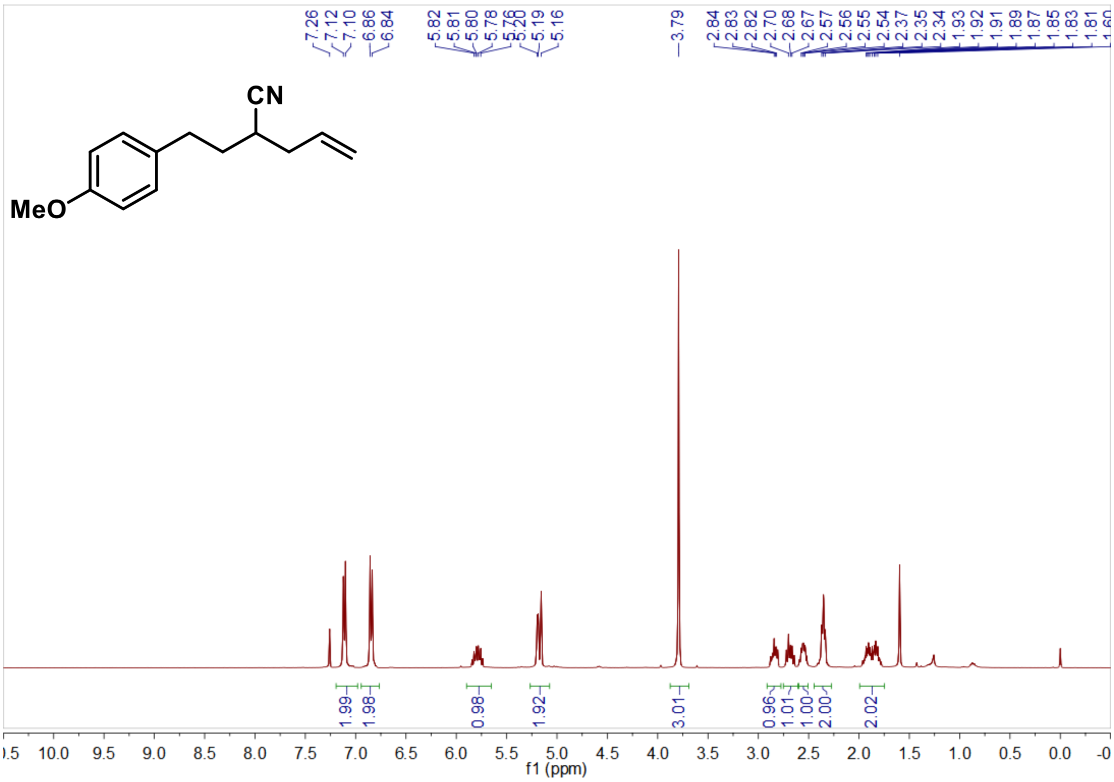

^{13}C NMR of compound 23 ($CDCl_3$, 101 MHz)

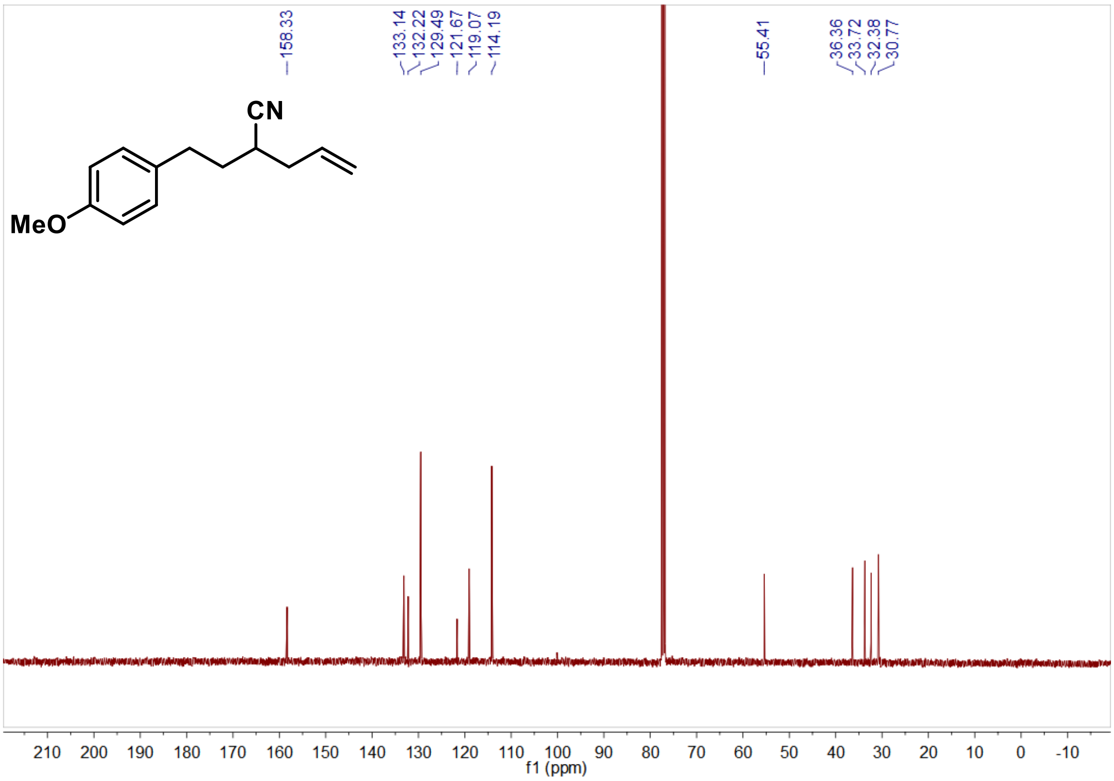

^{1}H NMR of compound 24 ($CDCl_3$, 400 MHz)

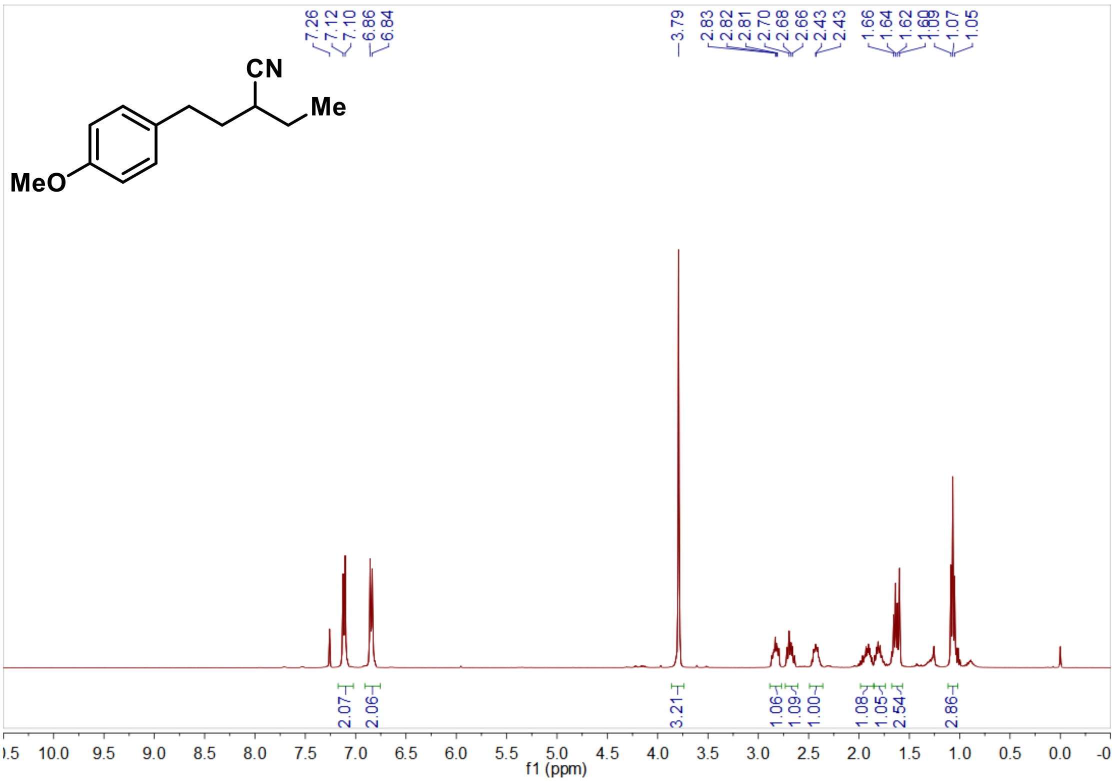

^{13}C NMR of compound 24 ($CDCl_3$, 101 MHz)

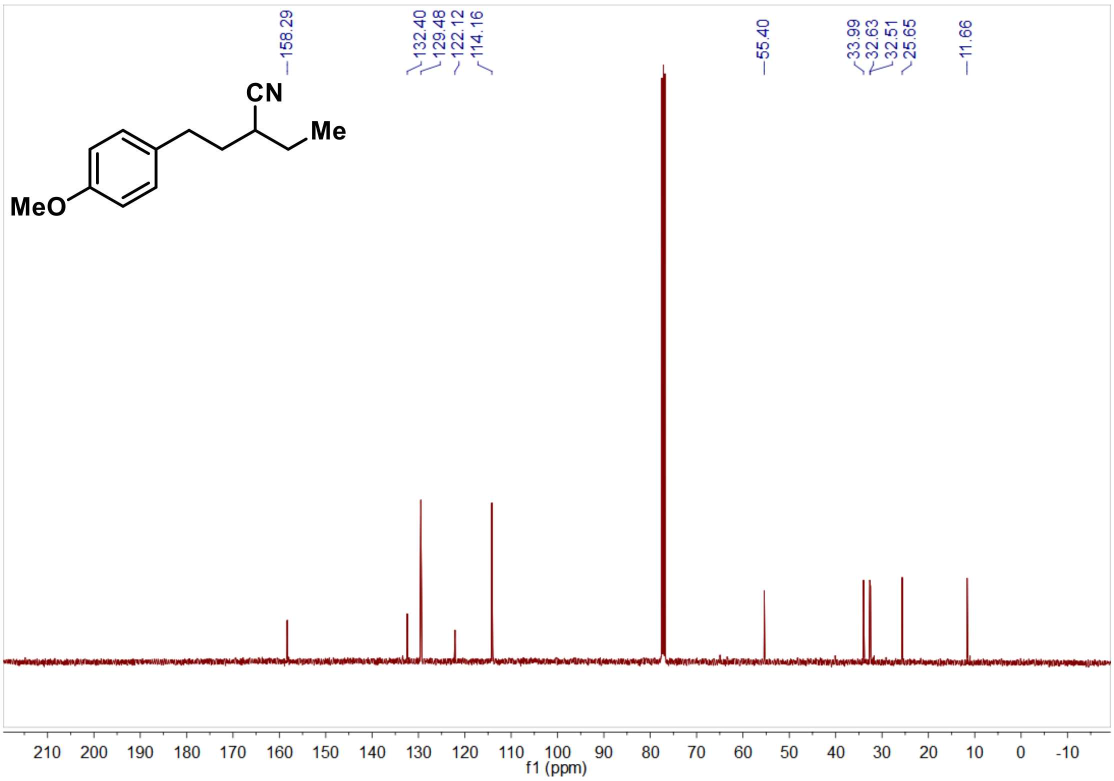

^{1}H NMR of compound 25 ($CDCl_3$, 400 MHz)

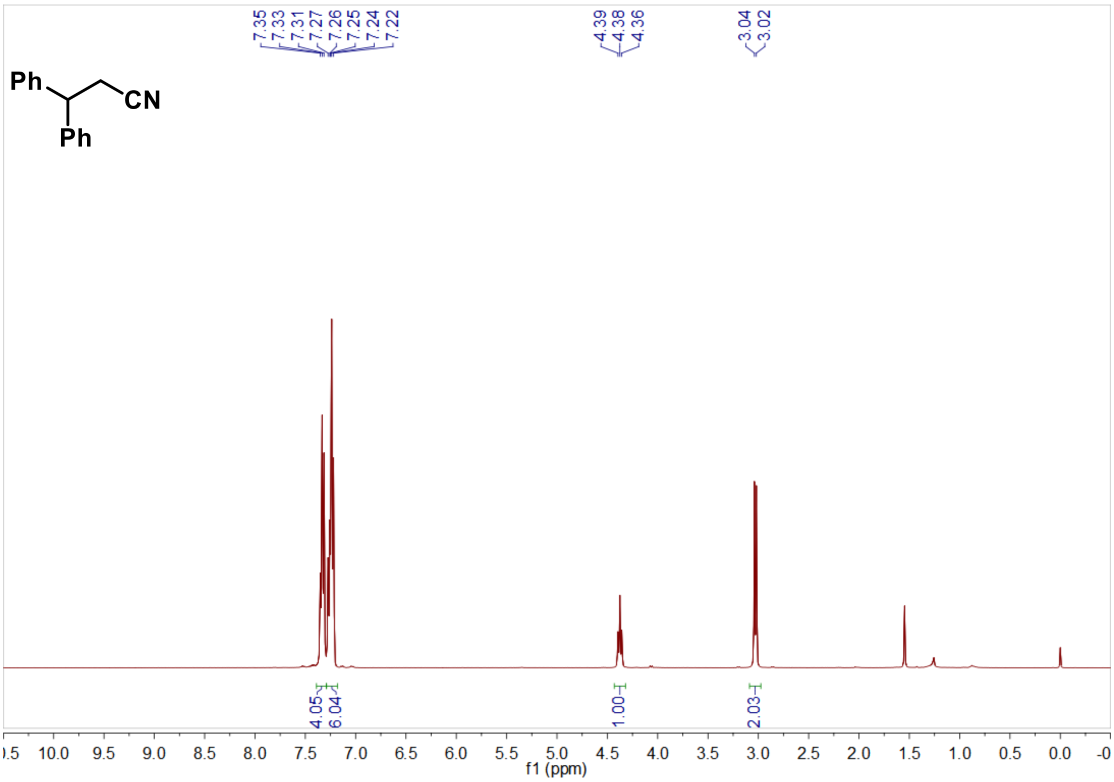

^{13}C NMR of compound 25 ($CDCl_3$, 101 MHz)

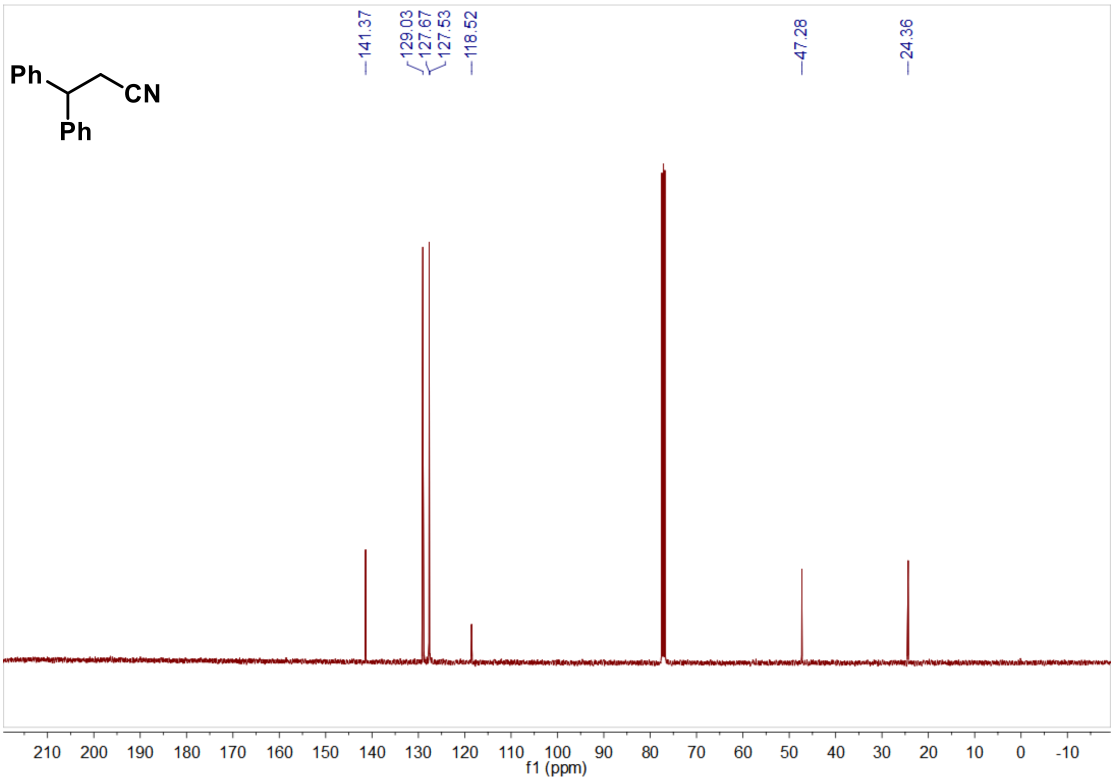

^{1}H NMR of compound 26 ($CDCl_3$, 400 MHz)

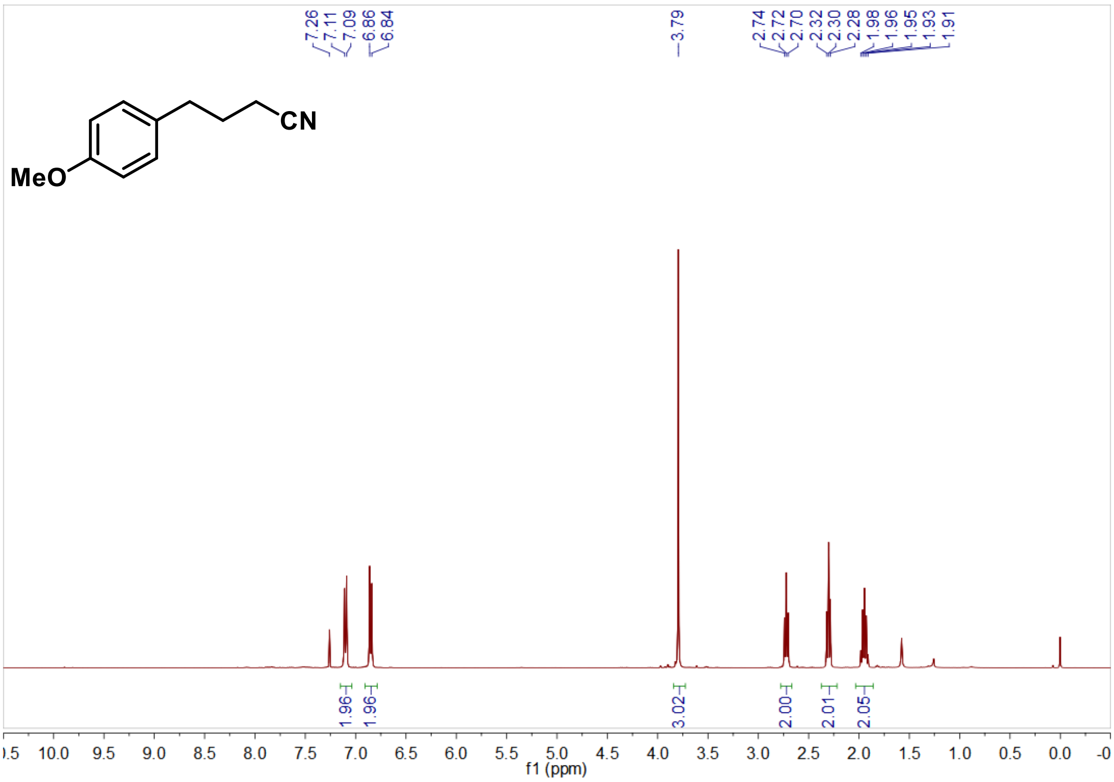

^{13}C NMR of compound 26 ($CDCl_3$, 101 MHz)

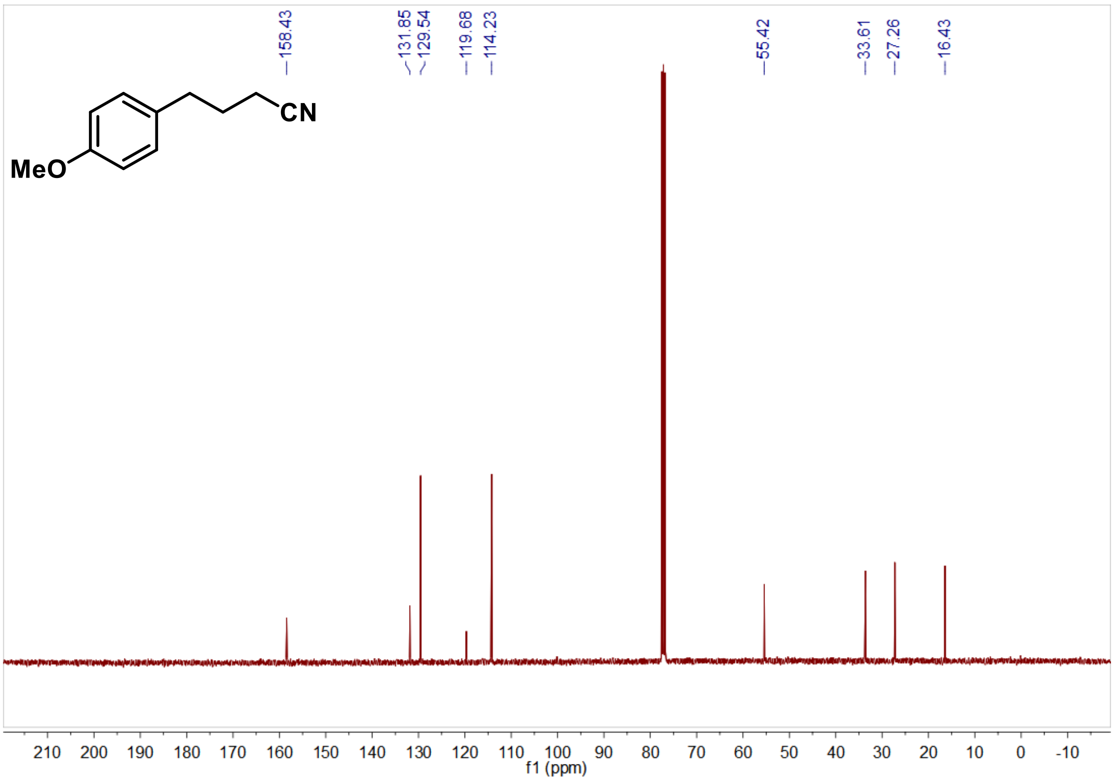

^{1}H NMR of compound 27 ($CDCl_3$, 500 MHz)

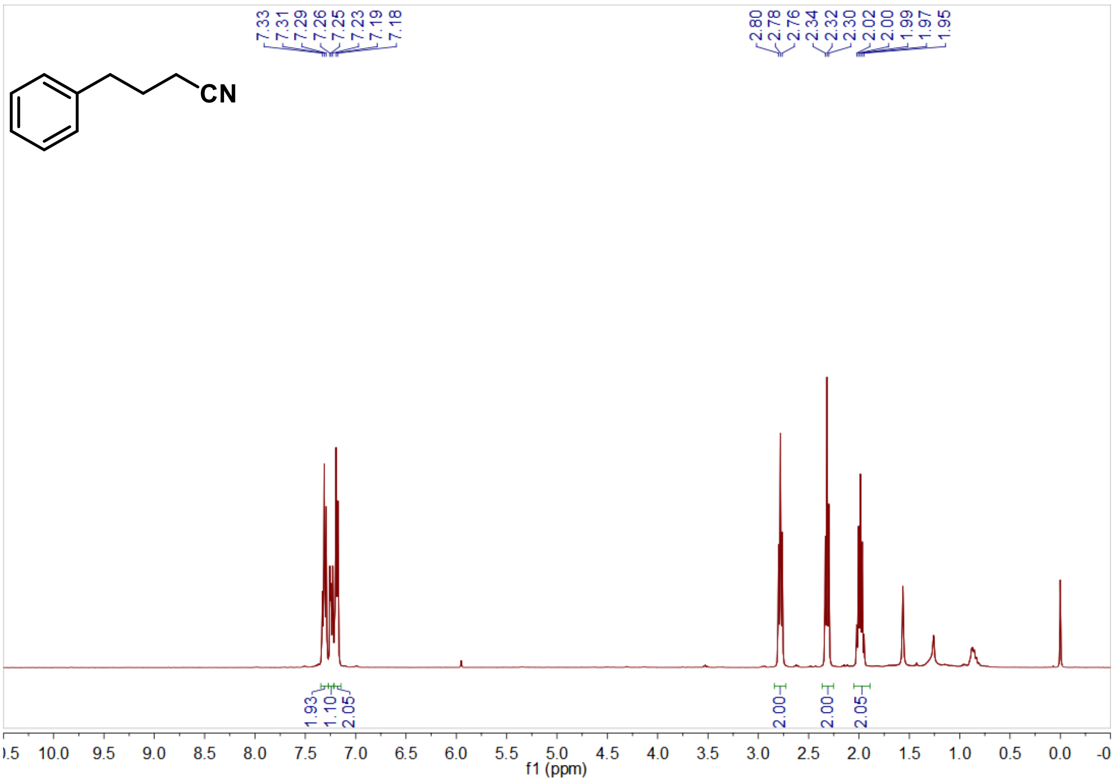

^{13}C NMR of compound 27 ($CDCl_3$, 126 MHz)

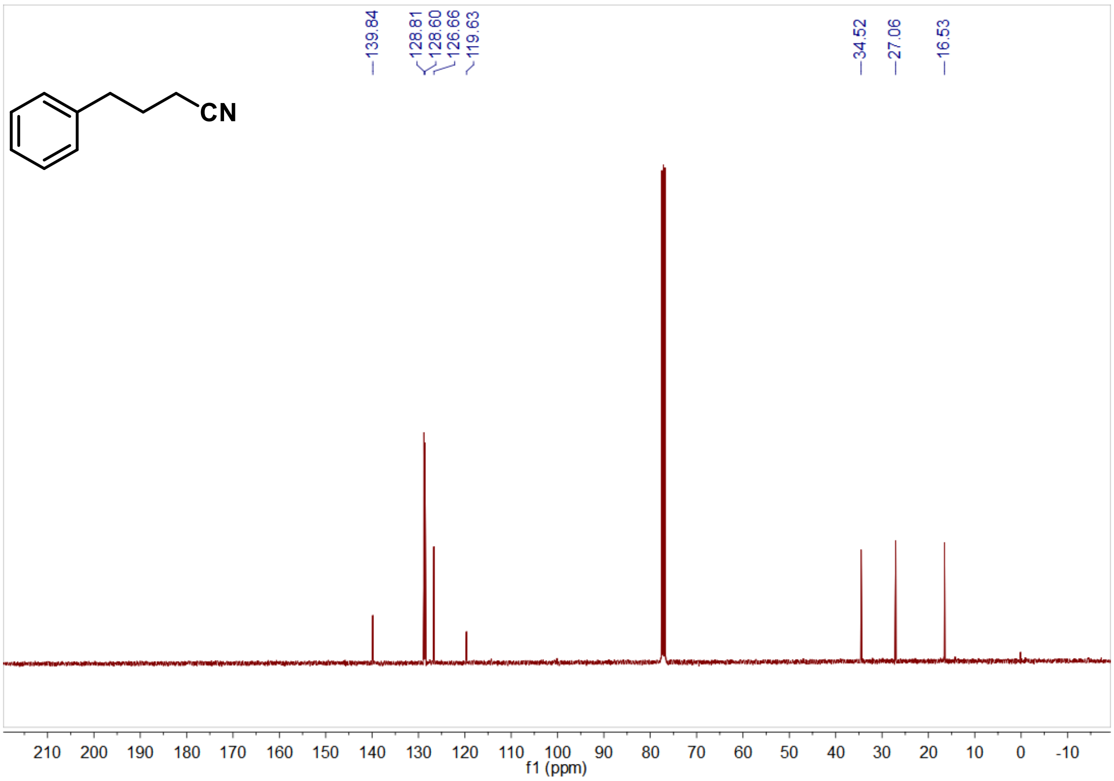

^{1}H NMR of compound 28 ($CDCl_3$, 400 MHz)

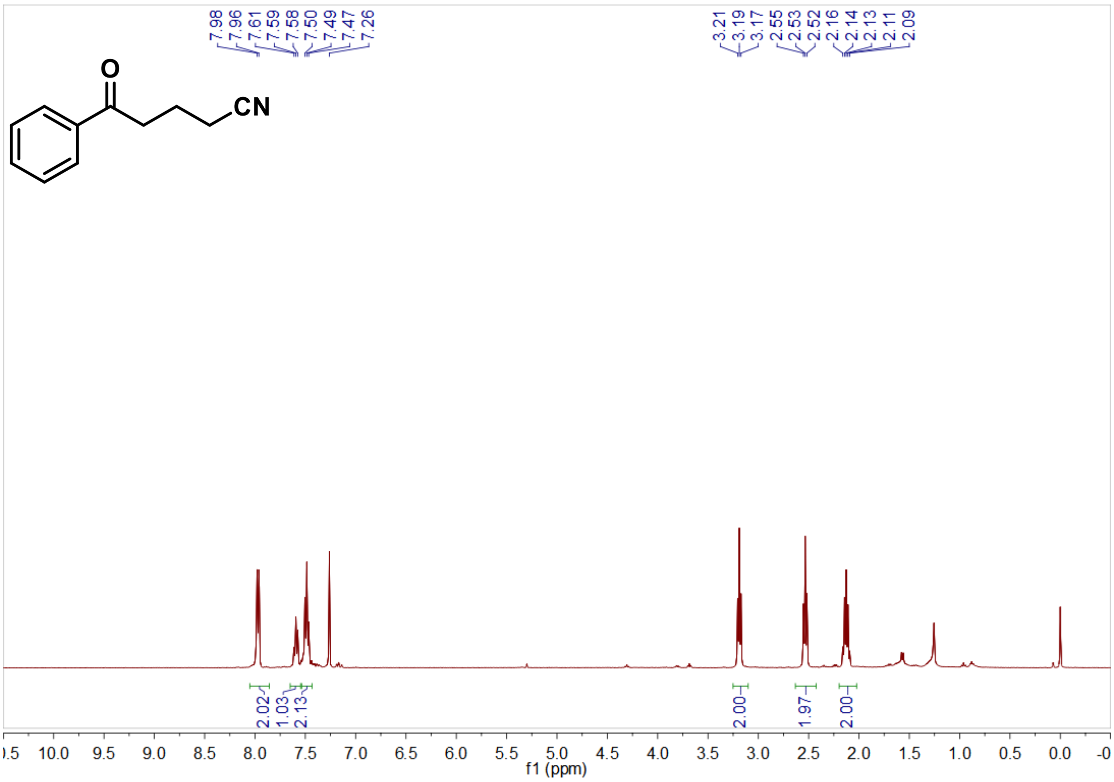

^{13}C NMR of compound 28 ($CDCl_3$, 101 MHz)

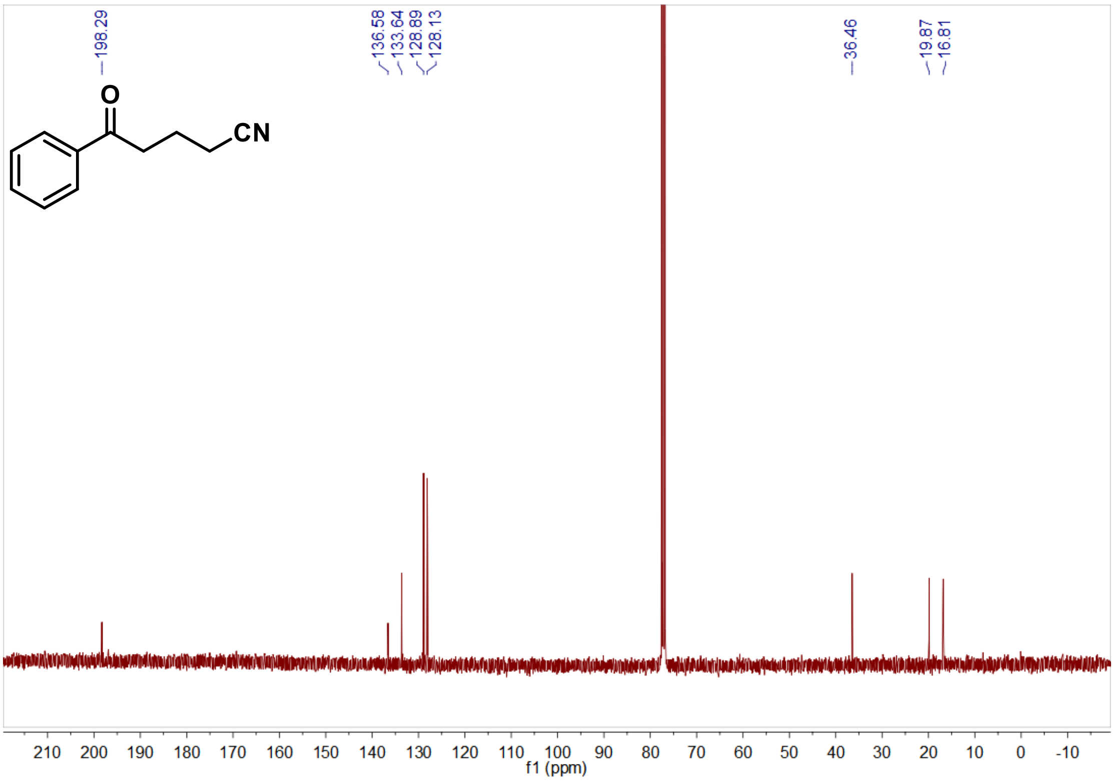

^{1}H NMR of compound 29 ($CDCl_3$, 400 MHz)

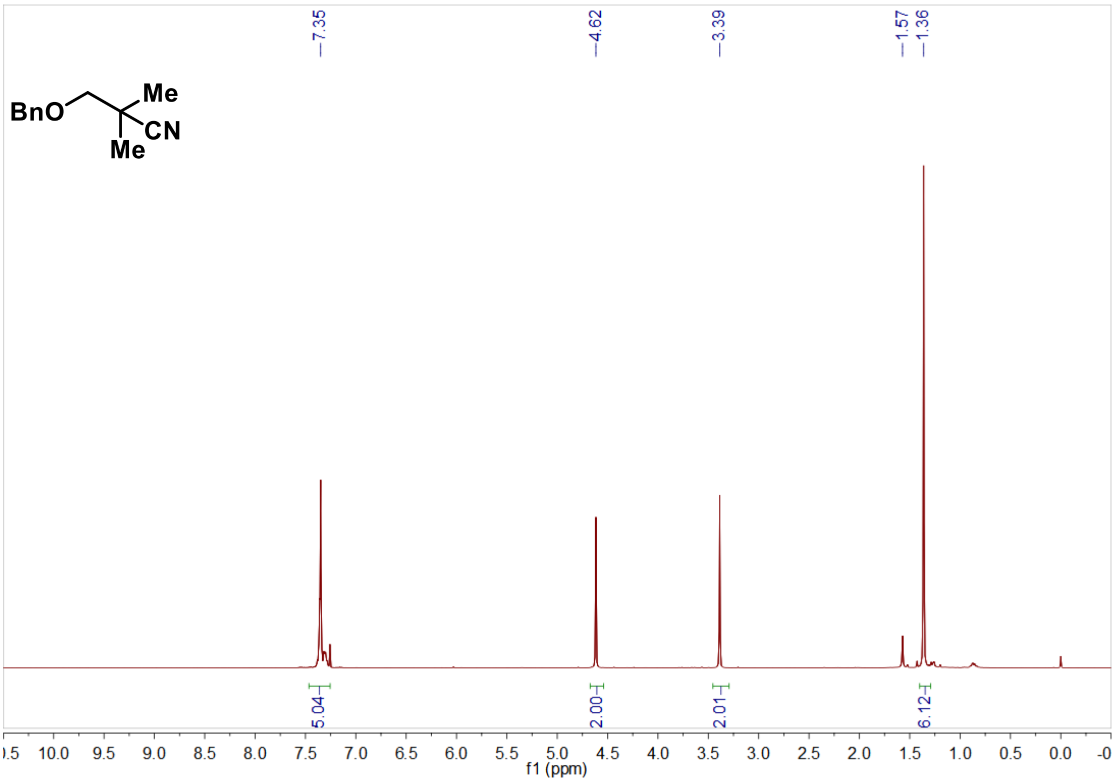

^{13}C NMR of compound 29 ($CDCl_3$, 101 MHz)

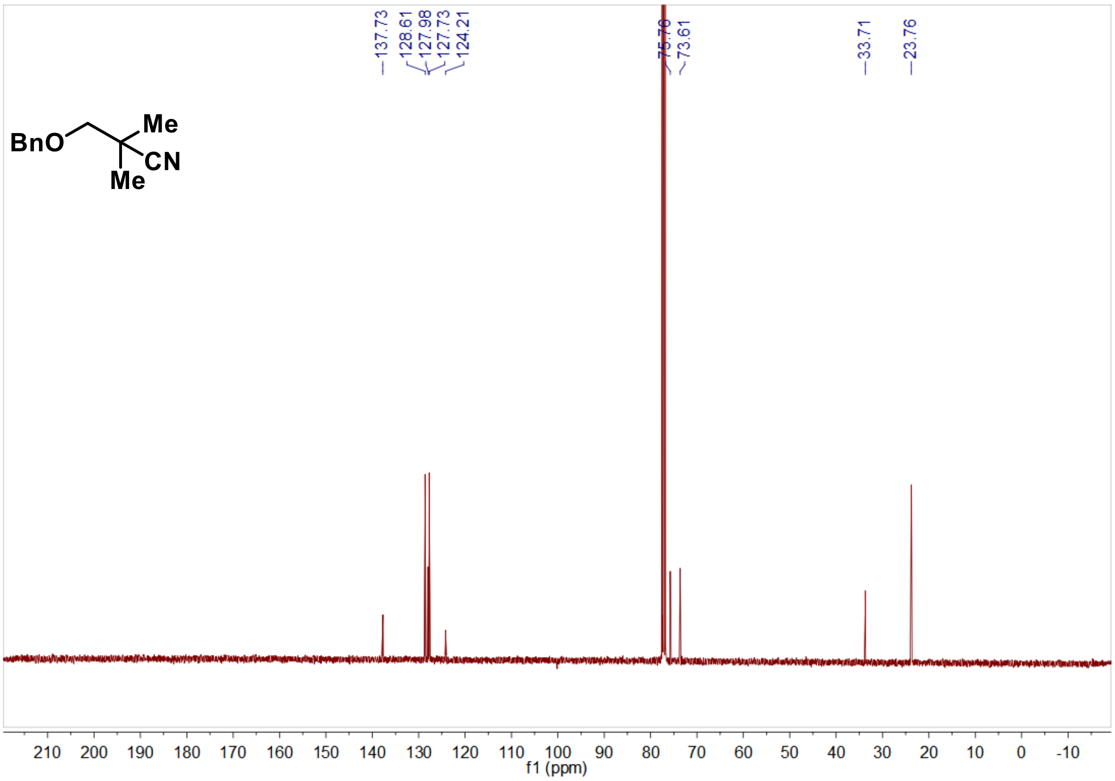

^{1}H NMR of compound 30 ($CDCl_3$, 500 MHz)

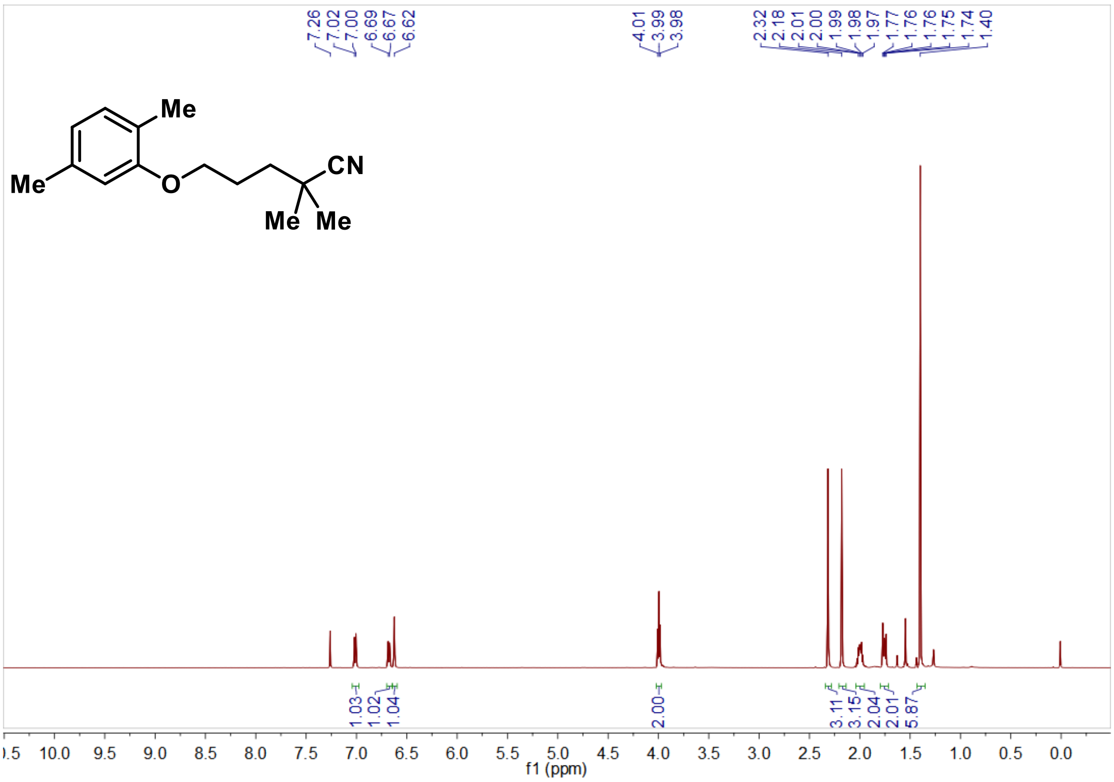

^{13}C NMR of compound 30 ($CDCl_3$, 126 MHz)

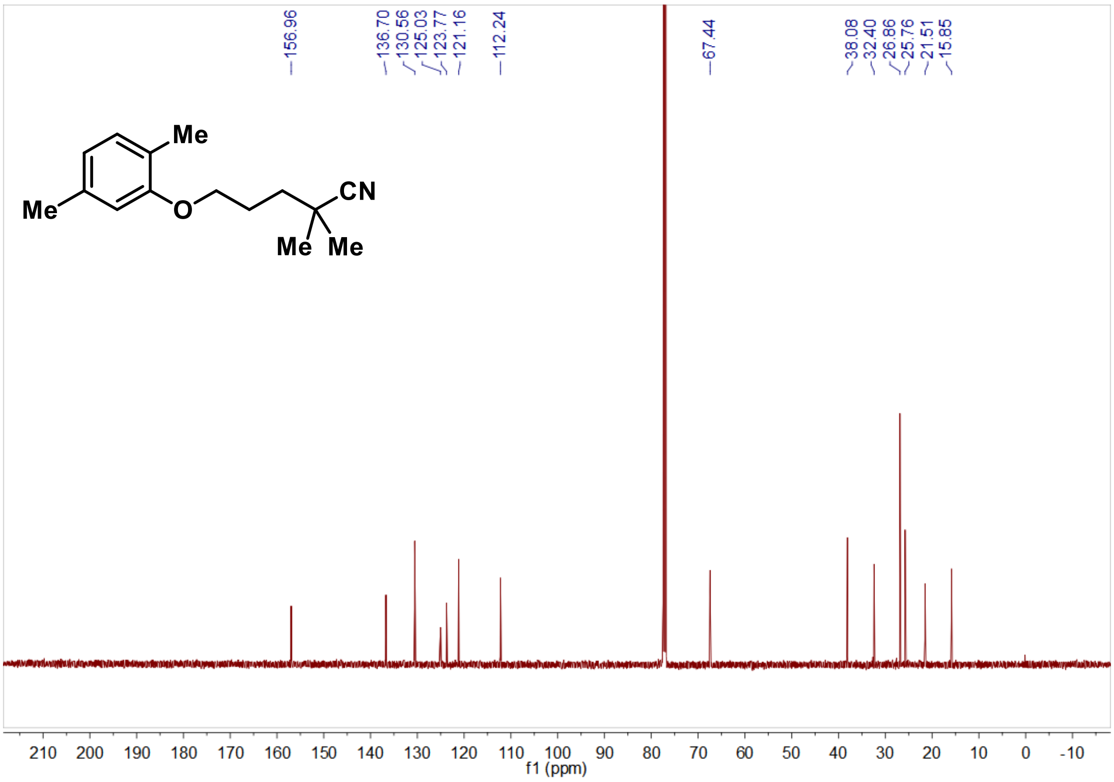

^{1}H NMR of compound 32 ($CDCl_3$, 400 MHz)

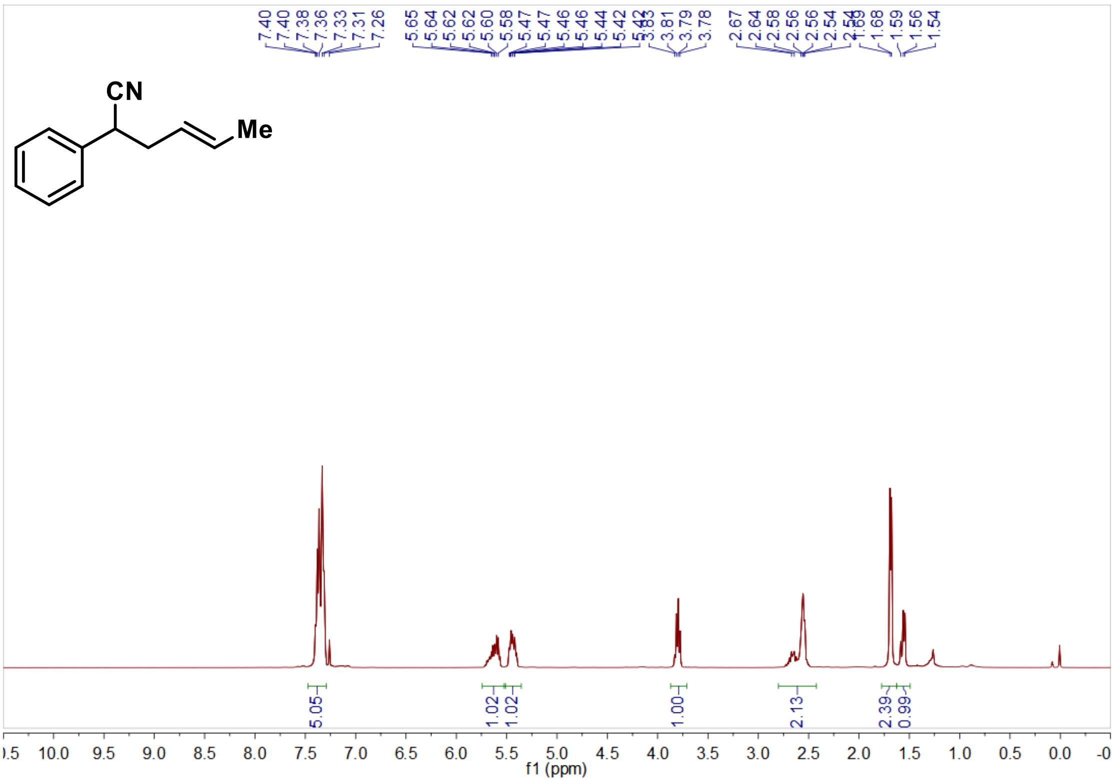

^{13}C NMR of compound 32 ($CDCl_3$, 101 MHz)

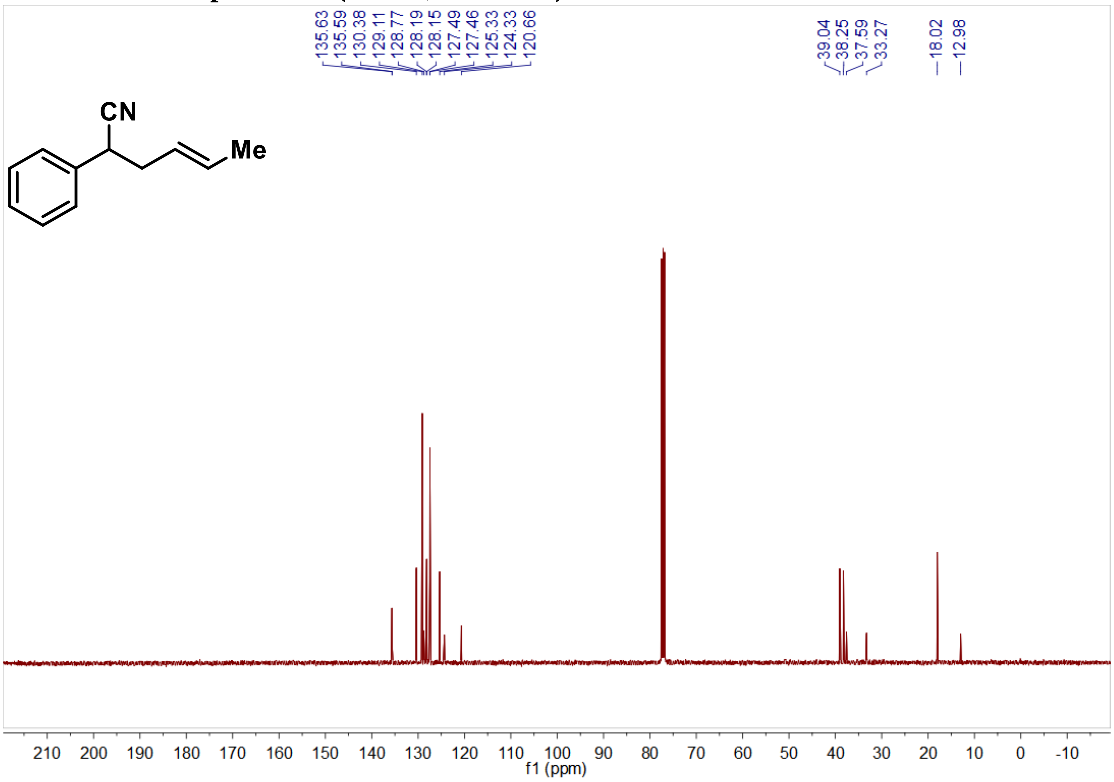

Supplement: File 1 — Experimental procedures, mechanistic studies, analytical data and copies of NMR spectra. [file Beilstein_J_Org_Chem-20-1497-s001.pdf]
